# Programmable Multiplexed Nucleic Acid Detection by Harnessing Specificity Defect of CRISPR‐Cas12a

**DOI:** 10.1002/advs.202411021

**Published:** 2024-12-04

**Authors:** Xin Guan, Rui Yang, Jiongyu Zhang, Jeong Moon, Chengyu Hou, Chong Guo, Lori Avery, Danielle Scarola, Daniel S. Roberts, Rocco LaSala, Changchun Liu

**Affiliations:** ^1^ Department of Biomedical Engineering University of Connecticut Health Center Farmington Connecticut 06030 USA; ^2^ Department of Biomedical Engineering University of Connecticut Storrs Connecticut 06269 USA; ^3^ Department of Pathology and Laboratory Medicine University of Connecticut Health Center Farmington Connecticut 06030 USA; ^4^ Division of Otolaryngology‐Head & Neck Surgery University of Connecticut Health Center Farmington Connecticut 06030 USA

**Keywords:** approximate pattern matching, CRISPR‐Cas12a, HR‐HPV detection, paper‐based microfluidic chip, precise querying

## Abstract

CRISPR‐Cas12a works like a sophisticated algorithm in nucleic acid detection, yet its challenge lies in sometimes failing to distinguish targets with mismatches due to its specificity limitations. Here, the mismatch profiles, including the quantity, location, and type of mismatches in the CRISPR‐Cas12a reaction, are investigated and its various tolerances to mismatches are discovered. By harnessing the specificity defect of the CRISPR‐Cas12a enzyme, a dual‐mode detection strategy is designed, which includes approximate matching and precise querying of target sequences and develop a programmable multiplexed nucleic acid assay. With the assay, 14 high‐risk human papillomavirus (HPV) subtypes are simultaneously detected, collectively responsible for 99% of cervical cancer cases, with attomolar sensitivity. Specifically, the assay not only distinguishes HPV16 and HPV18, the two most common subtypes but also detects 12 other high‐risk pooled HPV subtypes. To enable low‐cost point‐of‐care testing, the assay is incorporated into a paper‐based microfluidic chip. Furthermore, the clinical performance of the paper‐based microfluidic chip is validated by testing 75 clinical swab samples, achieving performance comparable to that of PCR. This programmable multiplexed nucleic acid assay has the potential to be widely applied for sensitive, specific, and simultaneous detection of different pathogens.

## Graphical abstract

A CRISPR‐Cas12a‐based dual‐mode assay strategy is developed for multiplexed nucleic acid detection by incorporating precise querying and approximate pattern matching. With the assay, HPV16 and HPV18 are not only distinguished but also 12 other high‐risk pooled HPV subtypes are detected. The assay into a paper‐based microfluidic chip is integrated and its clinical utility with 75 clinical samples, demonstrating significant promise for simple, sensitive, specific, and multiplexed detection of pathogens at the point of care is validated.

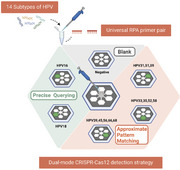

## 1 Introduction

Accurate detection of nucleic acids is essential for the diagnosis and management of numerous diseases. While polymerase chain reaction (PCR) has long been considered the gold standard due to its high sensitivity and specificity, its dependency on sophisticated infrastructure and technical expertise renders it impractical in resource‐limited environments. Recently, the CRISPR‐Cas system has emerged as a promising alternative to PCR assays due to its simplicity, rapidity, and minimal equipment requirements. Specifically, Cas12a, a Class II CRISPR‐associated endonuclease,^[^ ^1^ ^]^ has proven to be a pivotal enzyme in nucleic acid‐based diagnostics. It is activated by specific target recognition, leading to *trans*‐cleavage, a process utilized in clinical assays where it cleaves fluorophore‐quencher‐tagged single‐stranded DNA (ssDNA) to produce a fluorescent signal for sample analysis. In this scenario, CRISPR‐Cas12a functions akin to a database search engine, with the protospacer‐adjacent motif (PAM) sequence acting as the query index. When the target sequence matches the CRISPR RNA (crRNA) spacer sequence, a fluorescence signal is generated, effectively indicating a match in the database query.

To further improve the detection sensitivity of the CRISPR‐Cas12a system, various isothermal amplification techniques, such as recombinase polymerase amplification (RPA) and loop‐mediated isothermal amplification, have been integrated with CRISPR‐Cas12a to amplify signals. Particularly, RPA, driven by a recombinase‐primer complex, stands out for its ability to exponentially amplify target segments at a constant temperature (37–42 °C) within 20 min.^[^ ^2^ ^]^ Its rapid, isothermal amplification process, along with its compatibility with diverse detection technologies such as lateral flow strips and real‐time fluorescence signal detection, makes it well‐suited for point‐of‐care diagnostics.^[^ ^2^ ^]^ This combination of RPA and CRISPR‐Cas12a technology has enabled the rapid, user‐friendly, and cost‐efficient clinical testing.

While the CRISPR‐Cas12a system is highly regarded for its diagnostic potential, it is not without limitations in accuracy due to potential mismatches in the target sequence that may not inhibit *trans*‐cleavage.^[^ ^3^ ^]^ To enhance the precision in detecting single‐base mutations, researchers have investigated various factors including the location, quantity, and type of nucleotide substitutions.^[^ ^3^, ^4^ ^]^ Notably, some studies have implemented synthetic mismatches to improve the detection of single‐base mutations.^[^ ^3^, ^5^ ^]^ Further advancements include the introduction of strategies such as the application of 2′‐O‐methyl modifications at the 3′ end of crRNA to increase specificity.^[^ ^6^ ^]^ Additionally, the use of DNA‐RNA chimeric crRNAs and crRNAs with a 7‐mer DNA extension at the 3′ end have demonstrated improved capabilities in distinguishing mutations.^[^ ^7^ ^]^ These efforts to improve the specificity of the CRISPR‐Cas12a system clearly illustrate the inherent shortcomings in its ability to precisely identify mismatches in the target sequence, highlighting the ongoing challenge to optimize its diagnostic accuracy.

Human papillomavirus (HPV) is a prevalent viral infection that affects the skin and mucous membranes. HPV types 16, 18, 31, 33, 35, 39, 45, 51, 52, 56, 58, 59, 66, and 68, classified as high‐risk HPV (HR‐HPV), are strongly associated with various cancers, particularly cervical cancer, posing significant health risks globally.^[^ ^8^ ^]^ Of particular clinical importance are HPV16 and HPV18 genotyping, which guide clinical management strategies.^[^ ^9^ ^]^ Thus, accurate HPV detection and typing are critical for early intervention and preventing the onset of HPV‐associated cancers. For instance, the Cobas HPV test (Roche, Basel, Switzerland) is a well‐established approach widely used for detecting 14 HR‐HPV types. While it specifically identifies HPV16 and HPV18, it also provides a pooled detection result for the other 12 HR‐HPV types, facilitating more precise clinical decisions. However, it still relies on expensive equipment and well‐trained personnel, which are not suitable for point‐of‐care diagnostic applications in resource‐limited settings.^[^ ^10^ ^]^

In this study, we systematically investigated the effect of mismatch profiles (e.g., quantity, location, and type of mismatches) on the CRISPR‐Cas12a reaction and designed a dual‐mode detection strategy by engineering crRNAs capable of performing both “exact match” and “approximate pattern match” queries of the target sequences. By strategically introducing specific mismatches in crRNA design, we can constrain “approximate pattern matching” to ensure that a single crRNA simultaneously detects multiple related sequences, while preventing the detection of unintended sequences. Based on this, we further developed a programmable multiplexed nucleic acid assay for the simultaneous detection of 14 HR‐HPV subtypes, which not only specifically distinguishes HPV16 and HPV18 subtypes but also detects 12 other pooled HR‐HPV subtypes. To develop a simple and affordable point‐of‐care test, we further incorporated the assay into a paper‐based microfluidic chip. Finally, we clinically validated the paper‐based microfluidic chip for the simultaneous detection of 14 HR‐HPV subtypes using 75 clinical swab samples.

## 2 Results and Discussion

### 2.1 Investigating Mismatch Profiles in CRISPR‐Cas12a Target Recognition

**Figure 1:**
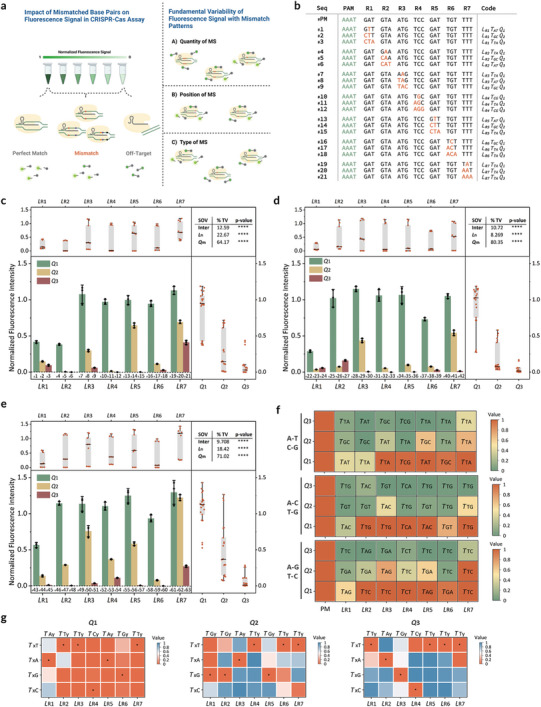
Variability of the fluorescence signal with mismatch profiles. a) Fluorescence signal variation in the CRISPR‐Cas12a assay system influenced by mismatched base pairs. The fluorescence intensity is affected by the quantity, location, and type of mismatches present. MS: mismatch site. Created in BioRender. Guan, X. (2024), https://BioRender.com/j54d267. b) Illustration of mismatch profiles within target‐stranded sequences of activators (PM, 1–21) following the substitution rule A‐T/C‐G. The sequences shown are from HPV16 and were randomly selected for illustration purposes. Each mismatched position is highlighted in red, and the PAM sequence is highlighted in green. PM: a perfect match. c–e) Normalized endpoint fluorescence signals for three types of nucleotide substitutions: c) A‐T/C‐G, d) A‐C/T‐G, and e) A‐G/T‐C, with varying locations and quantities of mismatch sites. For each mismatch type, data are grouped by location (*L_n_*) and mismatch quantity (*Q_m_*) and presented as box plots. Box plots depict the median (middle line), interquartile range (box), and lower and upper adjacent values (whiskers), with outliers represented as individual points. The upper‐right region shows two‐way ANOVA results. Data represent means ± s.d. (n = 3). SOV: source of variation; Inter: interaction; % TV: percentage of total variation. ^****^ *p*‐value < 0.0001. f) Heatmap of the normalized fluorescence signal, annotated with the type of base substitution (*T_xy_*), corresponding to panels (c–e). g) Percentage of fluorescence signal decay for the activator with mismatches. The asterisk (^*^) indicates the crRNA‐complementary nucleotide. The percentage of fluorescence signal decay = (M_FS_ – S_FS_)/ M_FS_, where S_FS_ is the fluorescence signal for the crRNA‐complementary nucleotide and M_FS_ is the fluorescence signal for the mutated activator.

To assess the specificity of the CRISPR‐Cas12a system in response to double‐stranded DNA (dsDNA), we analyzed the *trans*‐cleavage activity of a crRNA‐complementary activator against activators with engineered mismatches (**Figure** **1**a). To investigate the impact of mismatch profiles in the activators, we implemented an encoding strategy for each activator's target‐strand sequence, represented as *L_n_T_xy_Q_m_*. Here, *L_n_* is the numeric location identifier of the substitution within the sequence, *T_xy_* denotes the type of substitution where *x* is the original nucleotide and *y* is the mutated nucleotide, and *Q_m_* indicates the quantity of substitutions. To streamline the mismatch profile analysis, the 21‐bp target region was divided into seven 3‐bp segments (*L*_*R*1_ to *L*_*R*7_). In each segment, 1 to 3 mismatches were intentionally introduced based on three nucleotide substitution rules: transversions (A↔T, C↔G) or (A↔C, T↔G) and transitions (A↔G, T↔C). These modifications were systematically encoded using *T_xy_Q_m_*, which specifies the type and quantity of mismatches introduced.

As a result, increasing *Q_m_*, which indicates a higher number of mismatch sites, led to a pronounced decrease in fluorescence signals (Figure 1c–f). Additionally, we observed that mismatches closer to the PAM, represented by a lower *L_n_*, significantly reduced fluorescence signals, supporting findings from previous research.^[^ ^3a^ ^]^ This phenomenon has also been observed in CRISPR‐mediated defense systems, where the seed region plays a crucial role in recognizing and specifically binding to the corresponding nucleic acid target by adopting a conformation that enhances its ability to search for and interact with target sequences.^[^ ^11^ ^]^ By conducting two‐way analysis of variance (ANOVA) tests for the three nucleotide substitution types, we confirmed that the variables *L_n_* and *Q_m_* significantly influence fluorescence intensity, as illustrated in the top right of Figure 1c–e. Notably, the interaction between *L_n_* and *Q_m_* also significantly impacted fluorescence signal production, revealing a complex interaction of factors affecting the system's output. Simultaneously, grouping the data by *Q_m_*, we noticed that within the PAM‐proximal region, C substitution (*T_xC_*) tended to have a detrimental effect on *trans*‐cleavage, whereas Cas12a‐crRNA exhibited greater tolerance for T substitution (*T_xT_*) (Figure 1g, Figure S1, Supporting Information), consistent with prior research.^[^ ^3a^ ^]^ We further validated these observations by testing a different and randomly chosen sequence (Figure S2, Supporting Information). Our study findings demonstrate that the quantity (*Q_m_*), location (*L_n_*), and type of base substitutions (*T_xy_*) not only independently influence fluorescence signal generation but also interact in a complex manner, underscoring the need for careful consideration when developing CRISPR‐based detection assays.

While the tendency in the fluorescence signal alterations due to mismatches can be observed, accurately predicting the tendencies of variations in fluorescence signal under different mismatch conditions remains challenging. All these specific variations in fluorescence signals with different mismatch profiles may be associated with intricate, interrelated factors such as enzyme turnover rates and enzyme‐substrate affinities.^[^ ^4b^ ^]^ However, our study strategically focuses on three readily adjustable factors to establish a specific mismatch profile. This constrained approach significantly improves our control over the target recognition capabilities of CRISPR‐Cas12a, enabling more precise manipulation and optimization of its diagnostic utility. In future work, we further investigate and explore additional factors that could potentially improve CRISPR‐Cas12a detection, including non‐Watson‐Crick base pairings,^[^ ^12^ ^]^ various spacing between existing mismatches and synthetic ones,^[^ ^5b^ ^]^ and the crRNA length.^[^ ^7^, ^13^ ^]^

In conclusion, by leveraging the inherent specificity limitations of CRISPR‐Cas12a, we demonstrate the effectiveness of approximate pattern matching^[^ ^14^ ^]^ in targeting groups of similar sequences. Concurrently, by fine‐tuning the mismatch profile defined by *L_n_T_xy_Q_m_*, we establish precise query constraints that exclude unintended targets, much like parameters in a database query. This approach enables a programmable and highly controlled multiplexed nucleic acid detection system.

### 2.2 Universal RPA Primer Pair Unlocks Amplification for 14 HR‐HPV Subtypes

**Figure 2:**
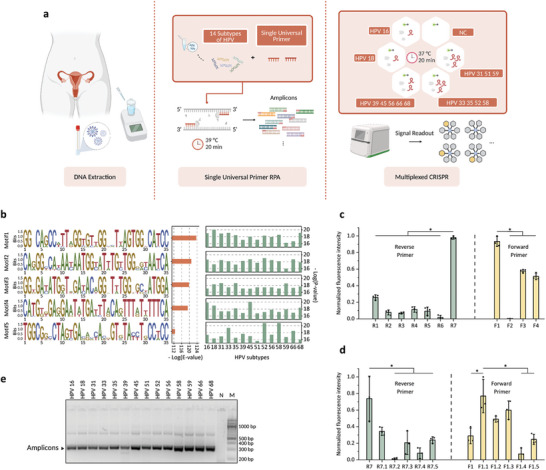
Universal primer pair enables amplification for 14 HPV subtypes. a) Diagrammatic workflow of HPV infection screening and typing. Initially, we extracted DNA from clinical cervical swab samples using a DNA extraction kit. Subsequently, we introduce a universal primer pair with an RPA mixture into the sample, initiating the amplification reaction, which occurs at a constant temperature of 39 °C for 20 min. We then transfer the reaction mixture to the CRISPR reaction chip, which comprises six reaction wells, corresponding to the 14 HPV subtypes and a blank control. An imager captures an image, providing comprehensive results for high‐risk genotypes and individual results for HPV16 and HPV18. NC: negative control. Created in BioRender. Guan, X. (2024), https://BioRender.com/t91o648. b) Sequence logo and detailed information for five motifs. Expectation values (E‐values) represent the expected number of times a particular result or score would occur by chance in a given database or sequence search due to random background noise. The figure shows ‐log(E‐value). The P‐value for each sequence represents the probability of the sequence within each HPV subtype containing the motif in a given sequence dataset, assuming that the appearance of this motif is purely determined by random chance. The figure displays ‐log(P‐value). c,d) Positional and length adjustment of the primer pairs targeting the HPV16 plasmid, with a constant concentration of 50 fM. Motif 1 served as forward primer (F1) and Motif 3 (R4) served as reverse primer. The green bars represent reverse primer adjustments, whereas the yellow bars denote forward primer adjustments. The increase in fluorescence signal during the reaction time, defined as the duration until signal saturation (within 60 min), was recorded and normalized via min‐max scaling. Data represent means ± s.d. (n = 3), with unpaired two‐tailed t‐tests performed. ^*^ *p*‐value < 0.05. e) Agarose gel electrophoresis of RPA products for 14 HPV subtypes using a universal primer pair (299‐bp amplicon). M: marker; N: negative control.

After investigating the mismatch profiles of the CRISPR‐Cas12a reaction system, we explored its application potential by simultaneously detecting 14 HR‐HPV subtypes, which are responsible for 99% of cervical cancer cases,^[^ ^15^ ^]^ as target models. By engineering crRNAs, we developed a programmable multiplexed HPV DNA detection platform by combining RPA amplification with multiple CRISPR detection (**Figure** **2**a). To reduce the testing cost, we sought to use a single universal RPA primer pair, instead of multiple primer pairs, to simultaneously amplify the 14 HR‐HPV subtypes. This strategy also avoids interference caused by a highly complex primer pool, such as primer dimer formation.^[^ ^16^ ^]^ To identify the most conserved sequences across the 14 subtypes of HPV, we conducted bioinformatic analyses of DNA motifs on the highly conserved L1 region.^[^ ^17^ ^]^ Utilizing the bioinformatics software MEME,^[^ ^18^ ^]^ we successfully identified sequences with specific patterns suitable for use as RPA primers. These sequences were ranked based on their E‐values, with lower values indicating higher statistical significance (Figure 2b). We selected the top five motifs as potential primer candidates and aligned them with the HPV genome to obtain detailed information regarding their positions and sequence consistency (Figures S3–S5, Supporting Information). To confirm the conservation of the identified motifs, we aligned them with the most used PCR primers for multiplex HPV detection, namely GP5+/GP6+ and MY09/MY11,^[^ ^19^ ^]^ which are too short to use in the RPA reaction. This alignment revealed partial overlap with the motifs we identified (Figure S3, Supporting Information), suggesting that the motifs we found may possess the capability to detect multiple HPV subtypes. Next, considering the length requirements of RPA amplicons, we selected pairs that resulted in optimal amplicon lengths. The combination of the first and third motifs yielded an amplicon of 299 bp while pairing the second and fourth motifs produced an amplicon of 182 bp. Gel electrophoresis results demonstrated that the first and third motif pairs effectively amplified all HPV subtypes (Figure S6, Supporting Information). However, the pair composed of the second and fourth motifs failed to amplify certain subtypes, such as HPV18 and HPV31 (Figure S7, Supporting Information). Consequently, we identified a promising universal primer pair by incorporating degenerate bases, enabling the simultaneous amplification of all 14 HR‐HPV subtypes.

To further enhance the performance of the RPA amplification, we implemented a multi‐phase primer optimization strategy (Figure S5, Supporting Information). To accurately recognize and detect HPV16, the highest‐risk HPV subtype, we prioritized enhancing its amplification efficiency by RPA reaction. First, we conducted a one‐base positional optimization of the motif primer pair to enhance HPV16 detection via the CRISPR‐Cas12a system, maximizing the primer's ability to bind to the intended target region (Figure 2c). We then methodically adjusted the primer's 3′‐end length by one base to fine‐tune its melting temperature and binding affinity, enhancing the assay's accuracy and yield (Figure 2d). Ultimately, three primer pairs (F1.1‐R7, F1.2‐R7, and F1.3‐R7) exhibited remarkable potential for highly efficient amplification of HPV16 DNA, as evidenced by fluorescence signals (Figure 2d). We subsequently assessed the amplification capacity of these three universal candidate primer pairs for all 14 HPV subtypes through agarose gel electrophoresis (Figure 2e, Figure S8, Supporting Information). The results conclusively demonstrated the successful amplification of all 14 HPV subtypes using the F1.3‐R7 primer pair.

In this step, we improved the overall efficiency of the universal RPA primer pair for detecting HPV16, a crucial subtype in HPV infection, while maintaining the capability to successfully amplify other HPV subtypes. This advancement overcomes the limitations of traditional molecular diagnostics.

### 2.3 Development of a Dual‐Mode Screening Assay for High‐Risk HPV

**Figure 3:**
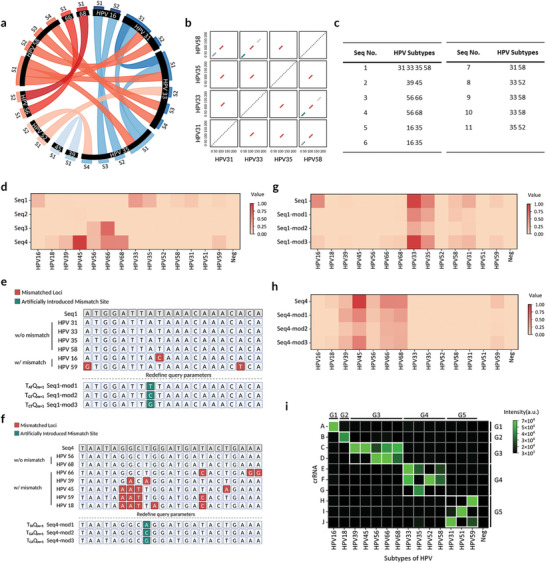
Design and optimization of crRNA combinations. a) Chord diagram, displaying results for pairwise sequence alignment, where connected nodes represent identical sequences. Nodes are labeled with the HPV subtypes to which the sequences belong. For cases in which multiple sequences belong to the same HPV subtype, the nodes are distinguished by labels S1, S2, S3, or S4. b) Results for a subset from the comprehensive all‐against‐all sequence comparison performed using the EMBOSS polydot tool. Matched sequences are differentiated by various colors to highlight distinct alignments. Created in BioRender. Guan, X. (2024), https://BioRender.com/r56p906. c) Table summarizing matched sequences labeled as 1–11 with corresponding HPV subtypes. d) Heatmap displaying normalized detection results for the 14 HR‐HPV subtypes using specifically designed crRNAs targeting sequences Seq1 through Seq4. e) Alignment of Seq1 with HPV subtypes that match perfectly (HPV31, 33, 35, 58) and those that have mismatches (HPV16, 59). The modified crRNAs, Seq1‐mod1 through Seq1‐mod3, utilize distinct strategic approaches. Created in BioRender. Guan, X. (2024), https://BioRender.com/h60m662. f) Alignment of Seq4 with HPV subtypes that match perfectly (HPV56, 68) and those that have mismatches (HPV66, 39, 45, 59, 18). The modified crRNAs, Seq4‐mod1 through Seq4‐mod3, utilize distinct strategic approaches. Created in BioRender. Guan, X. (2024), https://BioRender.com/y83r838. g,h) Heatmap presenting normalized detection outcomes for the 14 HR‐HPV subtypes using modified crRNAs targeting Seq1 and Seq4, respectively. i) Heatmap illustrating the final fluorescence signals generated during the detection of 14 HR‐HPV subtypes using five groups of crRNAs denoted as G1, G2, G3, G4, and G5. The PAM/suboptimal PAM sequences of each target are shown in Table S1 (Supporting Information).

To specifically differentiate HPV16 and HPV18 subtypes from the other 12 HR‐HPV subtypes, we designed a dual‐mode HPV detection strategy, including approximate matching and precise querying of target sequences (Figure 2a). With the dual‐mode detection strategy, we can not only precisely identify HPV16 and HPV18 but also detect other pooled HR‐HPV subtypes. To this end, first, we carefully selected crRNA A and crRNA B, which demonstrated high specificity for HPV16 and HPV18, respectively, without cross‐reactivity with other subtypes. Then, we employed the EMBOSS polydot bioinformatics tool^[^ ^20^ ^]^ for approximate pattern matching, conducting pairwise comparisons of amplicon sequences from 14 HPV subtypes to identify candidate patterns, including those shared by HPV16 and HPV18 (**Figure** **3**a,b, Figure S9, Supporting Information). We also collated common subsequences shared among the HPV subtypes in Figure 3c for enhanced clarity.

Recognizing the diversity and potential of suboptimal PAM sequences, which may perform similarly to the canonical TTTV PAM,^[^ ^21^ ^]^ we initially disregarded the presence of canonical PAM sequences to directly evaluate their effectiveness as approximate matching patterns. To prevent redundant detection of HPV16 and HPV18, and to maximize coverage of the remaining 12 HR‐HPV subtypes, we selected four pattern candidates, Seq1 through Seq4. We aligned these four patterns with all subtypes of HR‐HPV and sequenced them based on their consistency (Figure S10, Supporting Information). As a result, crRNAs targeting Seq1 and Seq4 were not only effective in querying for pattern‐matched HPV subtypes but also unexpectedly matched additional subtypes, as illustrated in Figure 3d. We excluded Seq2 and Seq3 due to ineffective signal generation and target overlap, respectively. Ultimately, after screening, we identified two promising patterns for targeting with the CRISPR‐Cas12a system in the detection of multiple HPV subtypes. Nevertheless, additional constraints are required for these two patterns to ensure the complete exclusion of HPV16 and HPV18 in subsequent detections (Figure 3d).

To improve target specificity via engineered mismatch profiles, we incorporated. *L_n_T_xy_Q_m_* as query constraints for sequence matching. First, we adopted the *Q*_*m* + 1_ strategy with variations in *T_xy_* for both Seq1 and Seq4 (Figure 3e,f). This approach aimed to create regions of continuous mismatches to refine the specificity of target detection. For Seq1, which initially had only a single nucleotide difference from the HPV16 sequence, we introduced an additional mismatch at a nearby site, varying the nucleotide substitution types. This modification, resulting in Seq1‐mod1, successfully prevented cross‐reactivity with HPV16 while allowing precise detection of HPV33, HPV35, and HPV58, although the detection of HPV31 yielded an insufficient fluorescence signal (Figure 3g). This sequence was designated as crRNA E, the core crRNA for one detection group.

In the case of Seq4, despite the presence of a scattered mismatch pattern, we focused on the three fundamental factors outlined above to regulate the specificity of the CRISPR‐Cas12a system. Similarly, we introduced intentional mismatches, creating a 5‐base mismatch region between the crRNA and the HPV18 sequence. This modification aimed to reduce the fluorescence signal when detecting HPV18. Moreover, in the case of Seq4, in addition to applying *T_xy_Q_m_* adjustments, we further explored specificity enhancements by modifying *L_n_*, reducing the *n* value to shift the mismatch closer to the 5′ end of the crRNA. The resulting sequences, Seq4‐mod4, Seq4‐mod5, and Seq4‐mod6, all showed significantly decreased fluorescence signals for mismatched subtypes, demonstrating the effectiveness of this approach (Figure S11, Supporting Information). Among these, Seq4‐mod1 successfully excluded HPV18 while maintaining recognition of multiple other subtypes (Figure 3h), leading to its designation as crRNA C, the core of another detection group. Through these strategic approaches within the *L_n_T_xy_Q_m_* framework, we established query constraints within the approximate pattern‐matching process to restrict the targets detectable by the CRISPR‐Cas12a system.

To address signal loss or weakness in detecting certain HPV subtypes, such as HPV56 and HPV58, we designed supplementary crRNAs located near the target regions of the core crRNAs in each group, but with greater sequence consistency for the subtypes needing enhancement. This strategy leverages the high sequence conservation in specific motifs within Seq1 and Seq4, ensuring that the new crRNAs align with the existing ones in the detection range while improving efficiency. This approach was applied to crRNA D in Group 3 (Figure S12, Supporting Information) and crRNA F in Group 4 (Figure S13, Supporting Information). For the remaining subtypes that could not be detected (e.g., HPV 31, 35, 51, and 59), we introduced subtype‐specific supplementary crRNAs (crRNA G‐J) without considering sequence conservation. Additionally, to minimize interference and cross‐reactivity, we strategically grouped crRNAs by the detected HPV subtypes, reducing the number of crRNAs in each group.

Ultimately, we successfully developed an assay with five detection systems, each corresponding to a specific group of crRNAs, enabling the simultaneous detection of 14 HR‐HPV subtypes. The first and second systems each contain a single crRNA targeting HPV16 and HPV18, respectively. The third system includes two crRNAs for detecting HPV 39, 45, 56, 66, and 68. The fourth and fifth systems each contain three crRNAs, targeting HPV 33, 35, 52, 58, and HPV 31, 51, and 59, respectively. This design allows for individual detection of HPV16 and HPV18, while the remaining 12 subtypes are collectively assessed across the other three groups (Figure 3i; Figure S14, Supporting Information).

### 2.4 Optimizing the CRISPR‐Cas12a Detection System

**Figure 4:**
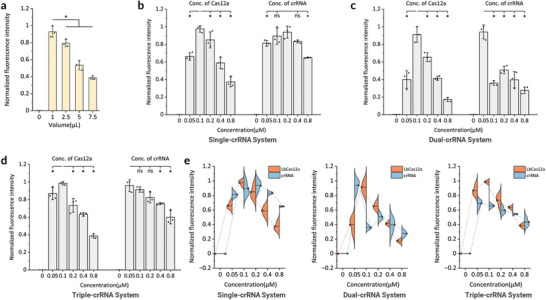
Optimization of the RPA/CRISPR‐Cas12a reaction system. a) Adjusting the RPA product volume within the CRISPR‐Cas12a reaction system. Different volumes (e.g., 1, 2.5, 5, and 7.5 µL) of RPA product of HPV16 at 20 fM were introduced into the CRISPR reaction solution with a final reaction volume of 10 µL. (b‐d) Normalized fluorescence signal for optimizing LbCas12a and crRNA concentrations in (b) single‐, c) dual‐, and (d) triple‐crRNA CRISPR reactions. We utilized plasmids of HPV16, HPV39, and HPV31 at a concentration of 20 fM to optimize the three reaction systems. Data represent means ± s.d. (n = 3), with unpaired two‐tailed t‐tests performed. ^*^ *p*‐value < 0.05; ns: not significant. e) A comprehensive summary of LbCas12a and crRNA concentrations in the three CRISPR detection systems (single‐, dual‐, and triple‐crRNA). Violin plots compare data distributions, with red for LbCas12a and blue for crRNA. Dotted lines show averages, and kernel smoothing enhances pattern visibility.

Next, we optimized the CRISPR‐Cas12a systems by adjusting the concentrations of crRNA(s) and LbCas12a in single‐, dual‐, and triple‐crRNA detection setups. Initially, we optimized the volume of RPA products added to the CRISPR reaction (**Figure** **4**a). Consistent with previous findings,^[^ ^22^ ^]^ RPA mixtures were found to interfere with the CRISPR reaction. Increasing the amount of RPA mixture added to the CRISPR reaction resulted in a reduction in *trans*‐cleavage activity (Figure 4a). Eventually, we determined that adding 1 µL of RPA product effectively activated the CRISPR‐Cas12a reaction. In the single‐crRNA reaction system, Cas12a produced relatively higher fluorescence signals when the ratio of Cas12a to crRNA was 1:2 (Figure 4b). However, in the presence of multiple crRNAs, with consistent enzyme concentrations, the optimal concentration of individual crRNAs tended to decrease, due to potential competition for enzyme binding and activity (Figure 4c,d).^[^ ^23^ ^]^ Consequently, we established the final concentration of LbCas12a at 0.1 µM for all three reaction systems, with each crRNA concentration set at 0.2 µm for single‐crRNA systems (for the detection of HPV16 and HPV18), and 0.05 µm for both dual‐crRNA (for the detection of HPV39, HPV45, HPV56, HPV66, and HPV68) and triple‐crRNA (for the detection of HPV33, HPV35, HPV52, HPV58, HPV31, HPV51, and HPV59) systems. Overall, we developed and optimized a programmable multiplexed DNA assay for simultaneous detection of 14 HR‐HPV subtypes.

### 2.5 Assessing the Detection Sensitivity for 14 HR‐HPV Subtypes

**Figure 5:**
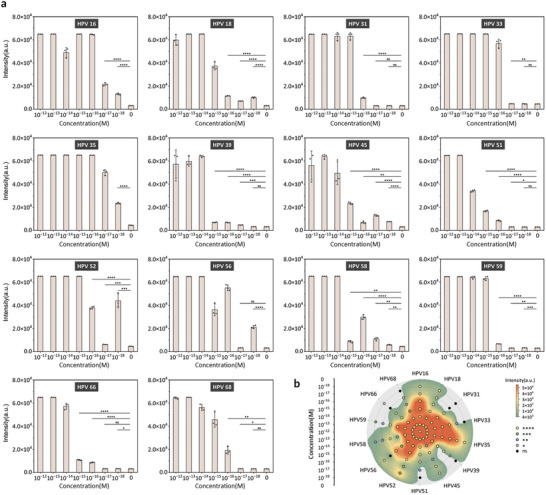
Sensitivity assessment of the programmable multiplexed DNA assay for 14 HR‐HPV subtypes. a) Sensitivity testing results for 14 HR‐HPV subtypes, including HPV16, 18, 31, 33, 35, 39, 45, 51, 52, 56, 58, 59, 66, and 68. The experiments involved CRISPR‐Cas12a detection following RPA amplification of HPV DNA plasmids with varying concentrations for each HPV subtype. Data represent means ± s.d. (n = 3). Statistical significance was determined using unpaired two‐tailed t‐tests. ^****^ *p*‐value < 0.0001, ^***^ *p*‐value < 0.001, ^**^ *p*‐value < 0.01, ^*^ *p*‐value < 0.05, ns: not significant. b) Polar contour plot depicting sensitivity results for all subtypes. The coordinate axes indicate concentration levels represented by each ring, while radial lines represent different HPV subtypes. Colors signify varying fluorescence signal intensities. The colored dots on the plot represent the p‐value of the fluorescence signal for each subtype at the corresponding concentration, relative to the negative control. The plot has undergone smoothing for visualization.

Through the optimized experimental parameters outlined above, we conducted sensitivity assessments for 14 HR‐HPV subtypes. HPV DNA plasmid samples spanning a concentration gradient from 10^−12^ to 10^−18^ M were subjected to testing, with 0 m serving as the negative control. Each sample underwent initial RPA amplification using the single universal primer pair, followed by fluorescence signal detection utilizing the optimized CRISPR‐Cas12a *trans*‐cleavage assay (**Figure** **5**a). In CRISPR‐Cas12a detection systems utilizing two or three crRNAs, we conducted tests using a combination of crRNAs instead of the specific crRNA designed for each HPV subtype. Figure 5b and Figure S15 (Supporting Information) provide a comprehensive overview of the sensitivity evaluations conducted across all 14 HR‐HPV subtypes. The dual‐ or triple‐crRNA detection systems maintained high detection efficacy compared to the single‐crRNA system and notably excelled in detecting specific subtypes, such as HPV35. However, the fluorescence signal for certain subtypes, such as HPV39, exhibited decreased intensity, potentially attributed to constraints in RPA amplification. Gel electrophoresis results of RPA products corroborated this observation (Figure 2e). Nevertheless, significant differences in endpoint signals persisted between the samples and negative controls, even at an initial HPV DNA plasmid concentration of 10^−17^ m. Overall, the assay demonstrated remarkable detection sensitivities ranging from 1 to 100 attomolar across all HR‐HPV subtypes, underscoring its robust performance despite sensitivity variations among different subtypes. In summary, our programmable multiplexed nucleic acid assay exhibits considerable potential for highly sensitive simultaneous detection of multiple targets.

### 2.6 Clinical Validation of the Programmable Multiplexed DNA Detection System on a Paper‐Based Microfluidic Chip

**Figure 6:**
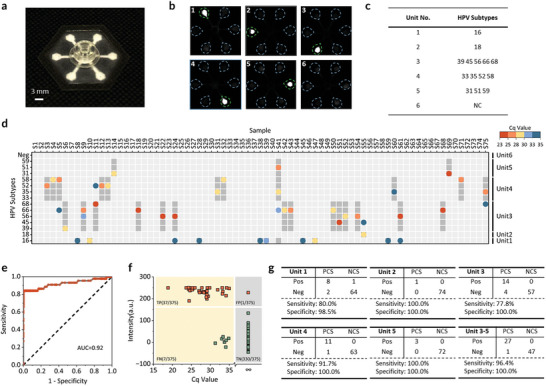
Clinical validation of the programmable multiplexed DNA assay on a paper‐based microfluidic chip. a) Photograph of the paper‐based microfluidic chip, which consists of i) a paper substrate hosting microfluidic channels formed by wax, ii) two layers of sealing film, and iii) a 3D‐printed RPA sample‐loading chamber, enclosed with an additional layer of sealing tape (Figure S16, Supporting Information). b) Images display fluorescence for positive results in units 1–5, shown in images 1–5. Image 6 shows a fluorescence image for the negative control (NC). Images 1–6 correspond to samples S8, S55, S54, S32, S69, and S73, respectively. c) HPV subtypes are identified within different detection units. d) Results of a blind test involving 75 clinical samples, utilizing both PCR and the microfluidic chip. Dark gray boxes indicate the chip's positive detection units, whereas circles represent PCR outcomes for HPV subtypes with Cq values ≤ 35, with colors reflecting their respective Cq values (Table S2, Supporting Information). e) ROC plot for the detection method. The area under the curve (AUC) of the ROC plot is calculated as 0.92. The black dashed line represents a random classifier. f) Scatter plot depicting background‐subtracted fluorescence signal values against corresponding Cq values. For cases in which multiple subtypes were detected by PCR within one detection unit, the minimum Cq value was selected. Here, we set a Cq threshold of 35 and an intensity threshold of 162 in arbitrary units. Each square represents an individual sample. Red squares indicate samples identified as positive by the chip, while green squares indicate negative results. Squares on a yellow background correspond to PCR‐positive samples, whereas squares on a gray background correspond to PCR‐negative samples (Table S4, Supporting Information). TP: true positive; FP: false positive; FN: false negative; TN: true negative. g) Statistical results for samples in units 1–5 and combined results for units 3–5, presenting sensitivity and specificity for each detection unit. PCS: positive clinical sample; NCS: negative clinical sample; Pos: samples tested positive by the chip; Neg: samples tested negative by the chip.

To assess the assay's applicability in clinical settings, we developed a paper‐based microfluidic chip to enable simple, rapid, multiplexed detection of 14 HR‐HPV subtypes at the point of care (**Figure** **6**a; Figure S16, Supporting Information). This chip features six distinct detection zones, with units 1–5 aligned with groups 1–5 in the CRISPR‐Cas12a assay, in addition to a blank control (Figure 6b,c). Lyophilized CRISPR enzyme/specific crRNAs for HPV subtype tests are pre‐stored in the individual units of the chip as detailed in Figure 6c. Corresponding fluorescence signals from individual reaction units are depicted in Figure 6b, with each unit labeled accordingly. Furthermore, we assessed the sensitivity of our paper‐based microfluidic chip. We selected representative subtypes from each reaction unit and achieved the detection sensitivities of 100 attomolar or less (Figure S17, Supporting Information), similar to that of the tube‐based reactions.

De‐identified clinical cervical swab samples were provided by the Clinical Microbiology Laboratory with a protocol approved by the ethics committee at the University of Connecticut Health Center (IRB# 22–215). During clinical sample testing, we first extracted DNA from 75 clinical swab samples. To ensure accurate detection and differentiation of HPV subtypes, we designed qPCR primers and probes targeting the 14 HR‐HPV subtypes and conducted tests to confirm no cross‐reactivity. Additionally, to ensure the quality of the extracted DNA, qPCR was employed to detect the reference gene GAPDH (Table S2, Supporting Information). Then, we utilized the RPA assay with a single universal primer pair to amplify the extracted DNA. After a 20‐min RPA incubation, we introduced the RPA products into our paper‐based microfluidic chip for CRISPR‐Cas12a detection. After incubating the chip at 37 °C for 20 min, the final fluorescence image of the platform was captured (Figure S18, Supporting Information). The summarized results, depicted in Figure 6d, integrate the qPCR detection outcomes, denoted by colored circles representing varying Cq values, and fluorescence signals observed in the corresponding chip detection units, highlighted by dark gray boxes. Overall, most of the clinical samples were accurately identified and typed. In instances where only a single HPV subtype was detected in the qPCR results, our chip yielded consistent outcomes. However, in cases such as S5, S11, and S24, where multiple HPV subtypes coexisted, our chip often prioritized indicating the subtype with a lower Cq value, which signifies a higher viral load. This prioritization could potentially overlook subtypes with lower viral loads, typically within the Cq value range of 33–35 (Table S3, Supporting Information). Moreover, despite our assay identifying S41 as having an HPV16 infection, the qPCR results indicated HPV16 negativity, suggesting a false positive result possibly attributed to sample handling issues.

To further assess the effect of increased incubation time on the detection specificity of our system, we tested seven clinical samples, including one negative and six positive samples, on our paper‐based microfluidic chip (Figure S19, Supporting Information). Fluorescence images were captured after 20, 40, and 60 min of incubation, as well as following overnight reaction at room temperature. The results showed a gradual increase in fluorescence signal intensity over time for positive samples, without false positive signals observed.

To comprehensively evaluate the chip's performance, we conducted statistical analyses on the qPCR detection results and the numerical fluorescence signals processed using Image J. By utilizing the Cq values and the increased fluorescence signal values of each detection unit relative to the blank control, we identified each detection unit of every sample (Table S4, Supporting Information). The receiver operating characteristic (ROC) curve, with an area under the curve (AUC) of 0.92, demonstrated excellent performance of the platform.^[^ ^24^ ^]^ Using the Youden Index, we determined the optimal cut‐off value for the relative increased fluorescence signal, set at 162. Subsequently, using this cut‐off value, a scatter plot (Figure 6f) categorized the detection units into four distinct parts for clarity. Calculated sensitivities, specificities, positive predictive values, and negative predictive values were 84.1, 99.7, 97.4, and 97.9%, respectively. Additionally, we performed a statistical analysis for each detection unit. The sensitivities of the detection units 1–5 were 80.0, 100.0, 77.8, 91.7, and 100.0%, respectively, and the specificities were 98.5%, 100.0%, 100.0%, 100.0%, and 100.0%, respectively. Then, we combined the detection results of units 3–5 for reporting all 12 subtypes collectively, resulting in a sensitivity and specificity of 96.4 and 100.0%, respectively (Figure 6g). Furthermore, we classified the samples according to the number of HPV infection types. Among the 75 samples, 39 were uninfected, 28 had a single infection, while 8 samples showed simultaneous infections with two or more subtypes. It is noteworthy that our chip achieved a 100% consistency rate in reporting results for negative samples and those infected with only one subtype of HPV. However, for samples infected with multiple HPV subtypes and exhibiting significant concentration differences among different subtypes, the chip accurately reported the higher concentrations of one or more subtypes.

In summary, our paper‐based microfluidic chip demonstrates a promising diagnostic alternative for multiplexed nucleic acid detection, offering a simple and affordable diagnostic tool at the point of care.

## 3 Conclusion

CRISPR‐Cas12a has recently gained attention as an effective tool for nucleic acid detection, although it sometimes struggles to differentiate targets with mismatches due to its inherent specificity limitations. In this study, we capitalized on the specificity defect of CRISPR‐Cas12a to develop a programmable multiplexed nucleic acid detection system. Specifically, we analyzed the mismatch profile (*L_n_T_xy_Q_m_*) to alter the target recognition capabilities of CRISPR‐Cas12a. To enhance the discrimination ability of CRISPR‐Cas12a systems, we found that increasing the number of mismatches (*Q_m_*) or positioning mismatches closer to the PAM‐proximal region (*L_n_*) could facilitate the recognition of a single mismatch. Conversely, to conduct an approximate matching across a group of sequences, targeting the subsequence with the highest consistency and moving the mismatch position toward the PAM‐distal region could be effective. Additionally, changing the type of mismatch (*T_xy_*) may also alter the *trans*‐cleavage activity.

By employing the modulation strategies outlined above, we developed a programmable dual‐mode nucleic acid assay that enables the individual detection of HPV16 and HPV18, alongside pooled detection of 12 other HR‐HPV subtypes. While this design efficiently identifies the broader group of HR‐HPV subtypes, additional detection methods (e.g., PCR) may be required in clinical settings where detailed subtype identification is necessary. Additionally, we integrated RPA technology with a pair of universal primers, significantly simplifying the assay. What's more, to facilitate simple and cost‐effective point‐of‐care testing, we embedded the multiplexed assay into a paper‐based microfluidic chip. We further tested the chip with 75 clinical swab samples and validated its clinical feasibility.

To further validate our constrained strategy, we applied it to detect the hepatitis C virus (HCV),^[^ ^25^ ^]^ focusing on its six major genotypes (Figure S20, Supporting Information). By leveraging mismatch profiles, we developed an assay that accurately identifies HCV genotype 1 while providing pooled detection for genotypes 2, 3, 4, 5, and 6, without cross‐reactivity with genotype 1. This approach enhances clinical information and conserves resources by guiding precise detection of HCV genotype 1, which is crucial for effective clinical treatment.^[^ ^25^ ^]^ In conclusion, our study successfully balances multiplex detection with individual subtype detection while addressing the challenge of mitigating positive signals from unintended targets with similar sequence patterns during pooled detection using the CRISPR‐Cas12a system.

In previous studies focusing on HPV multiplex detection, researchers typically utilized multiplexed RPA to amplify several types of HPV using primer pools.^[^ ^16^, ^26^ ^]^ When multiple primer pairs are present in the reaction simultaneously, the amplification system requires careful testing to ensure the optimization of primer compatibility, concentration ratios, and other factors. To address these potential concerns, we employed a systematic approach in our research to design RPA primers. This approach enabled the amplification of all subtypes using a universal primer pair. Moreover, prior investigations designed specific crRNAs for each subtype due to the precision of CRISPR‐Cas12a specificity.^[^ ^16^, ^26^ ^]^ To achieve this aim, the sharpness of the CRISPR‐Cas12a specificity is necessary. In contrast, based on the widely acknowledged tolerance to mismatches,^[^ ^4^, ^7^, ^27^ ^]^ we observed the potential benefits that this defect brings to multi‐target recognition.

While our approach facilitates the design of crRNA for multiple targets, we acknowledge the necessity for additional methods to improve workflow efficiency, such as predicting the structural compatibility of crRNA with the target sequence. While our detection assay and paper‐based microfluidic chip provide significant advantages, including cost reduction ($2.62 per detection, Table S5, Supporting Information), simplified manipulation, and time savings, integrating the system with automated equipment could further enhance stability and consistency, making it an even more reliable and efficient tool for clinical applications. To further refine our approach, our main objective is to enhance the precision of detecting multiple HR‐HPV infections in samples, ensuring accurate reporting of all HPV subtypes present, despite single HPV infection cases being predominant.^[^ ^28^ ^]^ In the future, we can further optimize primer design and CRISPR reaction conditions to improve the detection efficiency, enabling uniform amplification of different subtypes. Additionally, simplifying DNA extraction by using heat to release HPV DNA could further streamline the detection process.

In conclusion, we leveraged the specificity limitations of CRISPR‐Cas12a to develop a programmable dual‐mode nucleic acid assay for the simultaneous detection of multiple targets. This innovative approach has been integrated into an affordable paper‐based microfluidic chip, rendering it ideal for point‐of‐care applications. By strategically utilizing the specificity constraints of the CRISPR‐Cas12a system, we not only enable precise identification of specific sequences but also facilitate a broader screening for related sequences when necessary, thus enhancing the assay's versatility and expanding its clinical utility. Overall, this approach holds significant promise for simple, sensitive, specific, and multiplexed detection of various pathogens in resource‐limited settings.

## Conflict of Interest

The authors declare no conflict of interest.

## Supporting information



Supporting Information

## Data Availability

The data that support the findings of this study are available in the supplementary material of this article.
