# Supplementary material for: Programmable Multiplexed Nucleic Acid Detection by Harnessing Specificity Defect of CRISPR‐Cas12a
Source: Adv Sci (Weinh). 2024 Dec 4;12(4):2411021. doi: 10.1002/advs.202411021 (PMC11775522; doi:10.1002/advs.202411021)
Supplement: Supplementary file 1 — Supporting Information [file ADVS-12-2411021-s001.pdf]

ADVANCED SCIENCE
Open Access

Supporting Information

for *Adv. Sci.*, DOI 10.1002/advs.202411021

Programmable Multiplexed Nucleic Acid Detection by Harnessing Specificity Defect of CRISPR-Cas12a

*Xin Guan, Rui Yang, Jiongyu Zhang, Jeong Moon, Chengyu Hou, Chong Guo, Lori Avery, Danielle Scarola, Daniel S. Roberts, Rocco LaSala and Changchun Liu**

Supporting Information

Programmable Multiplexed Nucleic Acid Detection by Harnessing Specificity Defect of CRISPR-Cas12a

Xin Guan[1,2], Rui Yang[1,2], Jiongyu Zhang[1,2], Jeong Moon[1], Chengyu Hou[1,2], Chong Guo[1,2], Lori Avery[3], Danielle Scarola [4], Daniel S. Roberts [4], Rocco LaSala[3], and Changchun Liu[1]*

1. *Department of Biomedical Engineering, University of Connecticut Health Center, Farmington, Connecticut 06030, United States*
2. *Department of Biomedical Engineering, University of Connecticut, Storrs, Connecticut 06269, United States*
3. *Department of Pathology and Laboratory Medicine, University of Connecticut Health Center, Farmington, Connecticut 06030, United States*
4. *Division of Otolaryngology - Head & Neck Surgery, University of Connecticut Health Center, Farmington, Connecticut 06030, United States*

*** Corresponding author**

Dr. Changchun Liu

Department of Biomedical Engineering

University of Connecticut Health Center

263 Farmington Avenue

Farmington, CT 06030

Phone: (860)-679-2565

E-mail: chaliu@uchc.edu

1. Methods

Ethical statement

De-identified clinical cervical swab samples were provided by the Clinical Microbiology Laboratory with a protocol approved by the ethics committee at the University of Connecticut Health Center (IRB# 22-215).

Materials

Nuclease-free water was purchased from New England Biolab (NEB). The TwistAmp® Basic kit was purchased from TwistDx™ Limited. All nucleotides were synthesized by Integrated DNA Technologies (IDT). The Alt-R™ L.b. Cas12a (Cpf1) Ultra used in the CRISPR reaction was obtained from IDT. NEBuffer™ 4 was obtained from NEB. For gel electrophoresis, 40% acrylamide / bis solution (19:1), ammonium persulfate (APS), 10×Tris-borate-EDTA (TBE) buffer, tetramethylethylenediamine (TEMED), and Certified™ Molecular Biology Agarose were purchased from Bio-Rad Laboratories. SYBR™ Gold Nucleic Acid Gel Stain (10,000× Concentrate in DMSO) was purchased from Thermo Fisher Scientific. TriDye™ 100 bp DNA ladder and Gel Loading Dye Purple (6×) were obtained from NEB. The DNeasy Blood and Tissue kit was obtained from QIAGEN. The GoTaq® Probe qPCR kit was purchased from Promega. D-(+)-Trehalose dihydrate was purchased from Sigma-Aldrich. Microseal 'B' Adhesive Sealing Film was obtained from Bio-Rad Laboratories. Clear resin was purchased from Formlabs.

CRISPR-Cas12a *trans*-cleavage assay for mismatched targets

To examine the impact of mismatch sites on fluorescence signals, a 20 µL reaction mixture was prepared, consisting of 1×NEBuffer™ 4, 200 nM of crRNA, 100 nM of Alt-R™ L.b. Cas12a, 5 µM of ssDNA-FQ(/56-FAM/TTATT/3IABkFQ/), and 2 µL of the activator. The concentration of dsDNA was maintained at 5 nM. Following thorough mixing, the mixture was incubated at 37°C in the CFX96 Touch Real-Time PCR Detection System (Bio-Rad Laboratories) for 30 min, with

fluorescence detection recorded per minute. The positive control utilized a perfect-match DNA activator, while the negative control lacked any activator. The normalized data were processed using this scaling, defined as:

$$x_{norm} = \frac{x - x_{Neg}}{x_{Pos} - x_{Neg}}$$

Two-way ANOVA tests and Tukey's multiple comparisons tests were performed using GraphPad Prism 10.

Motif search

Motifs within a set of 14 HPV sequences were identified using the bioinformatics tool MEME (Multiple Em for Motif Elicitation) (https://meme-suite.org/meme/tools/meme)[1], which employs an unsupervised learning algorithm. Parameters were set to detect motifs occurring once per sequence with a sequence width from 30–35 bp, aiding in the selection of optimal primers for RPA amplification. The significance of identified motifs was assessed using E-value and P-value metrics, offering quantitative measures of motif significance and indicating the likelihood of observing these motifs by chance alone.

RPA assay

RPA reactions were conducted utilizing the TwistAmp® Basic kit, adhering to the instructions provided in the manual. The reaction mixture comprised 29.5 µL of rehydration buffer, 480 nM of forward and reverse primers, 14 mM of magnesium acetate, and 7.5 µL of either plasmid or extracted DNA. For the negative control, nuclease-free water was used instead of nucleic acid. Subsequently, the reaction proceeded at 39°C for 20 min. The resulting RPA products were then stored at -20°C for further use.

CRISPR-Cas12a *trans*-cleavage assay for primer optimization

The RPA/CRISPR assay protocol was developed according to our previous studies[2]. The RPA reaction was performed in a final volume of 50 μL, comprising 29.5 μL of rehydration buffer, 480 nM of forward and reverse primers, 14 mM of magnesium acetate, and 7.5 μL of plasmid at a concentration of 20 fM. Subsequently, the CRISPR-Cas12a *trans*-cleavage reaction was conducted with 1× NEBuffer™ 4, 200 nM of crRNA, 100 nM of Alt-R™ L.b. Cas12a, 5 μM of ssDNA-FQ(/56-FAM/TTATT/3IABkFQ/), and 1 μL of the RPA product, in a final volume of 10 μL. The reaction was carried out at 37°C in the CFX96 Touch Real-Time PCR Detection System (Bio-Rad Laboratories) for 60 min, with fluorescence recorded every minute. Data normalization was conducted using min-max scaling at each step of primer optimization. The unpaired two-tailed t-tests were performed using Origin 2023b.

Agarose gel electrophoresis

A 3% TBE agarose gel electrophoresis method was employed to visualize the RPA amplicons, with a plasmid concentration of 10 pM. To prepare the gel, 3g of agarose powder was weighed and added to a flask containing 10 mL of 10×TBE buffer and 90 mL of deionized water. After heating the mixture until complete dissolution of agarose, it was cooled to 60–70°C. Subsequently, 10 μL of SYBR gold was added, and the solution was poured into a gel casting tray with a comb inserted to form wells, left to solidify for 20–30 min. RPA products were premixed with gel loading dye and nuclease-free water, with 10 μL of the mixture loaded into each lane of the gel. Electrophoresis was conducted at a constant 100V for 90 min in 1×TBE buffer, followed by gel scanning using the Bio-Rad ChemiDocTM MP imaging system.

Native PAGE

An 8% denaturing polyacrylamide gel electrophoresis (PAGE) method was employed to visualize the RPA amplicons, with a plasmid concentration of 10 nM. Images of the amplicons are provided in the supplementary data. To prepare the gel, 4 mL of 40% acrylamide/bis solution (19:1), 2 mL of 10×TBE buffer, 14 mL of deionized water, 8 μL of TEMED, and 66 μL of 30% ammonium persulfate solution (prepared using 3 mg in 100 μL nuclease-free water) were mixed.

The mixture was then transferred to the gel-casting chamber and allowed to solidify. All RPA amplicons were premixed with gel loading dye and added to each lane of the gel. Electrophoresis was conducted at a constant 100V for 90 min in 1×TBE buffer. Subsequently, the gel was stained with dissolved SYBR™ Gold Nucleic Acid Gel Stain solution for 10 min in a dark environment. Finally, images were obtained using the Bio-Rad ChemiDocTM MP imaging system.

Pairwise sequence alignment

EMBOSS polydot (https://www.ebi.ac.uk/jdispatcher/seqstats/emboss_polydot)[3] constructs dot plots from pairwise comparisons of sequences within a set, portraying them on a unified graph. Dot plots visually highlight similarities between sequences, with each sequence representing an axis. Exact matches between sequence regions prompt dot placement, forming diagonal patterns indicating regions of similarity. For our analysis, we set the word size for comparison to 20–24 base pairs, matching the length of crRNA spacers. Information regarding matching regions for each HPV subtype was saved to a file.

CRISPR-Cas12a *trans*-cleavage assay cross-reactivity test

The CRISPR-Cas12a *trans*-cleavage reaction was conducted with 1× NEBuffer™ 4, 200 nM of crRNA, 100 nM of Alt-R™ L.b. Cas12a, 5 μM of ssDNA-FQ(/56-FAM/TTATT/3IABkFQ/), and 2.5 μL of plasmids at a concentration of 10 nM, in a final volume of 10 μL. The reaction was carried out at 37°C in the CFX96 Touch Real-Time PCR Detection System (Bio-Rad Laboratories) for 60 min, with fluorescence recorded every minute. Data normalization was conducted using min-max scaling for each cross-reactivity test.

System optimization of the CRISPR-Cas12a *trans*-cleavage assay

The RPA reaction was performed in a final volume of 50 μL, comprising 29.5 μL of rehydration buffer, 480 nM of forward and reverse primers, 14 mM of magnesium acetate, and 7.5 μL of plasmid for HPV16, HPV39, or HPV31 at a concentration of 20 fM for single-, dual- or triple-

crRNA detection system optimization, respectfully. Then, the CRISPR-Cas12a *trans*-cleavage reaction was conducted in a final volume of 10 μL with 1× NEBuffer™ 4, 5 μM of ssDNA-FQ(/56-FAM/TTATT/3IABkFQ/), varying concentrations of crRNA and Alt-R™ L.b. Cas12a, activated by different volumes of RPA products. The reaction was carried out at 37°C in the CFX96 Touch Real-Time PCR Detection System (Bio-Rad Laboratories) for 60 min, with fluorescence recorded every minute. Data normalization was conducted using min-max scaling at each step of optimization. The unpaired two-tailed t-tests were performed using Origin 2023b.

Sensitivity test of the 14 subtypes of HPV

The RPA reaction was carried out in a 50 μL volume with rehydration buffer, forward and reverse primers (480 nM each), magnesium acetate (14 mM), and plasmid (7.5 μL) at varying concentrations. Subsequently, the CRISPR-Cas12a *trans*-cleavage reaction was conducted in a final volume of 10 μL with NEBuffer™ 4, Alt-R™ L.b. Cas12a (100 nM), ssDNA-FQ (/56-FAM/TTATT/3IABkFQ/) (5 μM), and 1 μL of RPA product. The crRNA concentrations were 200 nM for single-crRNA and 50 nM for dual- and triple-crRNA systems. The reactions were performed at 37°C in the CFX96 Touch Real-Time PCR Detection System (Bio-Rad Laboratories) for 60 min, with fluorescence readings taken every minute. The unpaired two-tailed t-tests were performed using Origin 2023b.

Clinical sample preparation and qPCR detection

The extraction of HPV DNA from clinical cervical swab samples was performed using the DNeasy Blood and Tissue Kit, following its protocol meticulously. Subsequently, qPCR was conducted according to the GoTaq® Probe qPCR Master Mix protocol. The reaction mixture comprised GoTaq® Probe qPCR Master Mix (1×), forward primer (500 nM), reverse primer (500 nM), hydrolysis probe (250 nM), template DNA, and nuclease-free water. The qPCR reaction proceeded with the following cycling conditions: initial GoTaq® DNA polymerase activation at 95°C for two min; denaturation at 95°C for 15 s, followed by annealing/extension at

60°C for one min, repeated for 40 cycles. The fluorescence signal was captured by the CFX96 Touch Real-Time PCR Detection System (Bio-Rad Laboratories).

Fabrication and testing of the paper-based microfluidic chip

The chip design was created using PowerPoint and printed on Whatman Grade 1 Qualitative Filter Paper using a wax printer (Xerox ColorQube 8870). Subsequently, a hot plate (Thermo Scientific Cimarec Hotplate) was used to heat the paper-based chip at 120°C for 40 s, allowing the wax to penetrate to the other side. After cooling to room temperature, the paper-based chip was placed in a dish, and 2 μL of CRISPR-Cas12a reaction mixture, containing 10% trehalose, was added to each reaction chamber using a pipette. The paper-based chip was then frozen at -80°C for 30 min and subsequently freeze-dried for over two hours in a Labconco FreeZone Freeze Dryer System. After this, both sides of the dried paper-based chip were sealed with PCR sealing film, leaving sample injection holes on the front side. The 3D-printed sample loading chamber, designed using SolidWorks and printed by a Form 3B+ 3D printer, was adhered to the chip using double-sided tape. After mixing 15 μL of RPA product with 5 μL of deionized water, the mixture was added to the sample loading chamber, which was then sealed with sealing tape. The loaded chip was then placed in an Applied Biosystems ProFlex incubator at 37°C for 20 min. Finally, images of the chip were captured using the Bio-Rad ChemiDocTM MP imaging system and analyzed by Image J. The ROC curve along with the related statistical analyses were performed using Origin 2023b. The Youden Index[4] was used to determine the optimal threshold value, which was defined as:

$$J = max_c\{ S_e(c) + S_p(c) - 1\}$$

where S_e represents sensitivity and S_p stands for specificity. The cut-off value corresponding to the maximum J statistic was chosen as the optimal threshold (c^*) for the diagnostic test.

Statistical information

Unless specified otherwise, the unpaired two-tailed t-test, two-way ANOVA test, and ROC analyses were performed using Origin 2023b. Standard deviations and mean values were calculated in Excel (Microsoft Office) based on data from three identical experiments. The normalized data were used in min-max scaling, defined as:

$$x_{norm} = \frac{x - x_{min}}{x_{max} - x_{min}}$$

2. Supporting Figures and Tables

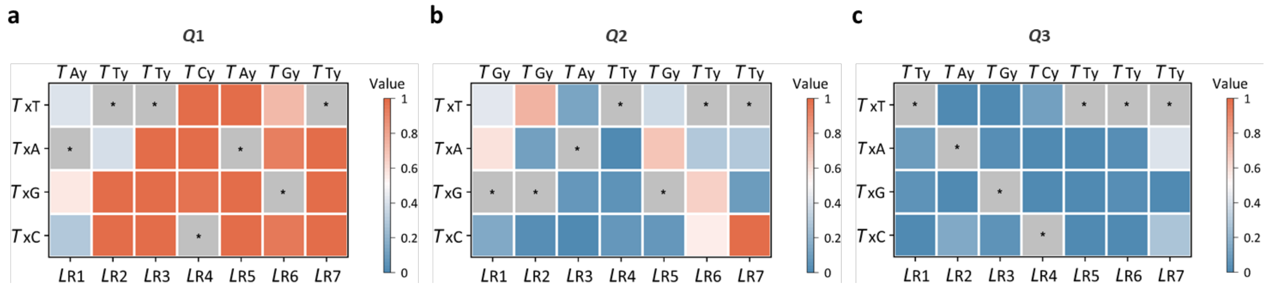

Supplementary Figure 1. Normalized fluorescence signal for activators with mismatch profiles described as $L_nT_{xy}Q_m$. (a-c) The heatmap shows the mismatch quantities of 1, 2, and 3 at the same location, respectively. Asterisks (*) indicate the crRNA-complementary nucleotide. L_n is the numeric location identifier of the substitution within the sequence, T_{xy} denotes the type of substitution where x is the original nucleotide and y is the mutated nucleotide, and Q_m indicates the quantity of substitutions.

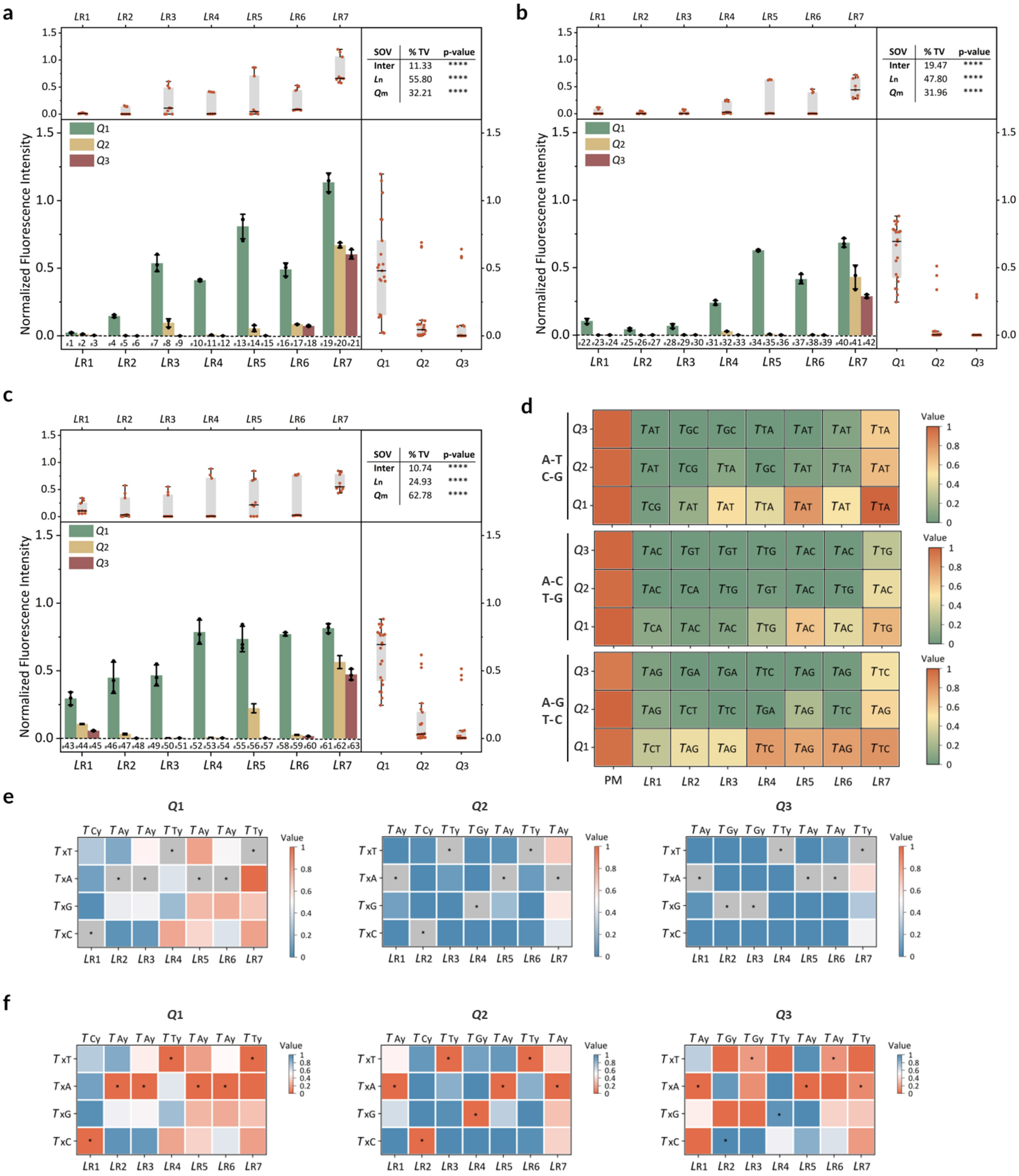

Supplementary Figure 2. Variability of fluorescence signal with mismatch profiles. (a-c) Normalized endpoint fluorescence signals for three types of nucleotide substitutions: (a) A-T/C-G, (b) A-C/T-G, and (c) A-G/T-C, with varying locations and quantities of mismatch sites. For each mismatch type, data are grouped by location (L_n) and mismatch quantity (Q_m) and presented as box plots. Box plots depict the median (middle line), interquartile range (box), and lower and upper adjacent values (whiskers), with

outliers represented as individual points. The upper-right region shows two-way ANOVA results. Data represent means ± s.d. (n = 3). SOV: source of variation; Inter: interaction; % TV: percentage of total variation. **** p-value < 0.0001. (d) Heatmap of the normalized fluorescence signal, annotated with the type of base substitution (T_{xy}), corresponding to panels (a)-(c). (e) Normalized fluorescence signal for activators with mismatch profiles described as $L_n T_{xy} Q_m$. The heatmap shows the mismatch quantities of 1, 2, and 3 at the same location, respectively. Asterisks (*) indicate the crRNA-complementary nucleotide. (f) Percentage of fluorescence signal decay for the activator with mismatches. The asterisk (*) indicates the crRNA-complementary nucleotide. The percentage of fluorescence signal decay = (M_{FS} - S_{FS})/ M_{FS}, where S_{FS} is the fluorescence signal for the crRNA-complementary nucleotide and M_{FS} is the fluorescence signal for the mutated activator.

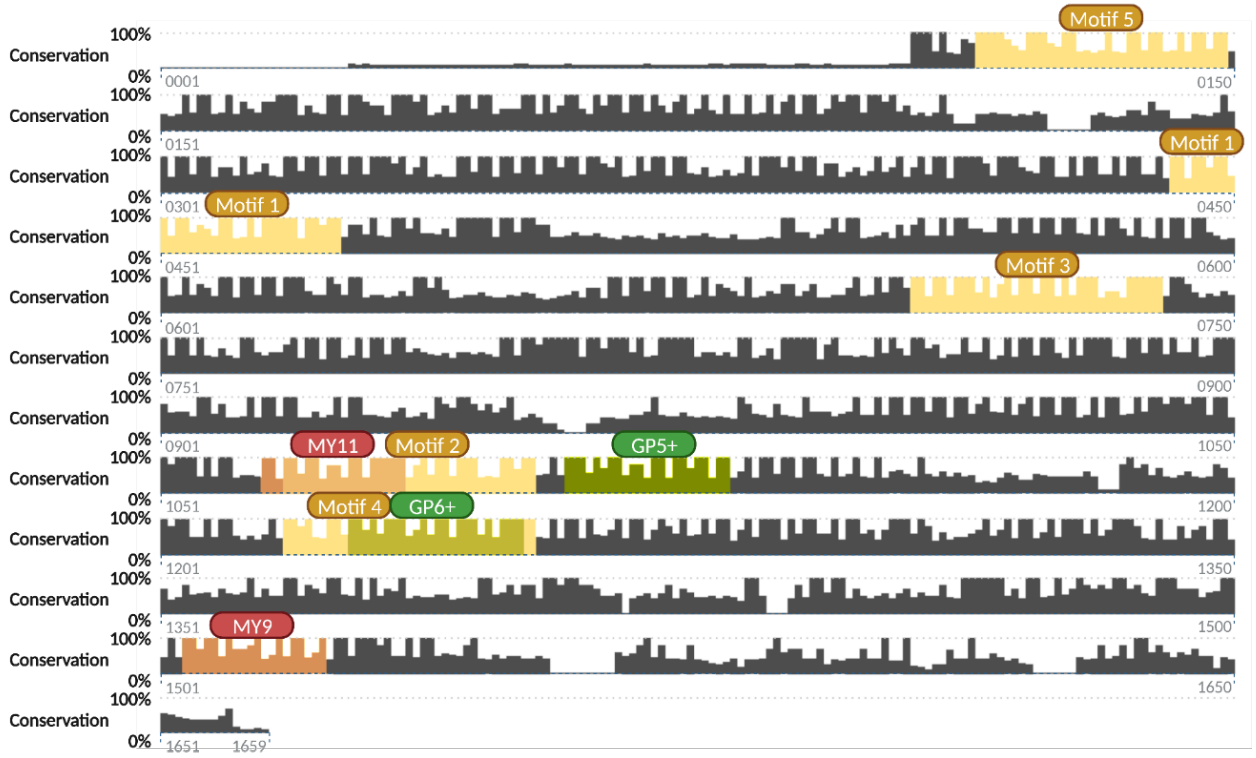

Supplementary Figure 3. Sequence alignment of the L1 regions of 14 HR-HPV subtypes. Gray bars represent the conservation percentage from 0% to 100% of each position. Colored bars indicate different subsequences, with motifs 1-5 highlighted in yellow, and the PCR primer pairs (GP5+/GP6+ and MY09/MY11) shown in green and red, respectively.

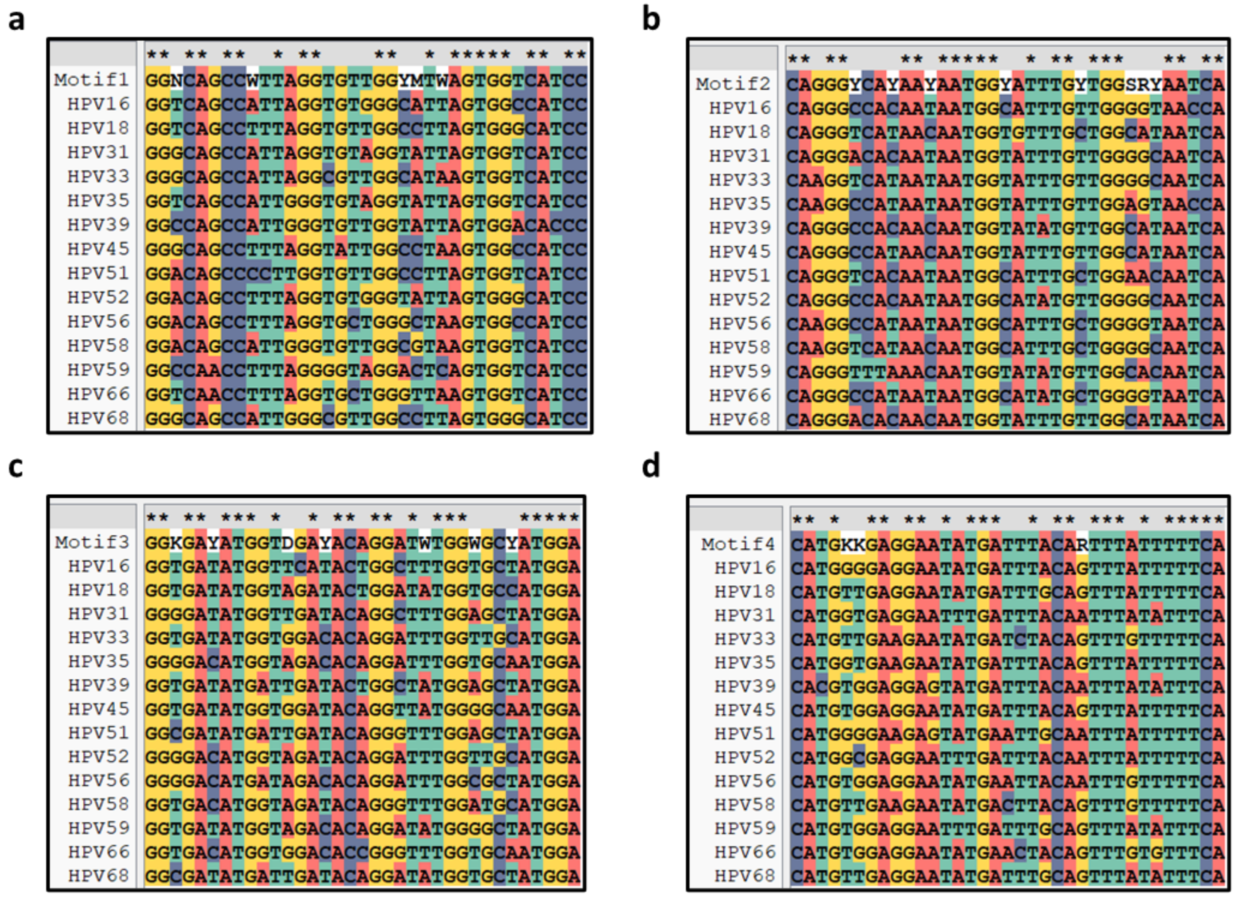

Supplementary Figure 4. Alignment of motifs 1-4 and sequences for each subtype of HR-HPV, corresponding to (a), (b), (c), and (d). Colors represent four different nucleotides, with asterisks (*) denoting positions with a single, fully conserved residue. The analysis was processed using ClustalX 2.1.[5]

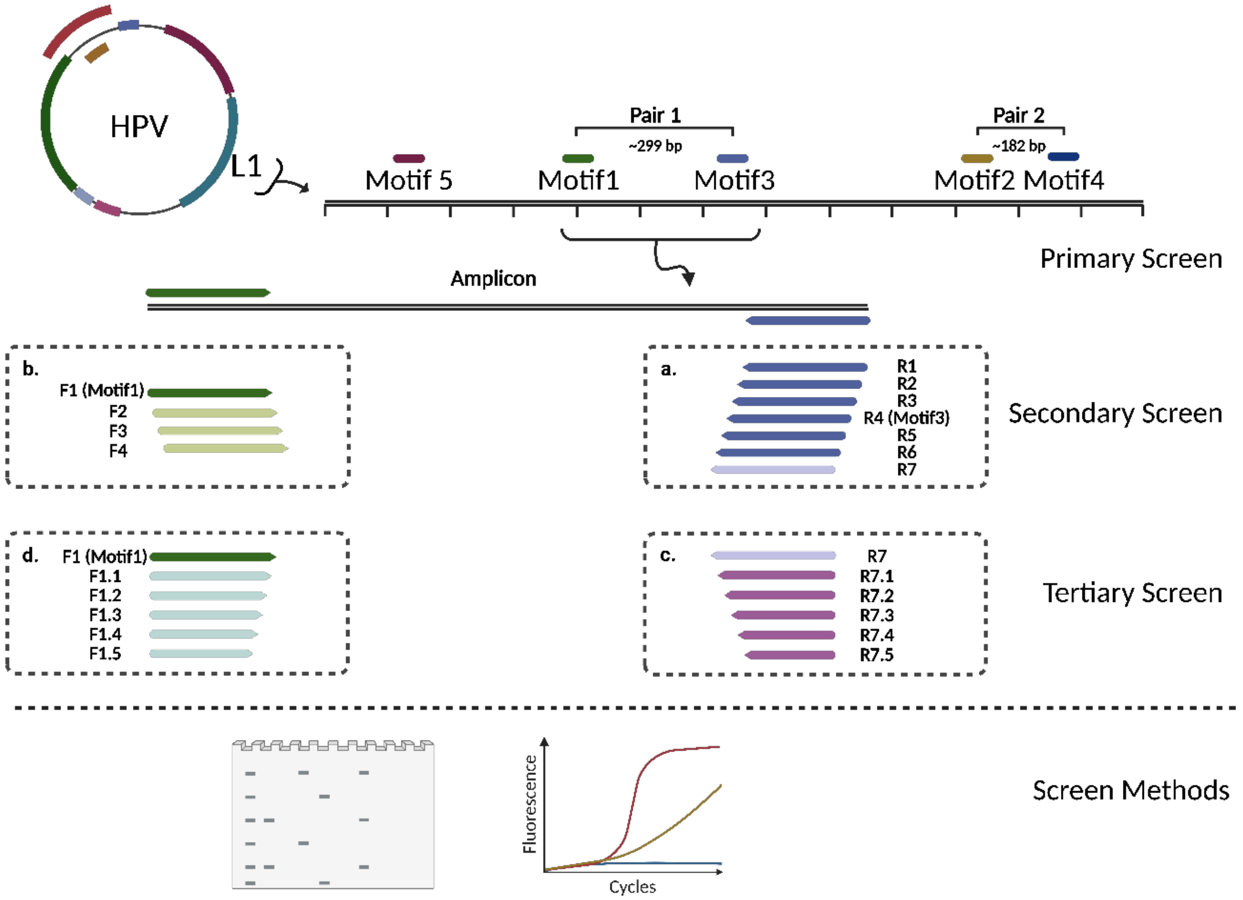

Supplementary Figure 5. Schematic illustrating the relative positions of motifs in HPV L1 identified through bioinformatics methods and the multi-phase primer optimization strategy. After primary screen, we began with the initial primer pair consisting of Motif1 (F1) and Motif3 (R4). (a) The first optimization step focused on Motif3, resulting in a series of new primers, designated as R1 through R7, each paired with the unchanged Motif1 (F1). (b) Following this, the first optimization of Motif1 was conducted, where R7 was fixed as the constant primer. This led to the development of four new primers, F1 through F4, each paired with R7. (c) Subsequently, we performed a second optimization on R7 while fixing F1, producing five new primers named R7.1 through R7.5. (d) Finally, we fixed R7 and carried out a second optimization on F1, resulting in the creation of five new primers, F1.1 through F1.5, each paired with R7. Screening methods involve gel electrophoresis and CRISPR-Cas12 *trans*-cleavage assays. Created in BioRender. Guan, X. (2024) https://BioRender.com/l24f526.

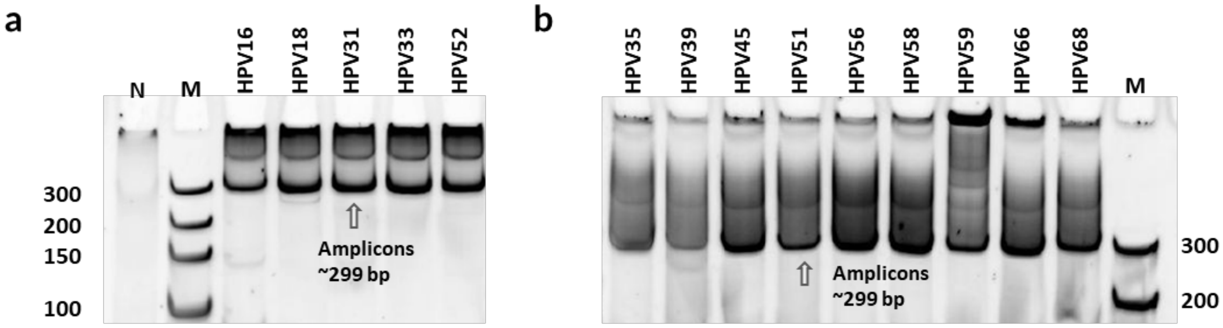

Supplementary Figure 6. Gel electrophoresis results of RPA products using the Motif 1–Motif 3 primer pair. (a) The image shows the RPA products for HPV16, HPV18, HPV31, HPV33 and HPV52. (b) The image shows the RPA products for HPV35, HPV39, HPV45, HPV51, HPV56, HPV58, HPV59, HPV66 and HPV68. Arrows indicate the position of the amplicons, which are about 299 bp in size. The concentration of the target plasmid is 10 nM. N: negative control; M: marker.

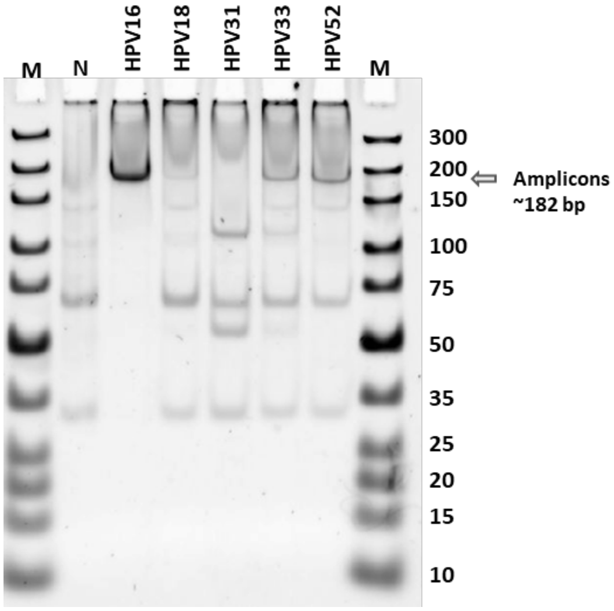

Supplementary Figure 7. Gel electrophoresis results for the primary screening step of primer selection. The amplicons of RPA using the Motif 2-Motif 4 primer pair. Arrows indicate the position of the amplicons, which are about 182 bp in size. The concentration of the target plasmid is 10 nM. N: negative control; M: marker.

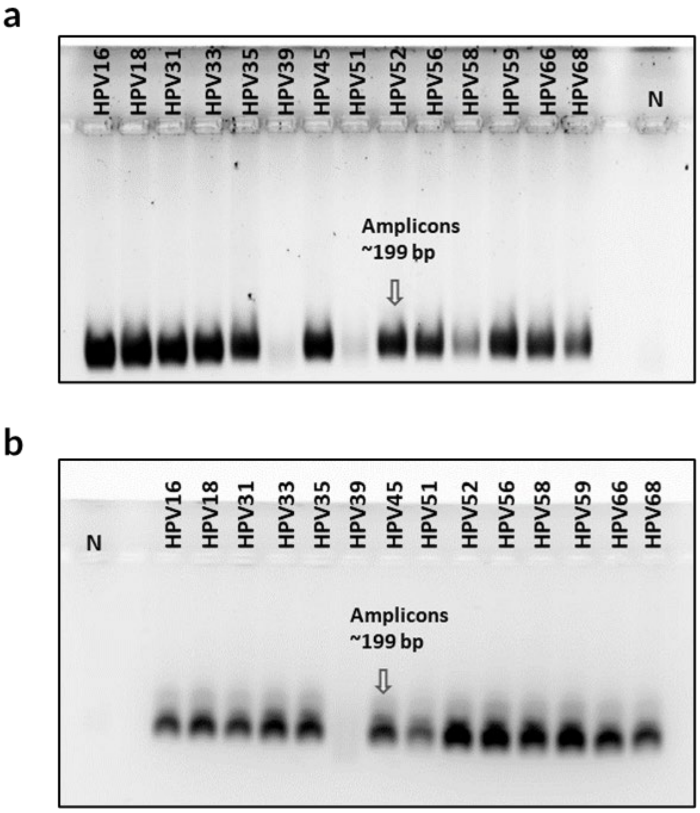

Supplementary Figure 8. Gel electrophoresis results of RPA reaction products with various primer sets. (a) Gel electrophoresis of RPA amplicons using the F11-R7 primer set. (b) Gel electrophoresis of RPA amplicons using the F12-R7 primer set. N: negative control.

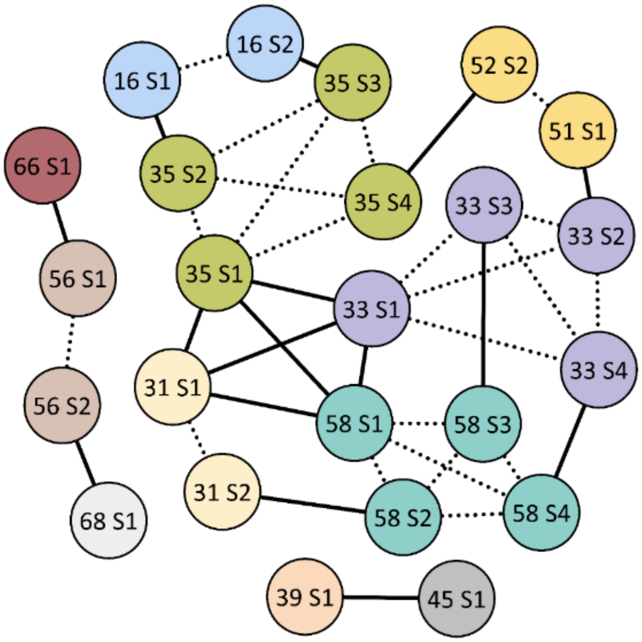

Supplementary Figure 9. Network plot illustrating the pairwise alignment results of HPV subtypes. Circles filled with the same color represent subsequences from the same subtype, connected by dotted lines. Solid lines connect perfectly matched subsequences. The text inside the circles indicates the HPV subtype and the number of subsequences within the sequence.

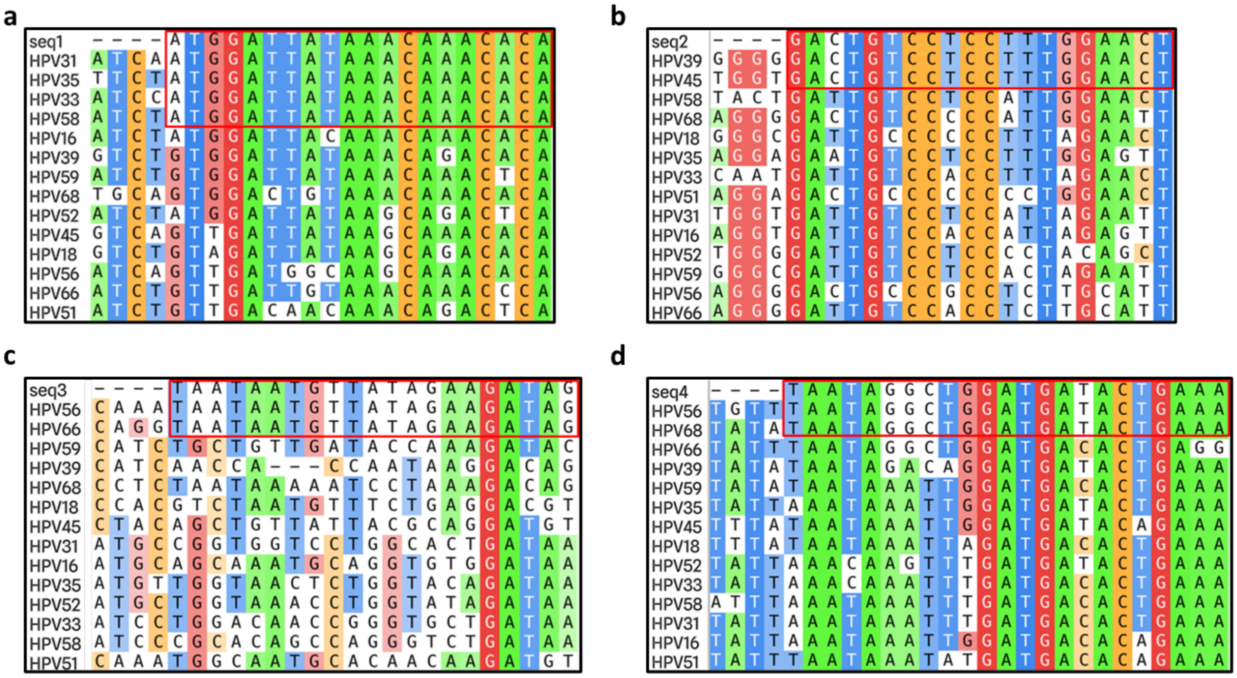

Supplementary Figure 10. Alignment of Seq1 through Seq4 with all subtypes of HR-HPV, corresponding to (a), (b), (c), and (d). Red frames are used to highlight the perfectly matched target sequences of the crRNAs. The analysis was processed using MAFFT version 7 (https://mafft.cbrc.jp/alignment/server/) [6].

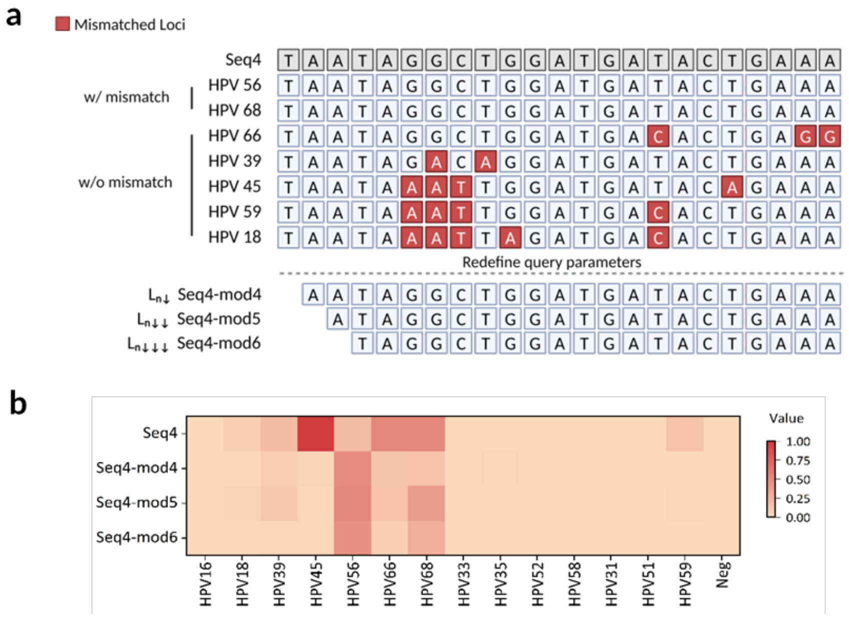

Supplementary Figure 11. Modification was performed on the crRNA targeting Seq4. Seq4-mod4, Seq4-mod5, and Seq4-mod6 were altered by decreasing the n value of L_n, which denotes the displacement of the mismatch site incrementally closer to the 5' end of the crRNA by one base. (a) Alignment of Seq4 with HPV subtypes that match perfectly (HPV56, 68) and those that have mismatches (HPV66, 39, 45, 59, 18). The modified crRNAs, Seq4-mod4 through Seq4-mod6, utilize distinct strategic approaches. Created in BioRender. Guan, X. (2024) https://BioRender.com/n89k691. (b) Heatmap presenting normalized detection outcomes for the 14 HR-HPV subtypes using modified crRNAs targeting Seq4.

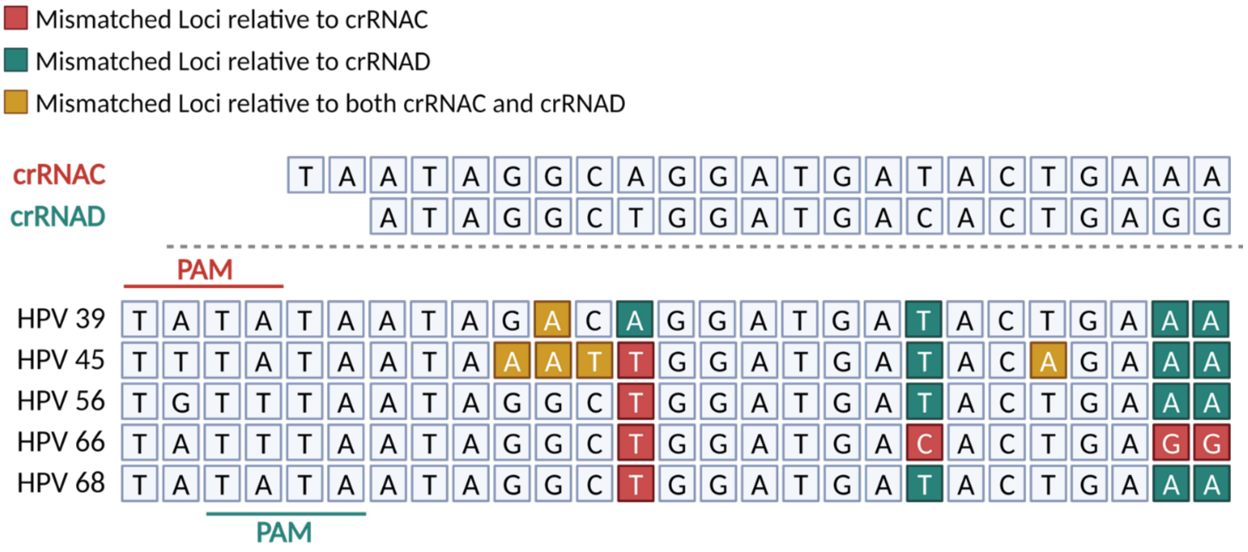

Supplementary Figure 12. Alignment of crRNA C and crRNA D with HPV subtypes HPV39, 45, 56, 66 and 68. Created in BioRender. Guan, X. (2024) https://BioRender.com/d88x635.

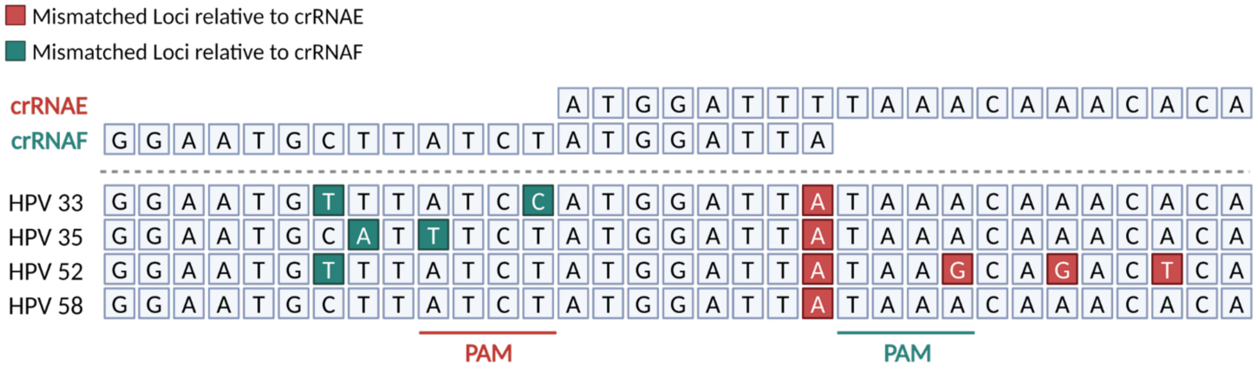

Supplementary Figure 13. Alignment of crRNA E and crRNA F with HPV subtypes HPV33, 35, 52 and 58. Created in BioRender. Guan, X. (2024) https://BioRender.com/k01c506.

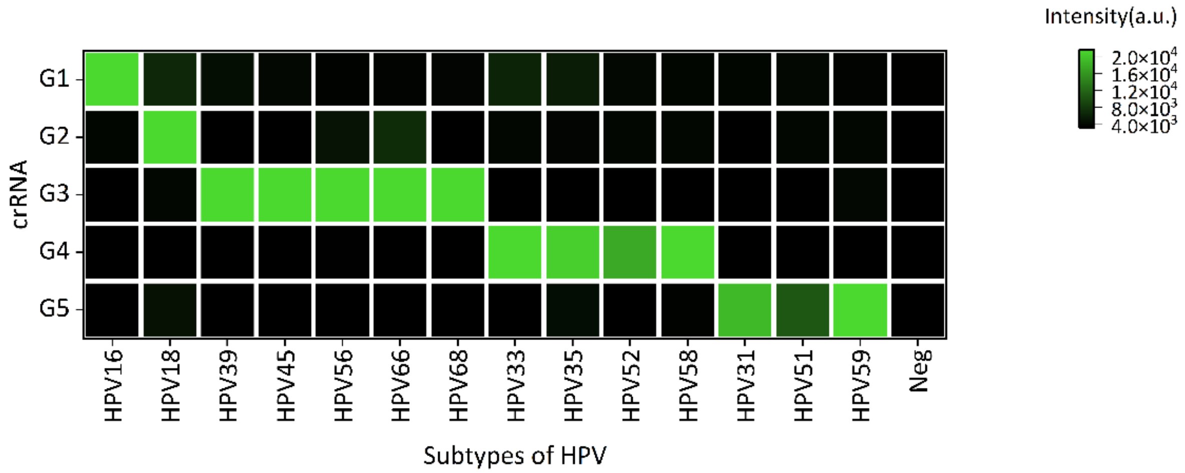

Supplementary Figure 14. The heatmap depicts the final fluorescence signals obtained while detecting 14 subtypes of HR-HPV using five groups of crRNAs labeled G1, G2, G3, G4, and G5. Within G3, G4, and G5, multiple crRNAs were included, which were pre-mixed in equal proportions prior to the experiment. The resulting mixture was then used for detecting each subtype, ensuring that the concentration of the mixed crRNAs matched that of the individual crRNA detection assays.

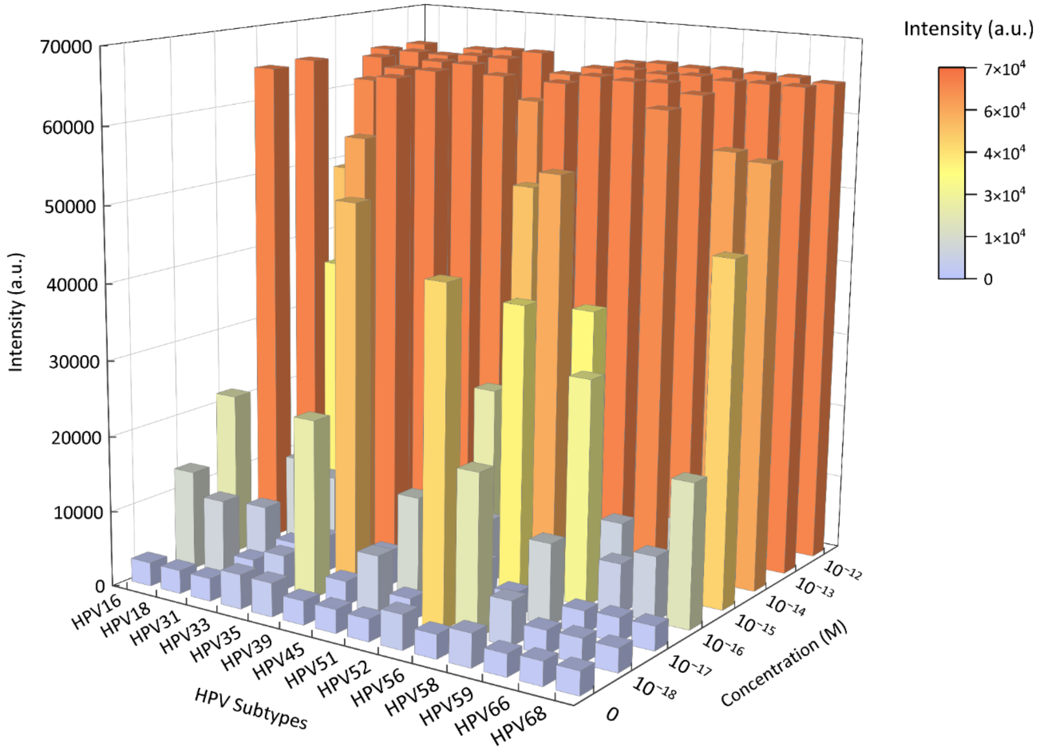

Supplementary Figure 15. 3D bar plot illustrates the sensitivity tests for all 14 HR-HPV subtypes.

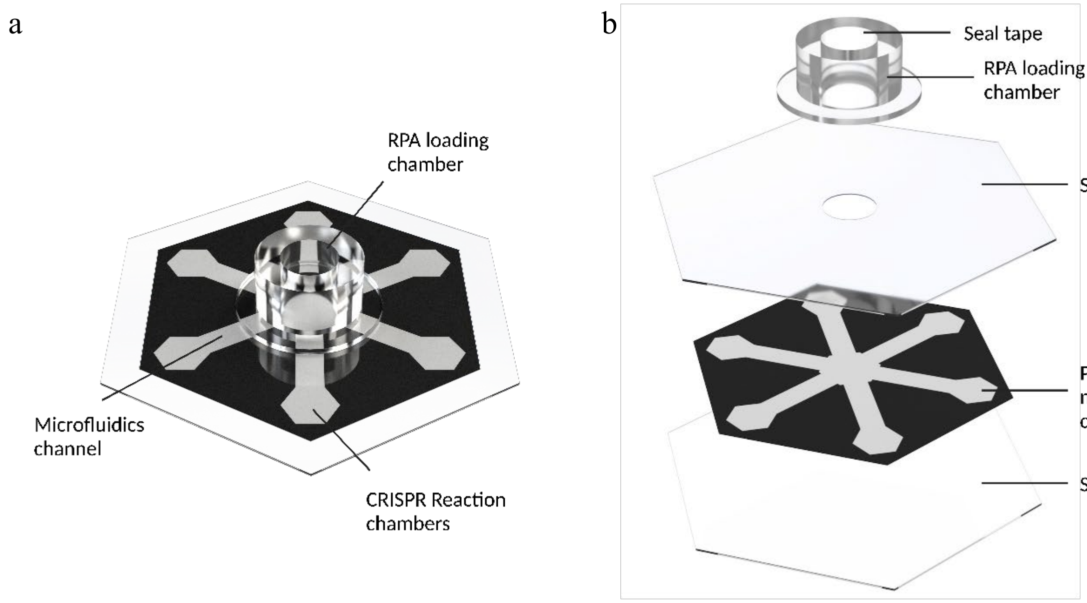

Supplementary Figure 16. Representation of the paper-based microfluidic chip. The chip's dimensions include a branch width and length of approximately 1 mm and 7 mm, respectively. The diameter of each unit and the central loading chamber is around 3 mm and 4.5 mm, respectively, depending on the melting conditions of the printed wax on the paper. (a) Schematic overview of the chip's reaction chambers. The chip includes an RPA loading chamber, six microfluidic channels, and six CRISPR reaction chambers. (b) Schematic showing the layers of the chip, including the seal tape combined with the RPA loading chamber and two layers of seal tape for the paper-based microfluidics chamber for the CRISPR-Cas12a reaction. These figures were produced using SolidWorks 2022.

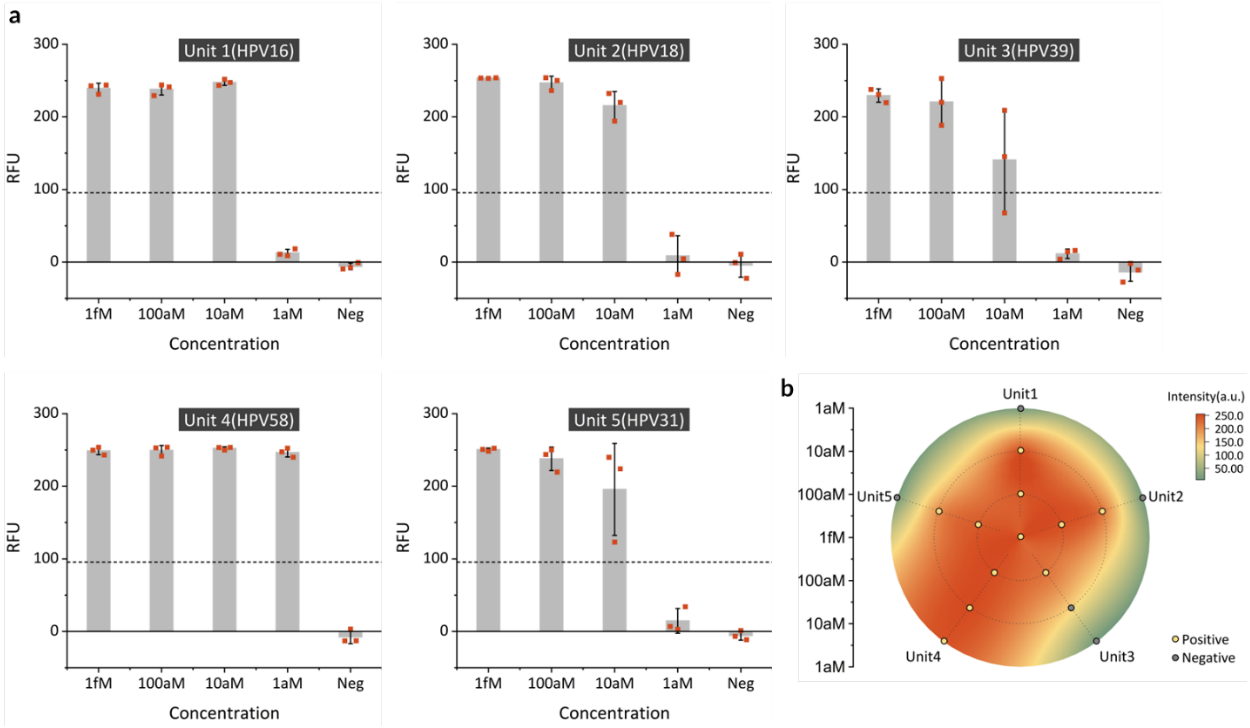

Supplementary Figure 17. Sensitivity analysis of the paper-based microfluidic chip. (a) Results of sensitivity testing for each unit of the paper-based microfluidic chip targeting HPV subtypes 16, 18, 39, 58, and 31. The assay utilized CRISPR-Cas12a detection on paper following RPA amplification of HPV DNA plasmids across a range of concentrations, from 1 fM to 1 aM. The dashed line represents the cut-off value set at 95.38, determined using the Youden Index. Data represent means ± s.d. (n = 3). Tests were performed at concentrations of 1fM, 100aM, 10aM, and 1aM, with three independent replicates for each concentration. Neg: negative. (b) Polar contour plot illustrating sensitivity results across all units. Each concentric ring on the plot corresponds to a different concentration level, while the radial lines represent different detection units. The colored dots on the plot indicate whether the detection result for each unit at the respective concentration is judged as positive or negative, based on the cut-off value. The plot has been smoothed for improved visualization.

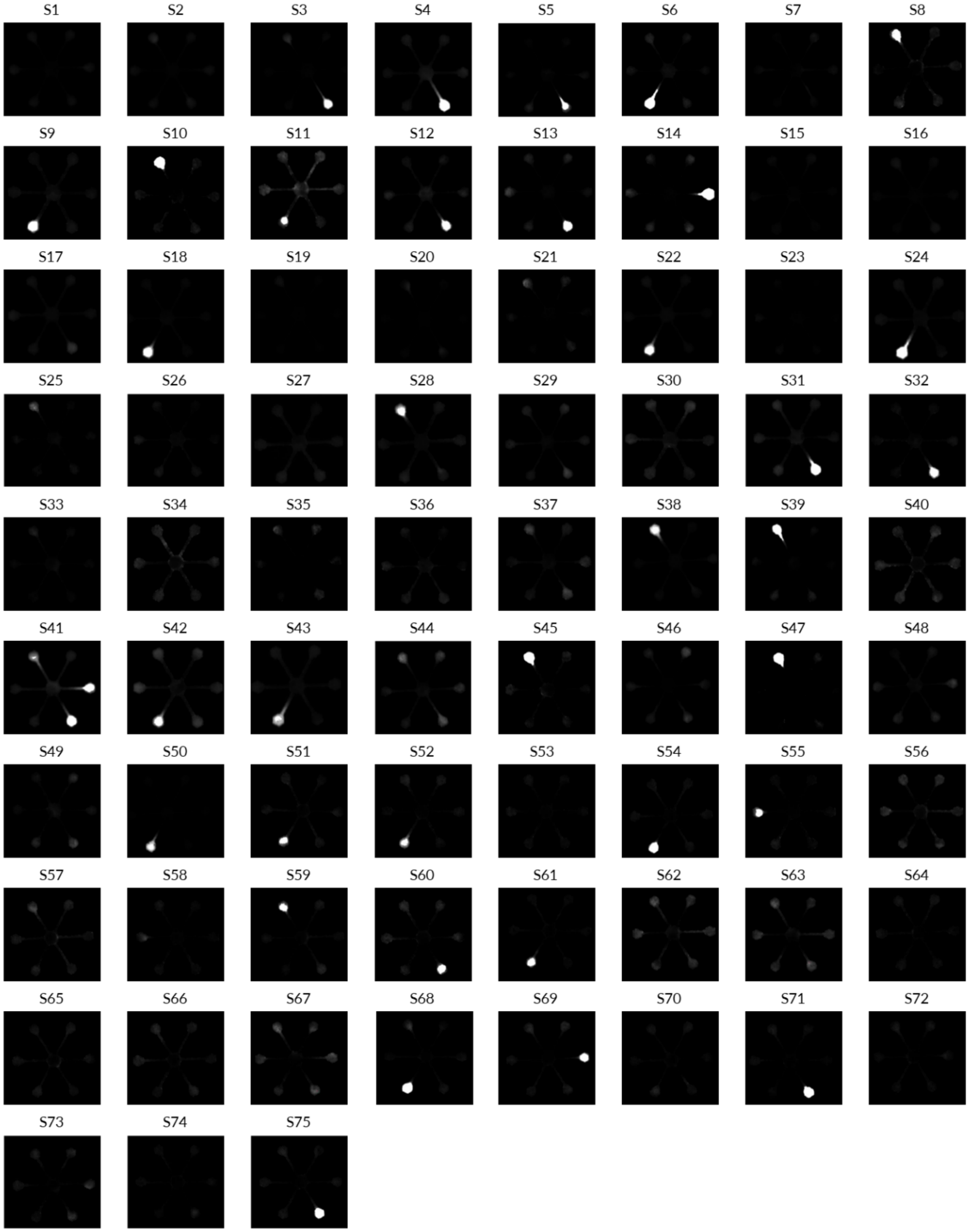

Supplementary Figure 18. Final fluorescence images captured from the platform, showcasing results obtained from 75 clinical samples.

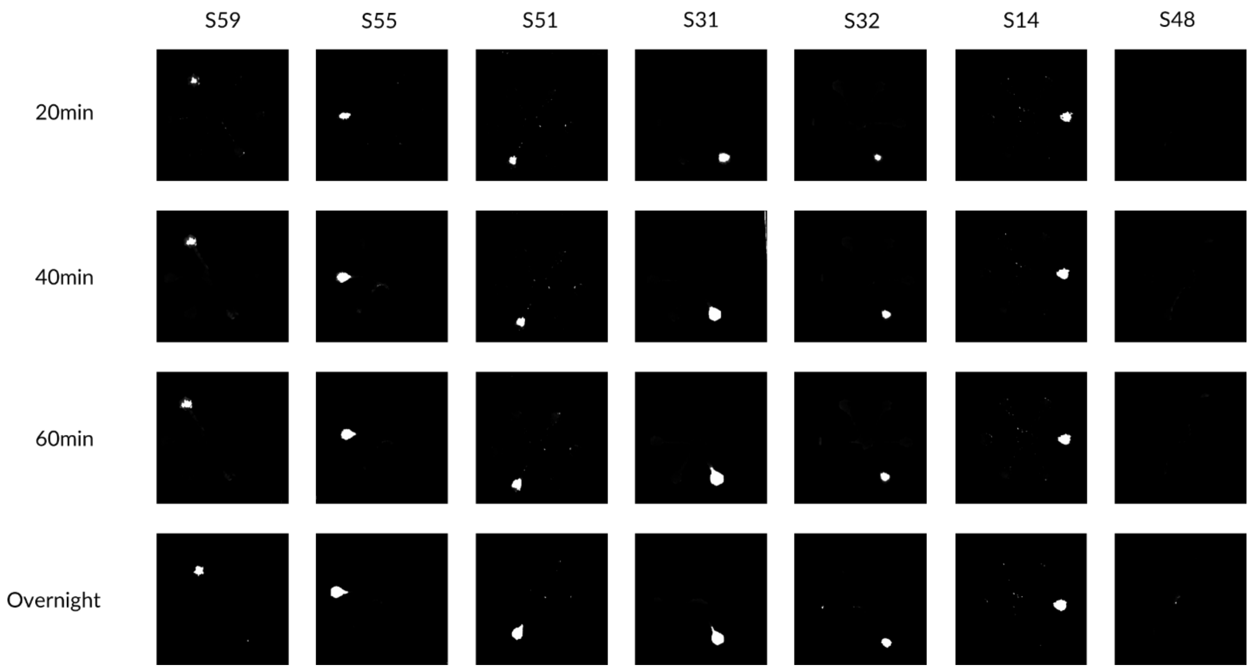

Supplementary Figure 19. Validation of system specificity and false positive potential using seven clinical samples (one negative, six positive) on a paper-based microfluidic chip (S59, S55, S51, S31, S32, S14, S48). Fluorescence images were taken at 20, 40, 60 minutes, and after overnight storage at room temperature.

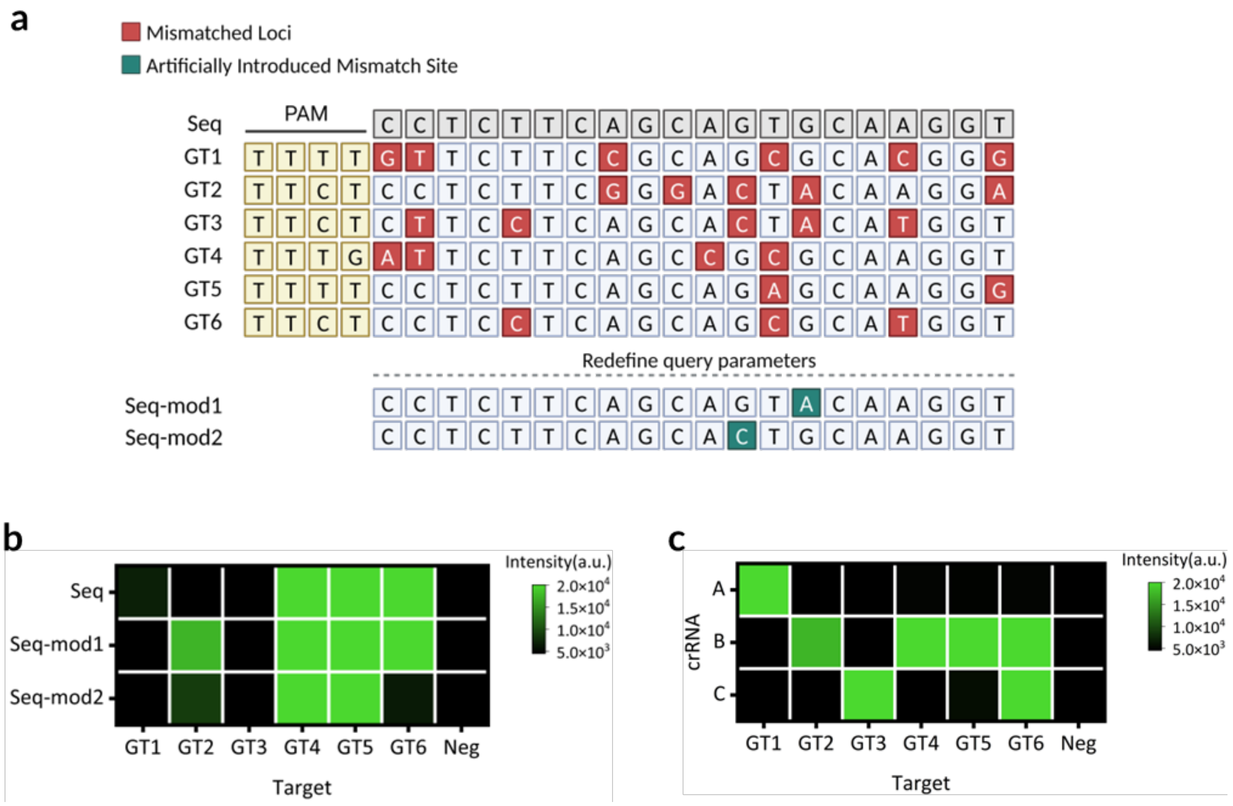

Supplementary Figure 20. Validation of the CRISPR-Cas12a assay for hepatitis C virus (HCV) detection. The assay was designed to accurately identify HCV genotype 1 and provide pooled detection for genotypes 2, 3, 4, 5, and 6. Mismatch profiles were utilized to prevent cross-reactivity with genotype 1, ensuring precise and efficient detection across multiple genotypes. (a) Alignment of the conserved sequence (Seq) identified in the NS5B region with HCV genotypes 1 through 6 (abbreviated as GT1 to GT6), using the bioinformatics tool MEME[7]. The modified crRNAs, Seq-mod1 and Seq-mod2, employ distinct strategic approaches. Created in BioRender. Guan, X. (2024) https://BioRender.com/c07e498. (b) Heatmap showing the fluorescence signals generated during the detection of the six HCV genotypes with and without modified crRNAs. (c) Heatmap displaying the fluorescence signals from the detection of the six HCV genotypes using modified crRNAs. crRNA A is specifically designed to detect genotype 1 (GT1) exclusively. crRNA B corresponds to Seq-mod1, which was modified to prevent cross-reactivity with GT1 while still allowing for pooled detection of other genotypes. crRNA C is a complementary crRNA developed to address the signal loss for genotype 3 (GT3). This crRNA is in the same NS5B region as crRNA B but has a sequence that is more closely aligned with GT3, thereby improving detection sensitivity for this genotype.

Supplementary Table 1. Target subtypes of each crRNA and corresponding PAM/suboptimal PAM sequences.

	Target1	***PAM Seq1***	***Target2***	***PAM Seq2***	***Target3***	***PAM Seq3***	***Target4***	***PAM Seq4***	***Target5***	***PAM Seq5***
crRNA A	HPV16	CCTA								
crRNA B	HPV18	GCCG								
crRNA C	HPV39	TATA	HPV45	TTTA	HPV56	TGTT	HPV66	TATT	HPV68	TATA
crRNA D	HPV66	TTTA	HPV56	TTTA	HPV68	TATA				
crRNA E	HPV33	ATCC	HPV35	TTCT	HPV58	ATCT				
crRNA F	HPV58	TTTA	HPV33	TTTA	HPV52	CTTA				
crRNA G	HPV35	TTTA								
crRNA H	HPV59	CTTG								
crRNA I	HPV51	TTTT								
crRNA J	HPV31	TTTG	HPV59	TTGG						

Supplementary Table 2. The Cq values for each sample for the 14 high-risk HPV subtypes and GAPDH.

HPV	***16***	***18***	***31***	***33***	***35***	***39***	***45***	***51***	***52***	***56***	***58***	***59***	***66***	***68***	***GAP DH***
S1															29.26
S2															24.65
S3									25.37						24.09
S4											28.12				22.56
S5											25.22		33.03		24.17
S6						28.4									24.48
S7															26.63
S8	34.16														24.43
S9										31.59			27.09		26.03
S10	29.11														24.14
S11									34.09					24.42	21.46
S12									27.37						25.22
S13									29.1						25.08
S14			28.95												24.68
S15															28.00
S16															29.19
S17															24.19
S18													24.82		21.53
S19															24.59
S20															25.25
S21															27.04
S22										24.29					22.99
S23															29.70
S24	33.74									24.65					23.73
S25															26.33
S26															24.08
S27															24.88
S28	33.3														23.62
S29															29.74
S30															25.01
S31					28.95										25.69
S32											29.13				24.79
S33															29.65
S34															28.89
S35															29.54
S36															27.38
S37															23.77
S38	34.98														22.61
S39	31.94														23.64
S40															28.15

S41					28.3			27.17					31.32		23.40
S42													29.44		22.61
S43													27.01		22.21
S44															25.18
S45	33.21														26.86
S46															25.14
S47	28.71														22.72
S48															24.13
S49															25.51
S50													27.77		25.09
S51							18.86								21.78
S52										29.2					23.34
S53															28.68
S54										25.02					23.11
S55		29.83					33.37								25.40
S56															28.40
S57															27.09
S58															27.17
S59	33.14														28.71
S60					34.31										27.05
S61	34.17									23.64					22.71
S62															29.13
S63															26.84
S64															29.10
S65															28.63
S66															29.73
S67															29.22
S68													24.79		23.55
S69			24.25												21.41
S70															24.60
S71											25.31				24.29
S72															24.82
S73															28.78
S74															24.10
S75					25.95									34.84	21.26

Supplementary Table 3. Samples with multiple subtypes of HR-HPV infection.

Sample	***Pos***	***Pos_Cq***	***Pos***	***Pos_Cq***	***Neg***	***Neg_Cq***
S5	U4	25.22			U3	33.03
S9	U3	31.59	U3	27.09		
S11	U3	24.42			U4	34.09
S24	U3	24.65			U1	33.74
S41	U4	28.3	U5	27.17	U3	31.32
S55	U2	29.83			U3	33.37
S61	U3	23.64			U1	34.17
S75	U4	25.95			U3	34.84

* Pos: Indicates the unit of the chip producing a positive detection result, with Pos_Cq representing the corresponding Cq value for the detected subtype of HPV. Neg: Indicates the unit of the chip producing a negative detection result, with Neg_Cq representing the corresponding Cq value for the undetected subtype of HPV.

Supplementary Table 4. The Cq values for each sample corresponding to each detection unit (a total of five detection units plus a blank control). The Cq value is derived from qPCR results; if multiple HPV subtypes are present in a single detection unit, the lower Cq value (indicating higher concentration) is recorded. The RFU (relative fluorescence units) is calculated by obtaining the fluorescence signal value for each unit on the chip using ImageJ, and then subtracting the fluorescence value of the blank control unit from each unit's fluorescence value to obtain the final RFU value. The P/N value indicates whether the sample is positive (1) or negative (0), determined using the paper-based chip. The cut-off value, calculated using the Youden Index, is 162.

	Unit1			***Unit2***			***Unit3***			***Unit4***			***Unit5***		
	**Cq**	**RFU**	**P/N**	**Cq**	**RFU**	**P/N**	**Cq**	**RFU**	**P/N**	**Cq**	**RFU**	**P/N**	**Cq**	**RFU**	**P/N**
S1		0	0		1	0		-1	0		-2	0		4	0
S2		25	0		8	0		1	0		1	0		2	0
S3		33	0		-5	0		-3	0	25.37	245	1		-6	0
S4		1	0		-7	0		-5	0	28.12	232	1		6	0
S5		-8	0		-4	0	33.03	16	0	25.22	241	1		3	0
S6		14	0		0	0	28.4	232	1		6	0		-7	0
S7		-15	0		-9	0		-15	0		-7	0		-8	0
S8	34.16	214	1		-24	0		48	0		19	0		-4	0
S9		-4	0		-2	0	27.09	235	1		1	0		-2	0
S10	29.11	199	1		-27	0		-23	0		-24	0		-24	0
S11		3	0		1	0	24.42	190	1	34.09	-22	0		-10	0
S12		-9	0		8	0		3	0	27.37	229	1		7	0
S13		-4	0		27	0		-7	0	29.1	218	1		-20	0
S14		-14	0		6	0		-10	0		12	0	28.95	207	1
S15		-3	0		-3	0		-6	0		-8	0		-8	0
S16		7	0		-4	0		-5	0		8	0		0	0
S17		0	0		5	0		-1	0		33	0		-3	0
S18		0	0		0	0	24.82	247	1		0	0		0	0
S19		10	0		-2	0		-1	0		-2	0		-2	0
S20		23	0		-2	0		0	0		18	0		-3	0
S21		55	0		-20	0		-32	0		-10	0		-20	0
S22		3	0		-1	0	24.29	243	1		-2	0		-4	0
S23		5	0		11	0		9	0		4	0		4	0
S24	33.74	1	0		0	0	24.65	237	1		-2	0		-2	0
S25		134	0		3	0		-3	0		5	0		-3	0
S26		10	0		22	0		17	0		10	0		9	0
S27		9	0		7	0		14	0		15	0		7	0
S28	33.3	233	1		-4	0		7	0		17	0		2	0
S29		-15	0		-15	0		-23	0		20	0		-16	0
S30		-9	0		-9	0		-21	0		-28	0		-26	0
S31		18	0		8	0		19	0	28.95	232	1		-5	0
S32		-4	0		4	0		0	0	29.13	235	1		0	0
S33		21	0		-2	0		-4	0		-4	0		-2	0

S34		5	0		0	0		-13	0		-20	0		-11	0
S35		18	0		-32	0		-36	0		-15	0		-27	0
S36		7	0		22	0		8	0		15	0		12	0
S37		43	0		-4	0		-1	0		43	0		31	0
S38	34.98	247	1		0	0		2	0		8	0		7	0
S39	31.94	241	1		2	0		16	0		14	0		-8	0
S40		-31	0		-12	0		-2	0		-8	0		6	0
S41		228	1		-5	0	31.32	-4	0	28.3	228	1	27.17	228	1
S42		9	0		19	0	29.44	211	1		8	0		-12	0
S43		2	0		0	0	27.01	236	1		-4	0		-3	0
S44		49	0		-40	0		-41	0		20	0		-20	0
S45	33.21	222	1		10	0		-9	0		2	0		-16	0
S46		-35	0		-41	0		-43	0		-14	0		-45	0
S47	28.71	227	1		-8	0		0	0		1	0		-18	0
S48		-9	0		8	0		7	0		14	0		33	0
S49		-26	0		-32	0		-7	0		31	0		-22	0
S50		-1	0		-1	0	27.77	250	1		0	0		-2	0
S51		29	0		16	0	18.86	250	1		15	0		18	0
S52		27	0		9	0	29.2	250	1		11	0		14	0
S53		13	0		15	0		13	0		13	0		9	0
S54		17	0		12	0	25.02	250	1		13	0		13	0
S55		8	0	29.83	250	1	33.37	25	0		13	0		15	0
S56		45	0		63	0		36	0		49	0		32	0
S57		67	0		21	0		26	0		20	0		25	0
S58		15	0		59	0		13	0		12	0		9	0
S59	33.14	250	1		12	0		6	0		31	0		10	0
S60		40	0		35	0		27	0	34.31	250	1		36	0
S61	34.17	9	0		7	0	23.64	250	1		7	0		8	0
S62		77	0		65	0		38	0		44	0		39	0
S63		79	0		48	0		37	0		89	0		37	0
S64		16	0		15	0		15	0		9	0		10	0
S65		18	0		16	0		21	0		10	0		18	0
S66		34	0		28	0		25	0		30	0		13	0
S67		89	0		47	0		26	0		80	0		74	0
S68		51	0		5	0	24.79	250	1		16	0		10	0
S69		36	0		17	0		13	0		22	0	24.25	251	1
S70		15	0		7	0		31	0		19	0		7	0
S71		21	0		9	0		5	0	25.31	250	1		4	0
S72		20	0		7	0		10	0		10	0		19	0
S73		25	0		18	0		15	0		42	0		50	0
S74		8	0		7	0		4	0		27	0		10	0
S75		29	0		17	0	34.84	18	0	25.95	250	1		16	0

Supplementary Table 5. The cost of the 14 HR-HPV detection chips.

RPA		***Price per kit***	***Volume/Reaction per kit***	***Volume per reaction***	***Cost per reaction***
	TwistAmp® Basic	$443.00	96 reactions	0.5 reaction	$2.3000
	Primer F	$5.57	2,500 µL	1.2 µL	$0.0027
	Primer R	$6.09	2,500 µL	1.2 µL	$0.0029
CRISPR		***Price per kit***	***Volume/Reaction per kit***	***Volume per reaction***	***Cost per reaction***
	LbCas12a	$913	837.5 µL	0.25 µL	$0.2725
	crRNA	$132.30	2,500 µL	0.4 µL	$0.0212
	ssDNA-FQ	$682.7	20,000 µL	0.5 µL	$0.0171
				Total:	$2.62

3. Nucleic acid sequences

Supplementary Table 6. Nucleotide sequences of mismatch profiles, used in the Figure 1. The sequences shown are from HPV16 and were randomly selected for illustration purposes.

Name	***Sequence (5'→3')***
crRNA	UAAUUUCUACUAAGUGUAGAUCUACAUUACAGGCUAACAAAA
PM	AGCCCTATTAAATAAATGTCTAACAAACATTTGTTCCCTTCGTAAATAAAAAAATA<br>AGCTGTCGCCATATGGTTCTGACACCATTTTAATATAATCTGGATATTTGCAAATAG<br>ATGTACAAATATCCAGTGGAACTTCACTTTTGTTAGCCTGTAATGTAGTAAAGTCCA<br>TAGCACCAAAGCCAGTATGAACCATATCACCATCCTGAATAACTGTGTTTATTAAC<br>TCTAATGGTGGACAATCACCTGGATTTACTGCAACATTGGTACATGGGGATCCTTTG<br>CCCCAGTGTTCCCCTAT
#1	AGCCCTATTAAATAAATGTCTAACAAACATTTGTTCCCTTCGTAAATAAAAAAATA<br>AGCTGTCGCCATATGGTTCTGACACCATTTTAATATAATCTGGATATTTGCAAATAG<br>ATGTACAAATATCCAGTGGAACTTCACTTTTGTTAGCCTGTAATGT**T**GTAAAGTCCA<br>TAGCACCAAAGCCAGTATGAACCATATCACCATCCTGAATAACTGTGTTTATTAAC<br>TCTAATGGTGGACAATCACCTGGATTTACTGCAACATTGGTACATGGGGATCCTTTG<br>CCCCAGTGTTCCCCTAT
#2	AGCCCTATTAAATAAATGTCTAACAAACATTTGTTCCCTTCGTAAATAAAAAAATA<br>AGCTGTCGCCATATGGTTCTGACACCATTTTAATATAATCTGGATATTTGCAAATAG<br>ATGTACAAATATCCAGTGGAACTTCACTTTTGTTAGCCTGTAATGT**TC**TAAAGTCCA<br>TAGCACCAAAGCCAGTATGAACCATATCACCATCCTGAATAACTGTGTTTATTAAC<br>TCTAATGGTGGACAATCACCTGGATTTACTGCAACATTGGTACATGGGGATCCTTTG<br>CCCCAGTGTTCCCCTAT
#3	AGCCCTATTAAATAAATGTCTAACAAACATTTGTTCCCTTCGTAAATAAAAAAATA<br>AGCTGTCGCCATATGGTTCTGACACCATTTTAATATAATCTGGATATTTGCAAATAG<br>ATGTACAAATATCCAGTGGAACTTCACTTTTGTTAGCCTGTAATG**ATC**TAAAGTCCA<br>TAGCACCAAAGCCAGTATGAACCATATCACCATCCTGAATAACTGTGTTTATTAAC<br>TCTAATGGTGGACAATCACCTGGATTTACTGCAACATTGGTACATGGGGATCCTTTG<br>CCCCAGTGTTCCCCTAT
#4	AGCCCTATTAAATAAATGTCTAACAAACATTTGTTCCCTTCGTAAATAAAAAAATA<br>AGCTGTCGCCATATGGTTCTGACACCATTTTAATATAATCTGGATATTTGCAAATAG<br>ATGTACAAATATCCAGTGGAACTTCACTTTTGTTAGCCTGTAA**A**GTAGTAAAGTCC<br>ATAGCACCAAAGCCAGTATGAACCATATCACCATCCTGAATAACTGTGTTTATTAA<br>CTCTAATGGTGGACAATCACCTGGATTTACTGCAACATTGGTACATGGGGATCCTTT<br>GCCCCAGTGTTCCCCTAT
#5	AGCCCTATTAAATAAATGTCTAACAAACATTTGTTCCCTTCGTAAATAAAAAAATA<br>AGCTGTCGCCATATGGTTCTGACACCATTTTAATATAATCTGGATATTTGCAAATAG<br>ATGTACAAATATCCAGTGGAACTTCACTTTTGTTAGCCTGTAA**AC**TAGTAAAGTCC<br>ATAGCACCAAAGCCAGTATGAACCATATCACCATCCTGAATAACTGTGTTTATTAA<br>CTCTAATGGTGGACAATCACCTGGATTTACTGCAACATTGGTACATGGGGATCCTTT<br>GCCCCAGTGTTCCCCTAT
#6	AGCCCTATTAAATAAATGTCTAACAAACATTTGTTCCCTTCGTAAATAAAAAAATA<br>AGCTGTCGCCATATGGTTCTGACACCATTTTAATATAATCTGGATATTTGCAAATAG<br>ATGTACAAATATCCAGTGGAACTTCACTTTTGTTAGCCTGTA**TAC**TAGTAAAGTCCA<br>TAGCACCAAAGCCAGTATGAACCATATCACCATCCTGAATAACTGTGTTTATTAAC<br>TCTAATGGTGGACAATCACCTGGATTTACTGCAACATTGGTACATGGGGATCCTTTG<br>CCCCAGTGTTCCCCTAT
#7	AGCCCTATTAAATAAATGTCTAACAAACATTTGTTCCCTTCGTAAATAAAAAAATA

AGCTGTCGCCATATGGTTCTGACACCATTTTAATATAATCTGGATATTTGCAAATAG
ATGTACAAATATCCAGTGGAACTTCACTTTTGTTAGCCTG**A**AATGTAGTAAAGTCC
ATAGCACCAAAGCCAGTATGAACCATATCACCATCCTGAATAACTGTGTTTATTAA
CTCTAATGGTGGACAATCACCTGGATTTACTGCAACATTGGTACATGGGGATCCTTT
GCCCCAGTGTTCCCCTAT

#8
AGCCCTATTAAATAAATGTCTAACAAACATTTGTTCCCTTCGTAAATAAAAAAATA
AGCTGTCGCCATATGGTTCTGACACCATTTTAATATAATCTGGATATTTGCAAATAG
ATGTACAAATATCCAGTGGAACTTCACTTTTGTTAGCCTG**AT**ATGTAGTAAAGTCCA
TAGCACCAAAGCCAGTATGAACCATATCACCATCCTGAATAACTGTGTTTATTAAC
TCTAATGGTGGACAATCACCTGGATTTACTGCAACATTGGTACATGGGGATCCTTTG
CCCCAGTGTTCCCCTAT

#9
AGCCCTATTAAATAAATGTCTAACAAACATTTGTTCCCTTCGTAAATAAAAAAATA
AGCTGTCGCCATATGGTTCTGACACCATTTTAATATAATCTGGATATTTGCAAATAG
ATGTACAAATATCCAGTGGAACTTCACTTTTGTTAGCCT**CAT**ATGTAGTAAAGTCCA
TAGCACCAAAGCCAGTATGAACCATATCACCATCCTGAATAACTGTGTTTATTAAC
TCTAATGGTGGACAATCACCTGGATTTACTGCAACATTGGTACATGGGGATCCTTTG
CCCCAGTGTTCCCCTAT

#10
AGCCCTATTAAATAAATGTCTAACAAACATTTGTTCCCTTCGTAAATAAAAAAATA
AGCTGTCGCCATATGGTTCTGACACCATTTTAATATAATCTGGATATTTGCAAATAG
ATGTACAAATATCCAGTGGAACTTCACTTTTGTTAGC**G**TGTAATGTAGTAAAGTCC
ATAGCACCAAAGCCAGTATGAACCATATCACCATCCTGAATAACTGTGTTTATTAA
CTCTAATGGTGGACAATCACCTGGATTTACTGCAACATTGGTACATGGGGATCCTTT
GCCCCAGTGTTCCCCTAT

#11
AGCCCTATTAAATAAATGTCTAACAAACATTTGTTCCCTTCGTAAATAAAAAAATA
AGCTGTCGCCATATGGTTCTGACACCATTTTAATATAATCTGGATATTTGCAAATAG
ATGTACAAATATCCAGTGGAACTTCACTTTTGTTAGC**GA**GTAATGTAGTAAAGTCC
ATAGCACCAAAGCCAGTATGAACCATATCACCATCCTGAATAACTGTGTTTATTAA
CTCTAATGGTGGACAATCACCTGGATTTACTGCAACATTGGTACATGGGGATCCTTT
GCCCCAGTGTTCCCCTAT

#12
AGCCCTATTAAATAAATGTCTAACAAACATTTGTTCCCTTCGTAAATAAAAAAATA
AGCTGTCGCCATATGGTTCTGACACCATTTTAATATAATCTGGATATTTGCAAATAG
ATGTACAAATATCCAGTGGAACTTCACTTTTGTTAG**GGA**GTAATGTAGTAAAGTCC
ATAGCACCAAAGCCAGTATGAACCATATCACCATCCTGAATAACTGTGTTTATTAA
CTCTAATGGTGGACAATCACCTGGATTTACTGCAACATTGGTACATGGGGATCCTTT
GCCCCAGTGTTCCCCTAT

#13
AGCCCTATTAAATAAATGTCTAACAAACATTTGTTCCCTTCGTAAATAAAAAAATA
AGCTGTCGCCATATGGTTCTGACACCATTTTAATATAATCTGGATATTTGCAAATAG
ATGTACAAATATCCAGTGGAACTTCACTTTTGTT**T**GCCTGTAATGTAGTAAAGTCCA
TAGCACCAAAGCCAGTATGAACCATATCACCATCCTGAATAACTGTGTTTATTAAC
TCTAATGGTGGACAATCACCTGGATTTACTGCAACATTGGTACATGGGGATCCTTTG
CCCCAGTGTTCCCCTAT

#14
AGCCCTATTAAATAAATGTCTAACAAACATTTGTTCCCTTCGTAAATAAAAAAATA
AGCTGTCGCCATATGGTTCTGACACCATTTTAATATAATCTGGATATTTGCAAATAG
ATGTACAAATATCCAGTGGAACTTCACTTTTGTT**TC**CCTGTAATGTAGTAAAGTCCA
TAGCACCAAAGCCAGTATGAACCATATCACCATCCTGAATAACTGTGTTTATTAAC
TCTAATGGTGGACAATCACCTGGATTTACTGCAACATTGGTACATGGGGATCCTTTG
CCCCAGTGTTCCCCTAT

#15
AGCCCTATTAAATAAATGTCTAACAAACATTTGTTCCCTTCGTAAATAAAAAAATA
AGCTGTCGCCATATGGTTCTGACACCATTTTAATATAATCTGGATATTTGCAAATAG
ATGTACAAATATCCAGTGGAACTTCACTTTTGT**ATC**CCTGTAATGTAGTAAAGTCCA
TAGCACCAAAGCCAGTATGAACCATATCACCATCCTGAATAACTGTGTTTATTAAC

TCTAATGGTGGACAATCACCTGGATTTACTGCAACATTGGTACATGGGGATCCTTTG
CCCCAGTGTTCCCCTAT

#16 AGCCCTATTAAATAAATGTCTAACAAACATTTGTTCCCTTCGTAAATAAAAAAATA
AGCTGTCGCCATATGGTTCTGACACCATTTTAATATAATCTGGATATTTGCAAATAG
ATGTACAAATATCCAGTGGAACTTCACTTTTCTTAGCCTGTAATGTAGTAAAGTCCA
TAGCACCAAAGCCAGTATGAACCATATCACCATCCTGAATAACTGTGTTTATTAAC
TCTAATGGTGGACAATCACCTGGATTTACTGCAACATTGGTACATGGGGATCCTTTG
CCCCAGTGTTCCCCTAT

#17 AGCCCTATTAAATAAATGTCTAACAAACATTTGTTCCCTTCGTAAATAAAAAAATA
AGCTGTCGCCATATGGTTCTGACACCATTTTAATATAATCTGGATATTTGCAAATAG
ATGTACAAATATCCAGTGGAACTTCACTTTTCATAGCCTGTAATGTAGTAAAGTCC
ATAGCACCAAAGCCAGTATGAACCATATCACCATCCTGAATAACTGTGTTTATTAA
CTCTAATGGTGGACAATCACCTGGATTTACTGCAACATTGGTACATGGGGATCCTTT
GCCCCAGTGTTCCCCTAT

#18 AGCCCTATTAAATAAATGTCTAACAAACATTTGTTCCCTTCGTAAATAAAAAAATA
AGCTGTCGCCATATGGTTCTGACACCATTTTAATATAATCTGGATATTTGCAAATAG
ATGTACAAATATCCAGTGGAACTTCACTTTACATAGCCTGTAATGTAGTAAAGTCC
ATAGCACCAAAGCCAGTATGAACCATATCACCATCCTGAATAACTGTGTTTATTAA
CTCTAATGGTGGACAATCACCTGGATTTACTGCAACATTGGTACATGGGGATCCTTT
GCCCCAGTGTTCCCCTAT

#19 AGCCCTATTAAATAAATGTCTAACAAACATTTGTTCCCTTCGTAAATAAAAAAATA
AGCTGTCGCCATATGGTTCTGACACCATTTTAATATAATCTGGATATTTGCAAATAG
ATGTACAAATATCCAGTGGAACTTCACTATTGTTAGCCTGTAATGTAGTAAAGTCC
ATAGCACCAAAGCCAGTATGAACCATATCACCATCCTGAATAACTGTGTTTATTAA
CTCTAATGGTGGACAATCACCTGGATTTACTGCAACATTGGTACATGGGGATCCTTT
GCCCCAGTGTTCCCCTAT

#20 AGCCCTATTAAATAAATGTCTAACAAACATTTGTTCCCTTCGTAAATAAAAAAATA
AGCTGTCGCCATATGGTTCTGACACCATTTTAATATAATCTGGATATTTGCAAATAG
ATGTACAAATATCCAGTGGAACTTCACTAATGTTAGCCTGTAATGTAGTAAAGTCC
ATAGCACCAAAGCCAGTATGAACCATATCACCATCCTGAATAACTGTGTTTATTAA
CTCTAATGGTGGACAATCACCTGGATTTACTGCAACATTGGTACATGGGGATCCTTT
GCCCCAGTGTTCCCCTAT

#21 AGCCCTATTAAATAAATGTCTAACAAACATTTGTTCCCTTCGTAAATAAAAAAATA
AGCTGTCGCCATATGGTTCTGACACCATTTTAATATAATCTGGATATTTGCAAATAG
ATGTACAAATATCCAGTGGAACTTCACAAATGTTAGCCTGTAATGTAGTAAAGTCC
ATAGCACCAAAGCCAGTATGAACCATATCACCATCCTGAATAACTGTGTTTATTAA
CTCTAATGGTGGACAATCACCTGGATTTACTGCAACATTGGTACATGGGGATCCTTT
GCCCCAGTGTTCCCCTAT

#22 AGCCCTATTAAATAAATGTCTAACAAACATTTGTTCCCTTCGTAAATAAAAAAATA
AGCTGTCGCCATATGGTTCTGACACCATTTTAATATAATCTGGATATTTGCAAATAG
ATGTACAAATATCCAGTGGAACTTCACTTTTGTTAGCCTGTAATGTCGTAAAGTCCA
TAGCACCAAAGCCAGTATGAACCATATCACCATCCTGAATAACTGTGTTTATTAAC
TCTAATGGTGGACAATCACCTGGATTTACTGCAACATTGGTACATGGGGATCCTTTG
CCCCAGTGTTCCCCTAT

#23 AGCCCTATTAAATAAATGTCTAACAAACATTTGTTCCCTTCGTAAATAAAAAAATA
AGCTGTCGCCATATGGTTCTGACACCATTTTAATATAATCTGGATATTTGCAAATAG
ATGTACAAATATCCAGTGGAACTTCACTTTTGTTAGCCTGTAATGTCTTAAAGTCCA
TAGCACCAAAGCCAGTATGAACCATATCACCATCCTGAATAACTGTGTTTATTAAC
TCTAATGGTGGACAATCACCTGGATTTACTGCAACATTGGTACATGGGGATCCTTTG
CCCCAGTGTTCCCCTAT

#24 AGCCCTATTAAATAAATGTCTAACAAACATTTGTTCCCTTCGTAAATAAAAAAATA

AGCTGTCGCCATATGGTTCTGACACCATTTTAATATAATCTGGATATTTGCAAATAG
ATGTACAAATATCCAGTGGAACTTCACTTTTGTTAGCCTGTAATG**GCT**TAAAGTCC
ATAGCACCAAAGCCAGTATGAACCATATCACCATCCTGAATAACTGTGTTTATTAA
CTCTAATGGTGGACAATCACCTGGATTTACTGCAACATTGGTACATGGGGATCCTTT
GCCCCAGTGTTCCCCTAT

#25 AGCCCTATTAAATAAATGTCTAACAAACATTTGTTCCCTTCGTAAATAAAAAAATA
AGCTGTCGCCATATGGTTCTGACACCATTTTAATATAATCTGGATATTTGCAAATAG
ATGTACAAATATCCAGTGGAACTTCACTTTTGTTAGCCTGTAA**G**GTAGTAAAGTCC
ATAGCACCAAAGCCAGTATGAACCATATCACCATCCTGAATAACTGTGTTTATTAA
CTCTAATGGTGGACAATCACCTGGATTTACTGCAACATTGGTACATGGGGATCCTTT
GCCCCAGTGTTCCCCTAT

#26 AGCCCTATTAAATAAATGTCTAACAAACATTTGTTCCCTTCGTAAATAAAAAAATA
AGCTGTCGCCATATGGTTCTGACACCATTTTAATATAATCTGGATATTTGCAAATAG
ATGTACAAATATCCAGTGGAACTTCACTTTTGTTAGCCTGTAA**GT**TAGTAAAGTCC
ATAGCACCAAAGCCAGTATGAACCATATCACCATCCTGAATAACTGTGTTTATTAA
CTCTAATGGTGGACAATCACCTGGATTTACTGCAACATTGGTACATGGGGATCCTTT
GCCCCAGTGTTCCCCTAT

#27 AGCCCTATTAAATAAATGTCTAACAAACATTTGTTCCCTTCGTAAATAAAAAAATA
AGCTGTCGCCATATGGTTCTGACACCATTTTAATATAATCTGGATATTTGCAAATAG
ATGTACAAATATCCAGTGGAACTTCACTTTTGTTAGCCTGTA**CGT**TAGTAAAGTCC
ATAGCACCAAAGCCAGTATGAACCATATCACCATCCTGAATAACTGTGTTTATTAA
CTCTAATGGTGGACAATCACCTGGATTTACTGCAACATTGGTACATGGGGATCCTTT
GCCCCAGTGTTCCCCTAT

#28 AGCCCTATTAAATAAATGTCTAACAAACATTTGTTCCCTTCGTAAATAAAAAAATA
AGCTGTCGCCATATGGTTCTGACACCATTTTAATATAATCTGGATATTTGCAAATAG
ATGTACAAATATCCAGTGGAACTTCACTTTTGTTAGCCTG**G**AATGTAGTAAAGTCC
ATAGCACCAAAGCCAGTATGAACCATATCACCATCCTGAATAACTGTGTTTATTAA
CTCTAATGGTGGACAATCACCTGGATTTACTGCAACATTGGTACATGGGGATCCTTT
GCCCCAGTGTTCCCCTAT

#29 AGCCCTATTAAATAAATGTCTAACAAACATTTGTTCCCTTCGTAAATAAAAAAATA
AGCTGTCGCCATATGGTTCTGACACCATTTTAATATAATCTGGATATTTGCAAATAG
ATGTACAAATATCCAGTGGAACTTCACTTTTGTTAGCCTG**GC**ATGTAGTAAAGTCC
ATAGCACCAAAGCCAGTATGAACCATATCACCATCCTGAATAACTGTGTTTATTAA
CTCTAATGGTGGACAATCACCTGGATTTACTGCAACATTGGTACATGGGGATCCTTT
GCCCCAGTGTTCCCCTAT

#30 AGCCCTATTAAATAAATGTCTAACAAACATTTGTTCCCTTCGTAAATAAAAAAATA
AGCTGTCGCCATATGGTTCTGACACCATTTTAATATAATCTGGATATTTGCAAATAG
ATGTACAAATATCCAGTGGAACTTCACTTTTGTTAGCCT**TGC**ATGTAGTAAAGTCC
ATAGCACCAAAGCCAGTATGAACCATATCACCATCCTGAATAACTGTGTTTATTAA
CTCTAATGGTGGACAATCACCTGGATTTACTGCAACATTGGTACATGGGGATCCTTT
GCCCCAGTGTTCCCCTAT

#31 AGCCCTATTAAATAAATGTCTAACAAACATTTGTTCCCTTCGTAAATAAAAAAATA
AGCTGTCGCCATATGGTTCTGACACCATTTTAATATAATCTGGATATTTGCAAATAG
ATGTACAAATATCCAGTGGAACTTCACTTTTGTTAGC**A**TGTAATGTAGTAAAGTCCA
TAGCACCAAAGCCAGTATGAACCATATCACCATCCTGAATAACTGTGTTTATTAAC
TCTAATGGTGGACAATCACCTGGATTTACTGCAACATTGGTACATGGGGATCCTTTG
CCCCAGTGTTCCCCTAT

#32 AGCCCTATTAAATAAATGTCTAACAAACATTTGTTCCCTTCGTAAATAAAAAAATA
AGCTGTCGCCATATGGTTCTGACACCATTTTAATATAATCTGGATATTTGCAAATAG
ATGTACAAATATCCAGTGGAACTTCACTTTTGTTAGC**AG**GTAATGTAGTAAAGTCC
ATAGCACCAAAGCCAGTATGAACCATATCACCATCCTGAATAACTGTGTTTATTAA

CTCTAATGGTGGACAATCACCTGGATTTACTGCAACATTGGTACATGGGGATCCTTT
GCCCCAGTGTTCCCCTAT

#33 AGCCCTATTAAATAAATGTCTAACAAACATTTGTTCCCTTCGTAAATAAAAAAATA
AGCTGTCGCCATATGGTTCTGACACCATTTTAATATAATCTGGATATTTGCAAATAG
ATGTACAAATATCCAGTGGAACTTCACTTTTGTTAG**AAG**GTAATGTAGTAAAGTCC
ATAGCACCAAAGCCAGTATGAACCATATCACCATCCTGAATAACTGTGTTTATTAA
CTCTAATGGTGGACAATCACCTGGATTTACTGCAACATTGGTACATGGGGATCCTTT
GCCCCAGTGTTCCCCTAT

#34 AGCCCTATTAAATAAATGTCTAACAAACATTTGTTCCCTTCGTAAATAAAAAAATA
AGCTGTCGCCATATGGTTCTGACACCATTTTAATATAATCTGGATATTTGCAAATAG
ATGTACAAATATCCAGTGGAACTTCACTTTTGTT**C**GCCTGTAATGTAGTAAAGTCCA
TAGCACCAAAGCCAGTATGAACCATATCACCATCCTGAATAACTGTGTTTATTAAC
TCTAATGGTGGACAATCACCTGGATTTACTGCAACATTGGTACATGGGGATCCTTTG
CCCCAGTGTTCCCCTAT

#35 AGCCCTATTAAATAAATGTCTAACAAACATTTGTTCCCTTCGTAAATAAAAAAATA
AGCTGTCGCCATATGGTTCTGACACCATTTTAATATAATCTGGATATTTGCAAATAG
ATGTACAAATATCCAGTGGAACTTCACTTTTGTT**CT**CCTGTAATGTAGTAAAGTCCA
TAGCACCAAAGCCAGTATGAACCATATCACCATCCTGAATAACTGTGTTTATTAAC
TCTAATGGTGGACAATCACCTGGATTTACTGCAACATTGGTACATGGGGATCCTTTG
CCCCAGTGTTCCCCTAT

#36 AGCCCTATTAAATAAATGTCTAACAAACATTTGTTCCCTTCGTAAATAAAAAAATA
AGCTGTCGCCATATGGTTCTGACACCATTTTAATATAATCTGGATATTTGCAAATAG
ATGTACAAATATCCAGTGGAACTTCACTTTTGT**GCT**CCTGTAATGTAGTAAAGTCC
ATAGCACCAAAGCCAGTATGAACCATATCACCATCCTGAATAACTGTGTTTATTAA
CTCTAATGGTGGACAATCACCTGGATTTACTGCAACATTGGTACATGGGGATCCTTT
GCCCCAGTGTTCCCCTAT

#37 AGCCCTATTAAATAAATGTCTAACAAACATTTGTTCCCTTCGTAAATAAAAAAATA
AGCTGTCGCCATATGGTTCTGACACCATTTTAATATAATCTGGATATTTGCAAATAG
ATGTACAAATATCCAGTGGAACTTCACTTTT**T**TTAGCCTGTAATGTAGTAAAGTCCA
TAGCACCAAAGCCAGTATGAACCATATCACCATCCTGAATAACTGTGTTTATTAAC
TCTAATGGTGGACAATCACCTGGATTTACTGCAACATTGGTACATGGGGATCCTTTG
CCCCAGTGTTCCCCTAT

#38 AGCCCTATTAAATAAATGTCTAACAAACATTTGTTCCCTTCGTAAATAAAAAAATA
AGCTGTCGCCATATGGTTCTGACACCATTTTAATATAATCTGGATATTTGCAAATAG
ATGTACAAATATCCAGTGGAACTTCACTTTT**TG**TAGCCTGTAATGTAGTAAAGTCC
ATAGCACCAAAGCCAGTATGAACCATATCACCATCCTGAATAACTGTGTTTATTAA
CTCTAATGGTGGACAATCACCTGGATTTACTGCAACATTGGTACATGGGGATCCTTT
GCCCCAGTGTTCCCCTAT

#39 AGCCCTATTAAATAAATGTCTAACAAACATTTGTTCCCTTCGTAAATAAAAAAATA
AGCTGTCGCCATATGGTTCTGACACCATTTTAATATAATCTGGATATTTGCAAATAG
ATGTACAAATATCCAGTGGAACTTCACTTT**GTG**TAGCCTGTAATGTAGTAAAGTCC
ATAGCACCAAAGCCAGTATGAACCATATCACCATCCTGAATAACTGTGTTTATTAA
CTCTAATGGTGGACAATCACCTGGATTTACTGCAACATTGGTACATGGGGATCCTTT
GCCCCAGTGTTCCCCTAT

#40 AGCCCTATTAAATAAATGTCTAACAAACATTTGTTCCCTTCGTAAATAAAAAAATA
AGCTGTCGCCATATGGTTCTGACACCATTTTAATATAATCTGGATATTTGCAAATAG
ATGTACAAATATCCAGTGGAACTTCACT**G**TTGTTAGCCTGTAATGTAGTAAAGTCC
ATAGCACCAAAGCCAGTATGAACCATATCACCATCCTGAATAACTGTGTTTATTAA
CTCTAATGGTGGACAATCACCTGGATTTACTGCAACATTGGTACATGGGGATCCTTT
GCCCCAGTGTTCCCCTAT

#41 AGCCCTATTAAATAAATGTCTAACAAACATTTGTTCCCTTCGTAAATAAAAAAATA

AGCTGTCGCCATATGGTTCTGACACCATTTTAATATAATCTGGATATTTGCAAATAG
ATGTACAAATATCCAGTGGAACTTCACT**GG**TGTTAGCCTGTAATGTAGTAAAGTCC
ATAGCACCAAAGCCAGTATGAACCATATCACCATCCTGAATAACTGTGTTTATTAA
CTCTAATGGTGGACAATCACCTGGATTTACTGCAACATTGGTACATGGGGATCCTTT
GCCCCAGTGTTCCCCTAT

#42 AGCCCTATTAAATAAATGTCTAACAAACATTTGTTCCCTTCGTAAATAAAAAAATA
AGCTGTCGCCATATGGTTCTGACACCATTTTAATATAATCTGGATATTTGCAAATAG
ATGTACAAATATCCAGTGGAACTTCAC**GGG**TGTTAGCCTGTAATGTAGTAAAGTCC
ATAGCACCAAAGCCAGTATGAACCATATCACCATCCTGAATAACTGTGTTTATTAA
CTCTAATGGTGGACAATCACCTGGATTTACTGCAACATTGGTACATGGGGATCCTTT
GCCCCAGTGTTCCCCTAT

#43 AGCCCTATTAAATAAATGTCTAACAAACATTTGTTCCCTTCGTAAATAAAAAAATA
AGCTGTCGCCATATGGTTCTGACACCATTTTAATATAATCTGGATATTTGCAAATAG
ATGTACAAATATCCAGTGGAACTTCACTTTTGTTAGCCTGTAATGT**G**GTAAAGTCCA
TAGCACCAAAGCCAGTATGAACCATATCACCATCCTGAATAACTGTGTTTATTAAC
TCTAATGGTGGACAATCACCTGGATTTACTGCAACATTGGTACATGGGGATCCTTTG
CCCCAGTGTTCCCCTAT

#44 AGCCCTATTAAATAAATGTCTAACAAACATTTGTTCCCTTCGTAAATAAAAAAATA
AGCTGTCGCCATATGGTTCTGACACCATTTTAATATAATCTGGATATTTGCAAATAG
ATGTACAAATATCCAGTGGAACTTCACTTTTGTTAGCCTGTAATGT**GA**TAAAGTCCA
TAGCACCAAAGCCAGTATGAACCATATCACCATCCTGAATAACTGTGTTTATTAAC
TCTAATGGTGGACAATCACCTGGATTTACTGCAACATTGGTACATGGGGATCCTTTG
CCCCAGTGTTCCCCTAT

#45 AGCCCTATTAAATAAATGTCTAACAAACATTTGTTCCCTTCGTAAATAAAAAAATA
AGCTGTCGCCATATGGTTCTGACACCATTTTAATATAATCTGGATATTTGCAAATAG
ATGTACAAATATCCAGTGGAACTTCACTTTTGTTAGCCTGTAATG**CGA**TAAAGTCC
ATAGCACCAAAGCCAGTATGAACCATATCACCATCCTGAATAACTGTGTTTATTAA
CTCTAATGGTGGACAATCACCTGGATTTACTGCAACATTGGTACATGGGGATCCTTT
GCCCCAGTGTTCCCCTAT

#46 AGCCCTATTAAATAAATGTCTAACAAACATTTGTTCCCTTCGTAAATAAAAAAATA
AGCTGTCGCCATATGGTTCTGACACCATTTTAATATAATCTGGATATTTGCAAATAG
ATGTACAAATATCCAGTGGAACTTCACTTTTGTTAGCCTGTAA**C**GTAGTAAAGTCC
ATAGCACCAAAGCCAGTATGAACCATATCACCATCCTGAATAACTGTGTTTATTAA
CTCTAATGGTGGACAATCACCTGGATTTACTGCAACATTGGTACATGGGGATCCTTT
GCCCCAGTGTTCCCCTAT

#47 AGCCCTATTAAATAAATGTCTAACAAACATTTGTTCCCTTCGTAAATAAAAAAATA
AGCTGTCGCCATATGGTTCTGACACCATTTTAATATAATCTGGATATTTGCAAATAG
ATGTACAAATATCCAGTGGAACTTCACTTTTGTTAGCCTGTAA**CA**TAGTAAAGTCC
ATAGCACCAAAGCCAGTATGAACCATATCACCATCCTGAATAACTGTGTTTATTAA
CTCTAATGGTGGACAATCACCTGGATTTACTGCAACATTGGTACATGGGGATCCTTT
GCCCCAGTGTTCCCCTAT

#48 AGCCCTATTAAATAAATGTCTAACAAACATTTGTTCCCTTCGTAAATAAAAAAATA
AGCTGTCGCCATATGGTTCTGACACCATTTTAATATAATCTGGATATTTGCAAATAG
ATGTACAAATATCCAGTGGAACTTCACTTTTGTTAGCCTGTA**GCA**TAGTAAAGTCC
ATAGCACCAAAGCCAGTATGAACCATATCACCATCCTGAATAACTGTGTTTATTAA
CTCTAATGGTGGACAATCACCTGGATTTACTGCAACATTGGTACATGGGGATCCTTT
GCCCCAGTGTTCCCCTAT

#49 AGCCCTATTAAATAAATGTCTAACAAACATTTGTTCCCTTCGTAAATAAAAAAATA
AGCTGTCGCCATATGGTTCTGACACCATTTTAATATAATCTGGATATTTGCAAATAG
ATGTACAAATATCCAGTGGAACTTCACTTTTGTTAGCCTG**C**AATGTAGTAAAGTCC
ATAGCACCAAAGCCAGTATGAACCATATCACCATCCTGAATAACTGTGTTTATTAA

CTCTAATGGTGGACAATCACCTGGATTTACTGCAACATTGGTACATGGGGATCCTTT
GCCCCAGTGTTCCCCTAT

#50 AGCCCTATTAAATAAATGTCTAACAAACATTTGTTCCCTTCGTAAATAAAAAAATA
AGCTGTCGCCATATGGTTCTGACACCATTTTAATATAATCTGGATATTTGCAAATAG
ATGTACAAATATCCAGTGGAACTTCACTTTTGTTAGCCTG**CG**ATGTAGTAAAGTCC
ATAGCACCAAAGCCAGTATGAACCATATCACCATCCTGAATAACTGTGTTTATTAA
CTCTAATGGTGGACAATCACCTGGATTTACTGCAACATTGGTACATGGGGATCCTTT
GCCCCAGTGTTCCCCTAT

#51 AGCCCTATTAAATAAATGTCTAACAAACATTTGTTCCCTTCGTAAATAAAAAAATA
AGCTGTCGCCATATGGTTCTGACACCATTTTAATATAATCTGGATATTTGCAAATAG
ATGTACAAATATCCAGTGGAACTTCACTTTTGTTAGCCT**ACG**ATGTAGTAAAGTCC
ATAGCACCAAAGCCAGTATGAACCATATCACCATCCTGAATAACTGTGTTTATTAA
CTCTAATGGTGGACAATCACCTGGATTTACTGCAACATTGGTACATGGGGATCCTTT
GCCCCAGTGTTCCCCTAT

#52 AGCCCTATTAAATAAATGTCTAACAAACATTTGTTCCCTTCGTAAATAAAAAAATA
AGCTGTCGCCATATGGTTCTGACACCATTTTAATATAATCTGGATATTTGCAAATAG
ATGTACAAATATCCAGTGGAACTTCACTTTTGTTAGC**T**TGTAATGTAGTAAAGTCCA
TAGCACCAAAGCCAGTATGAACCATATCACCATCCTGAATAACTGTGTTTATTAAC
TCTAATGGTGGACAATCACCTGGATTTACTGCAACATTGGTACATGGGGATCCTTTG
CCCCAGTGTTCCCCTAT

#53 AGCCCTATTAAATAAATGTCTAACAAACATTTGTTCCCTTCGTAAATAAAAAAATA
AGCTGTCGCCATATGGTTCTGACACCATTTTAATATAATCTGGATATTTGCAAATAG
ATGTACAAATATCCAGTGGAACTTCACTTTTGTTAGC**TC**GTAATGTAGTAAAGTCC
ATAGCACCAAAGCCAGTATGAACCATATCACCATCCTGAATAACTGTGTTTATTAA
CTCTAATGGTGGACAATCACCTGGATTTACTGCAACATTGGTACATGGGGATCCTTT
GCCCCAGTGTTCCCCTAT

#54 AGCCCTATTAAATAAATGTCTAACAAACATTTGTTCCCTTCGTAAATAAAAAAATA
AGCTGTCGCCATATGGTTCTGACACCATTTTAATATAATCTGGATATTTGCAAATAG
ATGTACAAATATCCAGTGGAACTTCACTTTTGTTAG**TTC**GTAATGTAGTAAAGTCC
ATAGCACCAAAGCCAGTATGAACCATATCACCATCCTGAATAACTGTGTTTATTAA
CTCTAATGGTGGACAATCACCTGGATTTACTGCAACATTGGTACATGGGGATCCTTT
GCCCCAGTGTTCCCCTAT

#55 AGCCCTATTAAATAAATGTCTAACAAACATTTGTTCCCTTCGTAAATAAAAAAATA
AGCTGTCGCCATATGGTTCTGACACCATTTTAATATAATCTGGATATTTGCAAATAG
ATGTACAAATATCCAGTGGAACTTCACTTTTGTT**G**GCCTGTAATGTAGTAAAGTCCA
TAGCACCAAAGCCAGTATGAACCATATCACCATCCTGAATAACTGTGTTTATTAAC
TCTAATGGTGGACAATCACCTGGATTTACTGCAACATTGGTACATGGGGATCCTTTG
CCCCAGTGTTCCCCTAT

#56 AGCCCTATTAAATAAATGTCTAACAAACATTTGTTCCCTTCGTAAATAAAAAAATA
AGCTGTCGCCATATGGTTCTGACACCATTTTAATATAATCTGGATATTTGCAAATAG
ATGTACAAATATCCAGTGGAACTTCACTTTTGTT**GA**CCTGTAATGTAGTAAAGTCCA
TAGCACCAAAGCCAGTATGAACCATATCACCATCCTGAATAACTGTGTTTATTAAC
TCTAATGGTGGACAATCACCTGGATTTACTGCAACATTGGTACATGGGGATCCTTTG
CCCCAGTGTTCCCCTAT

#57 AGCCCTATTAAATAAATGTCTAACAAACATTTGTTCCCTTCGTAAATAAAAAAATA
AGCTGTCGCCATATGGTTCTGACACCATTTTAATATAATCTGGATATTTGCAAATAG
ATGTACAAATATCCAGTGGAACTTCACTTTTGT**CGA**CCTGTAATGTAGTAAAGTCC
ATAGCACCAAAGCCAGTATGAACCATATCACCATCCTGAATAACTGTGTTTATTAA
CTCTAATGGTGGACAATCACCTGGATTTACTGCAACATTGGTACATGGGGATCCTTT
GCCCCAGTGTTCCCCTAT

#58 AGCCCTATTAAATAAATGTCTAACAAACATTTGTTCCCTTCGTAAATAAAAAAATA

	AGCTGTCGCCATATGGTTCTGACACCATTTTAATATAATCTGGATATTTGCAAATAG<br>ATGTACAAATATCCAGTGGAACTTCACTTTTATTAGCCTGTAATGTAGTAAAGTCCA<br>TAGCACCAAAGCCAGTATGAACCATATCACCATCCTGAATAACTGTGTTTATTAAC<br>TCTAATGGTGGACAATCACCTGGATTTACTGCAACATTGGTACATGGGGATCCTTTG<br>CCCCAGTGTTCCCCTAT
#59	AGCCCTATTAAATAAATGTCTAACAAACATTTGTTCCCTTCGTAAATAAAAAAATA<br>AGCTGTCGCCATATGGTTCTGACACCATTTTAATATAATCTGGATATTTGCAAATAG<br>ATGTACAAATATCCAGTGGAACTTCACTTTTACTAGCCTGTAATGTAGTAAAGTCC<br>ATAGCACCAAAGCCAGTATGAACCATATCACCATCCTGAATAACTGTGTTTATTAA<br>CTCTAATGGTGGACAATCACCTGGATTTACTGCAACATTGGTACATGGGGATCCTTT<br>GCCCCAGTGTTCCCCTAT
#60	AGCCCTATTAAATAAATGTCTAACAAACATTTGTTCCCTTCGTAAATAAAAAAATA<br>AGCTGTCGCCATATGGTTCTGACACCATTTTAATATAATCTGGATATTTGCAAATAG<br>ATGTACAAATATCCAGTGGAACTTCACTTTCACTAGCCTGTAATGTAGTAAAGTCC<br>ATAGCACCAAAGCCAGTATGAACCATATCACCATCCTGAATAACTGTGTTTATTAA<br>CTCTAATGGTGGACAATCACCTGGATTTACTGCAACATTGGTACATGGGGATCCTTT<br>GCCCCAGTGTTCCCCTAT
#61	AGCCCTATTAAATAAATGTCTAACAAACATTTGTTCCCTTCGTAAATAAAAAAATA<br>AGCTGTCGCCATATGGTTCTGACACCATTTTAATATAATCTGGATATTTGCAAATAG<br>ATGTACAAATATCCAGTGGAACTTCACTCTTGTTAGCCTGTAATGTAGTAAAGTCC<br>ATAGCACCAAAGCCAGTATGAACCATATCACCATCCTGAATAACTGTGTTTATTAA<br>CTCTAATGGTGGACAATCACCTGGATTTACTGCAACATTGGTACATGGGGATCCTTT<br>GCCCCAGTGTTCCCCTAT
#62	AGCCCTATTAAATAAATGTCTAACAAACATTTGTTCCCTTCGTAAATAAAAAAATA<br>AGCTGTCGCCATATGGTTCTGACACCATTTTAATATAATCTGGATATTTGCAAATAG<br>ATGTACAAATATCCAGTGGAACTTCACTCCTGTTAGCCTGTAATGTAGTAAAGTCC<br>ATAGCACCAAAGCCAGTATGAACCATATCACCATCCTGAATAACTGTGTTTATTAA<br>CTCTAATGGTGGACAATCACCTGGATTTACTGCAACATTGGTACATGGGGATCCTTT<br>GCCCCAGTGTTCCCCTAT
#63	AGCCCTATTAAATAAATGTCTAACAAACATTTGTTCCCTTCGTAAATAAAAAAATA<br>AGCTGTCGCCATATGGTTCTGACACCATTTTAATATAATCTGGATATTTGCAAATAG<br>ATGTACAAATATCCAGTGGAACTTCACCCCTGTTAGCCTGTAATGTAGTAAAGTCC<br>ATAGCACCAAAGCCAGTATGAACCATATCACCATCCTGAATAACTGTGTTTATTAA<br>CTCTAATGGTGGACAATCACCTGGATTTACTGCAACATTGGTACATGGGGATCCTTT<br>GCCCCAGTGTTCCCCTAT

* The target region of CRISPR-Cas12a is underlined, and the three rules for nucleotide substitution are highlighted in red, green, and blue, respectively.

Supplementary Table 7. Nucleotide sequences of mismatch profiles, used in the Supplementary Figure 2. The sequences shown are from HPV16 and were randomly selected for illustration purposes.

Name	***Sequence (5'→3')***
crRNA	UAAUUUCUACUAAGUGUAGAUUGUGUCAUCCAAUUUAUUUAA
PM	AAGTCCATAGCACCAAAGCCAGTATGAACCATATCACCATCCTGAATAACTGTGTTTA<br>TTAACTCTAATGGTGGACAATCACCTGGATTTACTGCAACATTGGTACATGGGGATCC<br>TTTGCCCCAGTGTTCCCCTATAGGTGGTTTGCAACCAATTAAACACAATTGTGTTTGT<br>TTGTAATCCATAGATATACATTCTCTATTATCCACACCTGCATTTGCTGCATAAGCACTA<br>GCATTTTCTGTGTCATCCAATTTATTTAATAAAGGATGGCCACTAATGCCCACACCTAA<br>TGGCTGACC
#1	AAGTCCATAGCACCAAAGCCAGTATGAACCATATCACCATCCTGAATAACTGTGTTTA<br>TTAACTCTAATGGTGGACAATCACCTGGATTTACTGCAACATTGGTACATGGGGATCC<br>TTTGCCCCAGTGTTCCCCTATAGGTGGTTTGCAACCAATTAAACACAATTGTGTTTGT<br>TTGTAATCCATAGATATACATTCTCTATTATCCACACCTGCATTTGCTGCATAAGCACTA<br>GCATTTTCTCTGTCATCCAATTTATTTAATAAAGGATGGCCACTAATGCCCACACCTA<br>ATGGCTGACC
#2	AAGTCCATAGCACCAAAGCCAGTATGAACCATATCACCATCCTGAATAACTGTGTTTA<br>TTAACTCTAATGGTGGACAATCACCTGGATTTACTGCAACATTGGTACATGGGGATCC<br>TTTGCCCCAGTGTTCCCCTATAGGTGGTTTGCAACCAATTAAACACAATTGTGTTTGT<br>TTGTAATCCATAGATATACATTCTCTATTATCCACACCTGCATTTGCTGCATAAGCACTA<br>GCATTTTCACTGTCATCCAATTTATTTAATAAAGGATGGCCACTAATGCCCACACCTA<br>ATGGCTGACC
#3	AAGTCCATAGCACCAAAGCCAGTATGAACCATATCACCATCCTGAATAACTGTGTTTA<br>TTAACTCTAATGGTGGACAATCACCTGGATTTACTGCAACATTGGTACATGGGGATCC<br>TTTGCCCCAGTGTTCCCCTATAGGTGGTTTGCAACCAATTAAACACAATTGTGTTTGT<br>TTGTAATCCATAGATATACATTCTCTATTATCCACACCTGCATTTGCTGCATAAGCACTA<br>GCATTTTCACAGTCATCCAATTTATTTAATAAAGGATGGCCACTAATGCCCACACCTA<br>ATGGCTGACC
#4	AAGTCCATAGCACCAAAGCCAGTATGAACCATATCACCATCCTGAATAACTGTGTTTA<br>TTAACTCTAATGGTGGACAATCACCTGGATTTACTGCAACATTGGTACATGGGGATCC<br>TTTGCCCCAGTGTTCCCCTATAGGTGGTTTGCAACCAATTAAACACAATTGTGTTTGT<br>TTGTAATCCATAGATATACATTCTCTATTATCCACACCTGCATTTGCTGCATAAGCACTA<br>GCATTTTCTGTGACATCCAATTTATTTAATAAAGGATGGCCACTAATGCCCACACCTA<br>ATGGCTGACC
#5	AAGTCCATAGCACCAAAGCCAGTATGAACCATATCACCATCCTGAATAACTGTGTTTA<br>TTAACTCTAATGGTGGACAATCACCTGGATTTACTGCAACATTGGTACATGGGGATCC<br>TTTGCCCCAGTGTTCCCCTATAGGTGGTTTGCAACCAATTAAACACAATTGTGTTTGT<br>TTGTAATCCATAGATATACATTCTCTATTATCCACACCTGCATTTGCTGCATAAGCACTA<br>GCATTTTCTGTCACATCCAATTTATTTAATAAAGGATGGCCACTAATGCCCACACCTA<br>ATGGCTGACC
#6	AAGTCCATAGCACCAAAGCCAGTATGAACCATATCACCATCCTGAATAACTGTGTTTA<br>TTAACTCTAATGGTGGACAATCACCTGGATTTACTGCAACATTGGTACATGGGGATCC<br>TTTGCCCCAGTGTTCCCCTATAGGTGGTTTGCAACCAATTAAACACAATTGTGTTTGT<br>TTGTAATCCATAGATATACATTCTCTATTATCCACACCTGCATTTGCTGCATAAGCACTA<br>GCATTTTCTGTCAGATCCAATTTATTTAATAAAGGATGGCCACTAATGCCCACACCTA<br>ATGGCTGACC
#7	AAGTCCATAGCACCAAAGCCAGTATGAACCATATCACCATCCTGAATAACTGTGTTTA<br>TTAACTCTAATGGTGGACAATCACCTGGATTTACTGCAACATTGGTACATGGGGATCC<br>TTTGCCCCAGTGTTCCCCTATAGGTGGTTTGCAACCAATTAAACACAATTGTGTTTGT<br>TTGTAATCCATAGATATACATTCTCTATTATCCACACCTGCATTTGCTGCATAAGCACTA

GCATTTTCTGTGTCA**A**CCAATTTATTTAATAAAGGATGGCCACTAATGCCCACACCTA
ATGGCTGACC

#8 AAGTCCATAGCACCAAAGCCAGTATGAACCATATCACCATCCTGAATAACTGTGTTTA
TTAACTCTAATGGTGGACAATCACCTGGATTTACTGCAACATTGGTACATGGGGATCC
TTTGCCCCAGTGTTCCCCTATAGGTGGTTTGCAACCAATTAAACACAATTGTGTTTGT
TTGTAATCCATAGATATACATTCTCTATTATCCACACCTGCATTTGCTGCATAAGCACTA
GCATTTTCTGTGTC**TA**CCAATTTATTTAATAAAGGATGGCCACTAATGCCCACACCTA
ATGGCTGACC

#9 AAGTCCATAGCACCAAAGCCAGTATGAACCATATCACCATCCTGAATAACTGTGTTTA
TTAACTCTAATGGTGGACAATCACCTGGATTTACTGCAACATTGGTACATGGGGATCC
TTTGCCCCAGTGTTCCCCTATAGGTGGTTTGCAACCAATTAAACACAATTGTGTTTGT
TTGTAATCCATAGATATACATTCTCTATTATCCACACCTGCATTTGCTGCATAAGCACTA
GCATTTTCTGTGTC**TAG**CAATTTATTTAATAAAGGATGGCCACTAATGCCCACACCTA
ATGGCTGACC

#10 AAGTCCATAGCACCAAAGCCAGTATGAACCATATCACCATCCTGAATAACTGTGTTTA
TTAACTCTAATGGTGGACAATCACCTGGATTTACTGCAACATTGGTACATGGGGATCC
TTTGCCCCAGTGTTCCCCTATAGGTGGTTTGCAACCAATTAAACACAATTGTGTTTGT
TTGTAATCCATAGATATACATTCTCTATTATCCACACCTGCATTTGCTGCATAAGCACTA
GCATTTTCTGTGTCATCC**T**ATTTATTTAATAAAGGATGGCCACTAATGCCCACACCTA
ATGGCTGACC

#11 AAGTCCATAGCACCAAAGCCAGTATGAACCATATCACCATCCTGAATAACTGTGTTTA
TTAACTCTAATGGTGGACAATCACCTGGATTTACTGCAACATTGGTACATGGGGATCC
TTTGCCCCAGTGTTCCCCTATAGGTGGTTTGCAACCAATTAAACACAATTGTGTTTGT
TTGTAATCCATAGATATACATTCTCTATTATCCACACCTGCATTTGCTGCATAAGCACTA
GCATTTTCTGTGTCATC**GT**ATTTATTTAATAAAGGATGGCCACTAATGCCCACACCTA
ATGGCTGACC

#12 AAGTCCATAGCACCAAAGCCAGTATGAACCATATCACCATCCTGAATAACTGTGTTTA
TTAACTCTAATGGTGGACAATCACCTGGATTTACTGCAACATTGGTACATGGGGATCC
TTTGCCCCAGTGTTCCCCTATAGGTGGTTTGCAACCAATTAAACACAATTGTGTTTGT
TTGTAATCCATAGATATACATTCTCTATTATCCACACCTGCATTTGCTGCATAAGCACTA
GCATTTTCTGTGTCATC**GTT**TTTATTTAATAAAGGATGGCCACTAATGCCCACACCTA
ATGGCTGACC

#13 AAGTCCATAGCACCAAAGCCAGTATGAACCATATCACCATCCTGAATAACTGTGTTTA
TTAACTCTAATGGTGGACAATCACCTGGATTTACTGCAACATTGGTACATGGGGATCC
TTTGCCCCAGTGTTCCCCTATAGGTGGTTTGCAACCAATTAAACACAATTGTGTTTGT
TTGTAATCCATAGATATACATTCTCTATTATCCACACCTGCATTTGCTGCATAAGCACTA
GCATTTTCTGTGTCATCCAAT**A**TATTTAATAAAGGATGGCCACTAATGCCCACACCTA
ATGGCTGACC

#14 AAGTCCATAGCACCAAAGCCAGTATGAACCATATCACCATCCTGAATAACTGTGTTTA
TTAACTCTAATGGTGGACAATCACCTGGATTTACTGCAACATTGGTACATGGGGATCC
TTTGCCCCAGTGTTCCCCTATAGGTGGTTTGCAACCAATTAAACACAATTGTGTTTGT
TTGTAATCCATAGATATACATTCTCTATTATCCACACCTGCATTTGCTGCATAAGCACTA
GCATTTTCTGTGTCATCCAA**AA**TATTTAATAAAGGATGGCCACTAATGCCCACACCTA
ATGGCTGACC

#15 AAGTCCATAGCACCAAAGCCAGTATGAACCATATCACCATCCTGAATAACTGTGTTTA
TTAACTCTAATGGTGGACAATCACCTGGATTTACTGCAACATTGGTACATGGGGATCC
TTTGCCCCAGTGTTCCCCTATAGGTGGTTTGCAACCAATTAAACACAATTGTGTTTGT
TTGTAATCCATAGATATACATTCTCTATTATCCACACCTGCATTTGCTGCATAAGCACTA
GCATTTTCTGTGTCATCCAA**AAA**ATTTAATAAAGGATGGCCACTAATGCCCACACCT
AATGGCTGACC

#16 AAGTCCATAGCACCAAAGCCAGTATGAACCATATCACCATCCTGAATAACTGTGTTTA

TTAACTCTAATGGTGGACAATCACCTGGATTTACTGCAACATTGGTACATGGGGATCC
TTTGCCCCAGTGTTCCCCTATAGGTGGTTTGCAACCAATTAAACACAATTGTGTTTGT
TTGTAATCCATAGATATACATTCTCTATTATCCACACCTGCATTTGCTGCATAAGCACTA
GCATTTTCTGTGTCATCCAATTTA**A**TTAATAAAGGATGGCCACTAATGCCCACACCTA
ATGGCTGACC

#17 AAGTCCATAGCACCAAAGCCAGTATGAACCATATCACCATCCTGAATAACTGTGTTTA
TTAACTCTAATGGTGGACAATCACCTGGATTTACTGCAACATTGGTACATGGGGATCC
TTTGCCCCAGTGTTCCCCTATAGGTGGTTTGCAACCAATTAAACACAATTGTGTTTGT
TTGTAATCCATAGATATACATTCTCTATTATCCACACCTGCATTTGCTGCATAAGCACTA
GCATTTTCTGTGTCATCCAATTT**TA**TTAATAAAGGATGGCCACTAATGCCCACACCTA
ATGGCTGACC

#18 AAGTCCATAGCACCAAAGCCAGTATGAACCATATCACCATCCTGAATAACTGTGTTTA
TTAACTCTAATGGTGGACAATCACCTGGATTTACTGCAACATTGGTACATGGGGATCC
TTTGCCCCAGTGTTCCCCTATAGGTGGTTTGCAACCAATTAAACACAATTGTGTTTGT
TTGTAATCCATAGATATACATTCTCTATTATCCACACCTGCATTTGCTGCATAAGCACTA
GCATTTTCTGTGTCATCCAATTT**TAA**TAATAAAGGATGGCCACTAATGCCCACACCTA
ATGGCTGACC

#19 AAGTCCATAGCACCAAAGCCAGTATGAACCATATCACCATCCTGAATAACTGTGTTTA
TTAACTCTAATGGTGGACAATCACCTGGATTTACTGCAACATTGGTACATGGGGATCC
TTTGCCCCAGTGTTCCCCTATAGGTGGTTTGCAACCAATTAAACACAATTGTGTTTGT
TTGTAATCCATAGATATACATTCTCTATTATCCACACCTGCATTTGCTGCATAAGCACTA
GCATTTTCTGTGTCATCCAATTTATTT**T**ATAAAGGATGGCCACTAATGCCCACACCTA
ATGGCTGACC

#20 AAGTCCATAGCACCAAAGCCAGTATGAACCATATCACCATCCTGAATAACTGTGTTTA
TTAACTCTAATGGTGGACAATCACCTGGATTTACTGCAACATTGGTACATGGGGATCC
TTTGCCCCAGTGTTCCCCTATAGGTGGTTTGCAACCAATTAAACACAATTGTGTTTGT
TTGTAATCCATAGATATACATTCTCTATTATCCACACCTGCATTTGCTGCATAAGCACTA
GCATTTTCTGTGTCATCCAATTTATT**AT**AATAAAGGATGGCCACTAATGCCCACACCTA
ATGGCTGACC

#21 AAGTCCATAGCACCAAAGCCAGTATGAACCATATCACCATCCTGAATAACTGTGTTTA
TTAACTCTAATGGTGGACAATCACCTGGATTTACTGCAACATTGGTACATGGGGATCC
TTTGCCCCAGTGTTCCCCTATAGGTGGTTTGCAACCAATTAAACACAATTGTGTTTGT
TTGTAATCCATAGATATACATTCTCTATTATCCACACCTGCATTTGCTGCATAAGCACTA
GCATTTTCTGTGTCATCCAATTTATT**ATT**TAAAGGATGGCCACTAATGCCCACACCTA
ATGGCTGACC

#22 AAGTCCATAGCACCAAAGCCAGTATGAACCATATCACCATCCTGAATAACTGTGTTTA
TTAACTCTAATGGTGGACAATCACCTGGATTTACTGCAACATTGGTACATGGGGATCC
TTTGCCCCAGTGTTCCCCTATAGGTGGTTTGCAACCAATTAAACACAATTGTGTTTGT
TTGTAATCCATAGATATACATTCTCTATTATCCACACCTGCATTTGCTGCATAAGCACTA
GCATTTTCT**T**TGTCATCCAATTTATTTAATAAAGGATGGCCACTAATGCCCACACCTA
ATGGCTGACC

#23 AAGTCCATAGCACCAAAGCCAGTATGAACCATATCACCATCCTGAATAACTGTGTTTA
TTAACTCTAATGGTGGACAATCACCTGGATTTACTGCAACATTGGTACATGGGGATCC
TTTGCCCCAGTGTTCCCCTATAGGTGGTTTGCAACCAATTAAACACAATTGTGTTTGT
TTGTAATCCATAGATATACATTCTCTATTATCCACACCTGCATTTGCTGCATAAGCACTA
GCATTTTC**GT**TGTCATCCAATTTATTTAATAAAGGATGGCCACTAATGCCCACACCTA
ATGGCTGACC

#24 AAGTCCATAGCACCAAAGCCAGTATGAACCATATCACCATCCTGAATAACTGTGTTTA
TTAACTCTAATGGTGGACAATCACCTGGATTTACTGCAACATTGGTACATGGGGATCC
TTTGCCCCAGTGTTCCCCTATAGGTGGTTTGCAACCAATTAAACACAATTGTGTTTGT
TTGTAATCCATAGATATACATTCTCTATTATCCACACCTGCATTTGCTGCATAAGCACTA

GCATTTTC<u>**GTG**GTCATCCAATTTATTTAA</u>TAAAGGATGGCCACTAATGCCCACACCTA
ATGGCTGACC

#25 AAGTCCATAGCACCAAAGCCAGTATGAACCATATCACCATCCTGAATAACTGTGTTTA
TTAACTCTAATGGTGGACAATCACCTGGATTTACTGCAACATTGGTACATGGGGATCC
TTTGCCCCAGTGTTCCCCTATAGGTGGTTTGCAACCAATTAAACACAATTGTGTTTGT
TTGTAATCCATAGATATACATTCTCTATTATCCACACCTGCATTTGCTGCATAAGCACTA
GCATTTTC<u>TGTG**G**CATCCAATTTATTTAA</u>TAAAGGATGGCCACTAATGCCCACACCTA
ATGGCTGACC

#26 AAGTCCATAGCACCAAAGCCAGTATGAACCATATCACCATCCTGAATAACTGTGTTTA
TTAACTCTAATGGTGGACAATCACCTGGATTTACTGCAACATTGGTACATGGGGATCC
TTTGCCCCAGTGTTCCCCTATAGGTGGTTTGCAACCAATTAAACACAATTGTGTTTGT
TTGTAATCCATAGATATACATTCTCTATTATCCACACCTGCATTTGCTGCATAAGCACTA
GCATTTTC<u>TGT**TG**CATCCAATTTATTTAA</u>TAAAGGATGGCCACTAATGCCCACACCTA
ATGGCTGACC

#27 AAGTCCATAGCACCAAAGCCAGTATGAACCATATCACCATCCTGAATAACTGTGTTTA
TTAACTCTAATGGTGGACAATCACCTGGATTTACTGCAACATTGGTACATGGGGATCC
TTTGCCCCAGTGTTCCCCTATAGGTGGTTTGCAACCAATTAAACACAATTGTGTTTGT
TTGTAATCCATAGATATACATTCTCTATTATCCACACCTGCATTTGCTGCATAAGCACTA
GCATTTTC<u>TGT**TGA**ATCCAATTTATTTAA</u>TAAAGGATGGCCACTAATGCCCACACCTA
ATGGCTGACC

#28 AAGTCCATAGCACCAAAGCCAGTATGAACCATATCACCATCCTGAATAACTGTGTTTA
TTAACTCTAATGGTGGACAATCACCTGGATTTACTGCAACATTGGTACATGGGGATCC
TTTGCCCCAGTGTTCCCCTATAGGTGGTTTGCAACCAATTAAACACAATTGTGTTTGT
TTGTAATCCATAGATATACATTCTCTATTATCCACACCTGCATTTGCTGCATAAGCACTA
GCATTTTC<u>TGTGTCA**G**CCAATTTATTTAA</u>TAAAGGATGGCCACTAATGCCCACACCTA
ATGGCTGACC

#29 AAGTCCATAGCACCAAAGCCAGTATGAACCATATCACCATCCTGAATAACTGTGTTTA
TTAACTCTAATGGTGGACAATCACCTGGATTTACTGCAACATTGGTACATGGGGATCC
TTTGCCCCAGTGTTCCCCTATAGGTGGTTTGCAACCAATTAAACACAATTGTGTTTGT
TTGTAATCCATAGATATACATTCTCTATTATCCACACCTGCATTTGCTGCATAAGCACTA
GCATTTTC<u>TGTGTC**CG**CCAATTTATTTAA</u>TAAAGGATGGCCACTAATGCCCACACCTA
ATGGCTGACC

#30 AAGTCCATAGCACCAAAGCCAGTATGAACCATATCACCATCCTGAATAACTGTGTTTA
TTAACTCTAATGGTGGACAATCACCTGGATTTACTGCAACATTGGTACATGGGGATCC
TTTGCCCCAGTGTTCCCCTATAGGTGGTTTGCAACCAATTAAACACAATTGTGTTTGT
TTGTAATCCATAGATATACATTCTCTATTATCCACACCTGCATTTGCTGCATAAGCACTA
GCATTTTC<u>TGTGTC**CGA**CAATTTATTTAA</u>TAAAGGATGGCCACTAATGCCCACACCTA
ATGGCTGACC

#31 AAGTCCATAGCACCAAAGCCAGTATGAACCATATCACCATCCTGAATAACTGTGTTTA
TTAACTCTAATGGTGGACAATCACCTGGATTTACTGCAACATTGGTACATGGGGATCC
TTTGCCCCAGTGTTCCCCTATAGGTGGTTTGCAACCAATTAAACACAATTGTGTTTGT
TTGTAATCCATAGATATACATTCTCTATTATCCACACCTGCATTTGCTGCATAAGCACTA
GCATTTTC<u>TGTGTCATCC**C**ATTTATTTAA</u>TAAAGGATGGCCACTAATGCCCACACCTA
ATGGCTGACC

#32 AAGTCCATAGCACCAAAGCCAGTATGAACCATATCACCATCCTGAATAACTGTGTTTA
TTAACTCTAATGGTGGACAATCACCTGGATTTACTGCAACATTGGTACATGGGGATCC
TTTGCCCCAGTGTTCCCCTATAGGTGGTTTGCAACCAATTAAACACAATTGTGTTTGT
TTGTAATCCATAGATATACATTCTCTATTATCCACACCTGCATTTGCTGCATAAGCACTA
GCATTTTC<u>TGTGTCATC**AC**ATTTATTTAA</u>TAAAGGATGGCCACTAATGCCCACACCTA
ATGGCTGACC

#33 AAGTCCATAGCACCAAAGCCAGTATGAACCATATCACCATCCTGAATAACTGTGTTTA

TTAACTCTAATGGTGGACAATCACCTGGATTTACTGCAACATTGGTACATGGGGATCC
TTTGCCCCAGTGTTCCCCTATAGGTGGTTTGCAACCAATTAAACACAATTGTGTTTGT
TTGTAATCCATAGATATACATTCTCTATTATCCACACCTGCATTTGCTGCATAAGCACTA
GCATTTTCTGTGTCATC**ACC**TTTATTTAATAAAGGATGGCCACTAATGCCCACACCTA
ATGGCTGACC

#34 AAGTCCATAGCACCAAAGCCAGTATGAACCATATCACCATCCTGAATAACTGTGTTTA
TTAACTCTAATGGTGGACAATCACCTGGATTTACTGCAACATTGGTACATGGGGATCC
TTTGCCCCAGTGTTCCCCTATAGGTGGTTTGCAACCAATTAAACACAATTGTGTTTGT
TTGTAATCCATAGATATACATTCTCTATTATCCACACCTGCATTTGCTGCATAAGCACTA
GCATTTTCTGTGTCATCCAAT**G**TATTTAATAAAGGATGGCCACTAATGCCCACACCTA
ATGGCTGACC

#35 AAGTCCATAGCACCAAAGCCAGTATGAACCATATCACCATCCTGAATAACTGTGTTTA
TTAACTCTAATGGTGGACAATCACCTGGATTTACTGCAACATTGGTACATGGGGATCC
TTTGCCCCAGTGTTCCCCTATAGGTGGTTTGCAACCAATTAAACACAATTGTGTTTGT
TTGTAATCCATAGATATACATTCTCTATTATCCACACCTGCATTTGCTGCATAAGCACTA
GCATTTTCTGTGTCATCCAA**GG**TATTTAATAAAGGATGGCCACTAATGCCCACACCT
AATGGCTGACC

#36 AAGTCCATAGCACCAAAGCCAGTATGAACCATATCACCATCCTGAATAACTGTGTTTA
TTAACTCTAATGGTGGACAATCACCTGGATTTACTGCAACATTGGTACATGGGGATCC
TTTGCCCCAGTGTTCCCCTATAGGTGGTTTGCAACCAATTAAACACAATTGTGTTTGT
TTGTAATCCATAGATATACATTCTCTATTATCCACACCTGCATTTGCTGCATAAGCACTA
GCATTTTCTGTGTCATCCAA**GGG**ATTTAATAAAGGATGGCCACTAATGCCCACACCT
AATGGCTGACC

#37 AAGTCCATAGCACCAAAGCCAGTATGAACCATATCACCATCCTGAATAACTGTGTTTA
TTAACTCTAATGGTGGACAATCACCTGGATTTACTGCAACATTGGTACATGGGGATCC
TTTGCCCCAGTGTTCCCCTATAGGTGGTTTGCAACCAATTAAACACAATTGTGTTTGT
TTGTAATCCATAGATATACATTCTCTATTATCCACACCTGCATTTGCTGCATAAGCACTA
GCATTTTCTGTGTCATCCAATTTA**G**TTAATAAAGGATGGCCACTAATGCCCACACCTA
ATGGCTGACC

#38 AAGTCCATAGCACCAAAGCCAGTATGAACCATATCACCATCCTGAATAACTGTGTTTA
TTAACTCTAATGGTGGACAATCACCTGGATTTACTGCAACATTGGTACATGGGGATCC
TTTGCCCCAGTGTTCCCCTATAGGTGGTTTGCAACCAATTAAACACAATTGTGTTTGT
TTGTAATCCATAGATATACATTCTCTATTATCCACACCTGCATTTGCTGCATAAGCACTA
GCATTTTCTGTGTCATCCAATTT**CG**TTAATAAAGGATGGCCACTAATGCCCACACCTA
ATGGCTGACC

#39 AAGTCCATAGCACCAAAGCCAGTATGAACCATATCACCATCCTGAATAACTGTGTTTA
TTAACTCTAATGGTGGACAATCACCTGGATTTACTGCAACATTGGTACATGGGGATCC
TTTGCCCCAGTGTTCCCCTATAGGTGGTTTGCAACCAATTAAACACAATTGTGTTTGT
TTGTAATCCATAGATATACATTCTCTATTATCCACACCTGCATTTGCTGCATAAGCACTA
GCATTTTCTGTGTCATCCAATTT**CGG**TAATAAAGGATGGCCACTAATGCCCACACCT
AATGGCTGACC

#40 AAGTCCATAGCACCAAAGCCAGTATGAACCATATCACCATCCTGAATAACTGTGTTTA
TTAACTCTAATGGTGGACAATCACCTGGATTTACTGCAACATTGGTACATGGGGATCC
TTTGCCCCAGTGTTCCCCTATAGGTGGTTTGCAACCAATTAAACACAATTGTGTTTGT
TTGTAATCCATAGATATACATTCTCTATTATCCACACCTGCATTTGCTGCATAAGCACTA
GCATTTTCTGTGTCATCCAATTTATTT**C**ATAAAGGATGGCCACTAATGCCCACACCTA
ATGGCTGACC

#41 AAGTCCATAGCACCAAAGCCAGTATGAACCATATCACCATCCTGAATAACTGTGTTTA
TTAACTCTAATGGTGGACAATCACCTGGATTTACTGCAACATTGGTACATGGGGATCC
TTTGCCCCAGTGTTCCCCTATAGGTGGTTTGCAACCAATTAAACACAATTGTGTTTGT
TTGTAATCCATAGATATACATTCTCTATTATCCACACCTGCATTTGCTGCATAAGCACTA

GCATTTTCTGTGTCATCCAATTTATT**GC**ATAAAGGATGGCCACTAATGCCCACACCTA
ATGGCTGACC

#42 AAGTCCATAGCACCAAAGCCAGTATGAACCATATCACCATCCTGAATAACTGTGTTTA
TTAACTCTAATGGTGGACAATCACCTGGATTTACTGCAACATTGGTACATGGGGATCC
TTTGCCCCAGTGTTCCCCTATAGGTGGTTTGCAACCAATTAAACACAATTGTGTTTGT
TTGTAATCCATAGATATACATTCTCTATTATCCACACCTGCATTTGCTGCATAAGCACTA
GCATTTTCTGTGTCATCCAATTTATT**GCC**TAAAGGATGGCCACTAATGCCCACACCTA
ATGGCTGACC

#43 AAGTCCATAGCACCAAAGCCAGTATGAACCATATCACCATCCTGAATAACTGTGTTTA
TTAACTCTAATGGTGGACAATCACCTGGATTTACTGCAACATTGGTACATGGGGATCC
TTTGCCCCAGTGTTCCCCTATAGGTGGTTTGCAACCAATTAAACACAATTGTGTTTGT
TTGTAATCCATAGATATACATTCTCTATTATCCACACCTGCATTTGCTGCATAAGCACTA
GCATTTTCT**A**TGTCATCCAATTTATTTAATAAAGGATGGCCACTAATGCCCACACCTA
ATGGCTGACC

#44 AAGTCCATAGCACCAAAGCCAGTATGAACCATATCACCATCCTGAATAACTGTGTTTA
TTAACTCTAATGGTGGACAATCACCTGGATTTACTGCAACATTGGTACATGGGGATCC
TTTGCCCCAGTGTTCCCCTATAGGTGGTTTGCAACCAATTAAACACAATTGTGTTTGT
TTGTAATCCATAGATATACATTCTCTATTATCCACACCTGCATTTGCTGCATAAGCACTA
GCATTTTC**CA**TGTCATCCAATTTATTTAATAAAGGATGGCCACTAATGCCCACACCTA
ATGGCTGACC

#45 AAGTCCATAGCACCAAAGCCAGTATGAACCATATCACCATCCTGAATAACTGTGTTTA
TTAACTCTAATGGTGGACAATCACCTGGATTTACTGCAACATTGGTACATGGGGATCC
TTTGCCCCAGTGTTCCCCTATAGGTGGTTTGCAACCAATTAAACACAATTGTGTTTGT
TTGTAATCCATAGATATACATTCTCTATTATCCACACCTGCATTTGCTGCATAAGCACTA
GCATTTTC**CAC**GTCATCCAATTTATTTAATAAAGGATGGCCACTAATGCCCACACCTA
ATGGCTGACC

#46 AAGTCCATAGCACCAAAGCCAGTATGAACCATATCACCATCCTGAATAACTGTGTTTA
TTAACTCTAATGGTGGACAATCACCTGGATTTACTGCAACATTGGTACATGGGGATCC
TTTGCCCCAGTGTTCCCCTATAGGTGGTTTGCAACCAATTAAACACAATTGTGTTTGT
TTGTAATCCATAGATATACATTCTCTATTATCCACACCTGCATTTGCTGCATAAGCACTA
GCATTTTCTGTG**C**CATCCAATTTATTTAATAAAGGATGGCCACTAATGCCCACACCTA
ATGGCTGACC

#47 AAGTCCATAGCACCAAAGCCAGTATGAACCATATCACCATCCTGAATAACTGTGTTTA
TTAACTCTAATGGTGGACAATCACCTGGATTTACTGCAACATTGGTACATGGGGATCC
TTTGCCCCAGTGTTCCCCTATAGGTGGTTTGCAACCAATTAAACACAATTGTGTTTGT
TTGTAATCCATAGATATACATTCTCTATTATCCACACCTGCATTTGCTGCATAAGCACTA
GCATTTTCTGT**AC**CATCCAATTTATTTAATAAAGGATGGCCACTAATGCCCACACCTA
ATGGCTGACC

#48 AAGTCCATAGCACCAAAGCCAGTATGAACCATATCACCATCCTGAATAACTGTGTTTA
TTAACTCTAATGGTGGACAATCACCTGGATTTACTGCAACATTGGTACATGGGGATCC
TTTGCCCCAGTGTTCCCCTATAGGTGGTTTGCAACCAATTAAACACAATTGTGTTTGT
TTGTAATCCATAGATATACATTCTCTATTATCCACACCTGCATTTGCTGCATAAGCACTA
GCATTTTCTGT**ACT**ATCCAATTTATTTAATAAAGGATGGCCACTAATGCCCACACCTA
ATGGCTGACC

#49 AAGTCCATAGCACCAAAGCCAGTATGAACCATATCACCATCCTGAATAACTGTGTTTA
TTAACTCTAATGGTGGACAATCACCTGGATTTACTGCAACATTGGTACATGGGGATCC
TTTGCCCCAGTGTTCCCCTATAGGTGGTTTGCAACCAATTAAACACAATTGTGTTTGT
TTGTAATCCATAGATATACATTCTCTATTATCCACACCTGCATTTGCTGCATAAGCACTA
GCATTTTCTGTGTCA**C**CCAATTTATTTAATAAAGGATGGCCACTAATGCCCACACCTA
ATGGCTGACC

#50 AAGTCCATAGCACCAAAGCCAGTATGAACCATATCACCATCCTGAATAACTGTGTTTA

TTAACTCTAATGGTGGACAATCACCTGGATTTACTGCAACATTGGTACATGGGGATCC
TTTGCCCCAGTGTTCCCCTATAGGTGGTTTGCAACCAATTAAACACAATTGTGTTTGT
TTGTAATCCATAGATATACATTCTCTATTATCCACACCTGCATTTGCTGCATAAGCACTA
GCATTTTCTGTGTCGCCCAATTTATTTAATAAAGGATGGCCACTAATGCCCACACCTA
ATGGCTGACC

#51 AAGTCCATAGCACCAAAGCCAGTATGAACCATATCACCATCCTGAATAACTGTGTTTA
TTAACTCTAATGGTGGACAATCACCTGGATTTACTGCAACATTGGTACATGGGGATCC
TTTGCCCCAGTGTTCCCCTATAGGTGGTTTGCAACCAATTAAACACAATTGTGTTTGT
TTGTAATCCATAGATATACATTCTCTATTATCCACACCTGCATTTGCTGCATAAGCACTA
GCATTTTCTGTGTCGCTCAATTTATTTAATAAAGGATGGCCACTAATGCCCACACCTA
ATGGCTGACC

#52 AAGTCCATAGCACCAAAGCCAGTATGAACCATATCACCATCCTGAATAACTGTGTTTA
TTAACTCTAATGGTGGACAATCACCTGGATTTACTGCAACATTGGTACATGGGGATCC
TTTGCCCCAGTGTTCCCCTATAGGTGGTTTGCAACCAATTAAACACAATTGTGTTTGT
TTGTAATCCATAGATATACATTCTCTATTATCCACACCTGCATTTGCTGCATAAGCACTA
GCATTTTCTGTGTCATCCGATTTATTTAATAAAGGATGGCCACTAATGCCCACACCTA
ATGGCTGACC

#53 AAGTCCATAGCACCAAAGCCAGTATGAACCATATCACCATCCTGAATAACTGTGTTTA
TTAACTCTAATGGTGGACAATCACCTGGATTTACTGCAACATTGGTACATGGGGATCC
TTTGCCCCAGTGTTCCCCTATAGGTGGTTTGCAACCAATTAAACACAATTGTGTTTGT
TTGTAATCCATAGATATACATTCTCTATTATCCACACCTGCATTTGCTGCATAAGCACTA
GCATTTTCTGTGTCATCTGATTTATTTAATAAAGGATGGCCACTAATGCCCACACCTA
ATGGCTGACC

#54 AAGTCCATAGCACCAAAGCCAGTATGAACCATATCACCATCCTGAATAACTGTGTTTA
TTAACTCTAATGGTGGACAATCACCTGGATTTACTGCAACATTGGTACATGGGGATCC
TTTGCCCCAGTGTTCCCCTATAGGTGGTTTGCAACCAATTAAACACAATTGTGTTTGT
TTGTAATCCATAGATATACATTCTCTATTATCCACACCTGCATTTGCTGCATAAGCACTA
GCATTTTCTGTGTCATCTGGTTTATTTAATAAAGGATGGCCACTAATGCCCACACCTA
ATGGCTGACC

#55 AAGTCCATAGCACCAAAGCCAGTATGAACCATATCACCATCCTGAATAACTGTGTTTA
TTAACTCTAATGGTGGACAATCACCTGGATTTACTGCAACATTGGTACATGGGGATCC
TTTGCCCCAGTGTTCCCCTATAGGTGGTTTGCAACCAATTAAACACAATTGTGTTTGT
TTGTAATCCATAGATATACATTCTCTATTATCCACACCTGCATTTGCTGCATAAGCACTA
GCATTTTCTGTGTCATCCAATCTATTTAATAAAGGATGGCCACTAATGCCCACACCTA
ATGGCTGACC

#56 AAGTCCATAGCACCAAAGCCAGTATGAACCATATCACCATCCTGAATAACTGTGTTTA
TTAACTCTAATGGTGGACAATCACCTGGATTTACTGCAACATTGGTACATGGGGATCC
TTTGCCCCAGTGTTCCCCTATAGGTGGTTTGCAACCAATTAAACACAATTGTGTTTGT
TTGTAATCCATAGATATACATTCTCTATTATCCACACCTGCATTTGCTGCATAAGCACTA
GCATTTTCTGTGTCATCCAACCTATTTAATAAAGGATGGCCACTAATGCCCACACCTA
ATGGCTGACC

#57 AAGTCCATAGCACCAAAGCCAGTATGAACCATATCACCATCCTGAATAACTGTGTTTA
TTAACTCTAATGGTGGACAATCACCTGGATTTACTGCAACATTGGTACATGGGGATCC
TTTGCCCCAGTGTTCCCCTATAGGTGGTTTGCAACCAATTAAACACAATTGTGTTTGT
TTGTAATCCATAGATATACATTCTCTATTATCCACACCTGCATTTGCTGCATAAGCACTA
GCATTTTCTGTGTCATCCAACCCATTTAATAAAGGATGGCCACTAATGCCCACACCT
AATGGCTGACC

#58 AAGTCCATAGCACCAAAGCCAGTATGAACCATATCACCATCCTGAATAACTGTGTTTA
TTAACTCTAATGGTGGACAATCACCTGGATTTACTGCAACATTGGTACATGGGGATCC
TTTGCCCCAGTGTTCCCCTATAGGTGGTTTGCAACCAATTAAACACAATTGTGTTTGT
TTGTAATCCATAGATATACATTCTCTATTATCCACACCTGCATTTGCTGCATAAGCACTA

GCATTTTCTGTGTCATCCAATTTACTTAATAAAGGATGGCCACTAATGCCCACACCTA
ATGGCTGACC

#59 AAGTCCATAGCACCAAAGCCAGTATGAACCATATCACCATCCTGAATAACTGTGTTTA
TTAACTCTAATGGTGGACAATCACCTGGATTTACTGCAACATTGGTACATGGGGATCC
TTTGCCCCAGTGTTCCCCTATAGGTGGTTTGCAACCAATTAAACACAATTGTGTTTGT
TTGTAATCCATAGATATACATTCTCTATTATCCACACCTGCATTTGCTGCATAAGCACTA
GCATTTTCTGTGTCATCCAATTTGCTTAATAAAGGATGGCCACTAATGCCCACACCTA
ATGGCTGACC

#60 AAGTCCATAGCACCAAAGCCAGTATGAACCATATCACCATCCTGAATAACTGTGTTTA
TTAACTCTAATGGTGGACAATCACCTGGATTTACTGCAACATTGGTACATGGGGATCC
TTTGCCCCAGTGTTCCCCTATAGGTGGTTTGCAACCAATTAAACACAATTGTGTTTGT
TTGTAATCCATAGATATACATTCTCTATTATCCACACCTGCATTTGCTGCATAAGCACTA
GCATTTTCTGTGTCATCCAATTTGCCTAATAAAGGATGGCCACTAATGCCCACACCTA
ATGGCTGACC

#61 AAGTCCATAGCACCAAAGCCAGTATGAACCATATCACCATCCTGAATAACTGTGTTTA
TTAACTCTAATGGTGGACAATCACCTGGATTTACTGCAACATTGGTACATGGGGATCC
TTTGCCCCAGTGTTCCCCTATAGGTGGTTTGCAACCAATTAAACACAATTGTGTTTGT
TTGTAATCCATAGATATACATTCTCTATTATCCACACCTGCATTTGCTGCATAAGCACTA
GCATTTTCTGTGTCATCCAATTTATTTGATAAAGGATGGCCACTAATGCCCACACCTA
ATGGCTGACC

#62 AAGTCCATAGCACCAAAGCCAGTATGAACCATATCACCATCCTGAATAACTGTGTTTA
TTAACTCTAATGGTGGACAATCACCTGGATTTACTGCAACATTGGTACATGGGGATCC
TTTGCCCCAGTGTTCCCCTATAGGTGGTTTGCAACCAATTAAACACAATTGTGTTTGT
TTGTAATCCATAGATATACATTCTCTATTATCCACACCTGCATTTGCTGCATAAGCACTA
GCATTTTCTGTGTCATCCAATTTATTCGATAAAGGATGGCCACTAATGCCCACACCTA
ATGGCTGACC

#63 AAGTCCATAGCACCAAAGCCAGTATGAACCATATCACCATCCTGAATAACTGTGTTTA
TTAACTCTAATGGTGGACAATCACCTGGATTTACTGCAACATTGGTACATGGGGATCC
TTTGCCCCAGTGTTCCCCTATAGGTGGTTTGCAACCAATTAAACACAATTGTGTTTGT
TTGTAATCCATAGATATACATTCTCTATTATCCACACCTGCATTTGCTGCATAAGCACTA
GCATTTTCTGTGTCATCCAATTTATTCGGTAAAGGATGGCCACTAATGCCCACACCTA
ATGGCTGACC

* The target region of CRISPR-Cas12a is underlined, and the three rules for nucleotide substitution are highlighted in red, green, and blue, respectively.

Supplementary Table 8. Sequences used in the CRISPR assay and qPCR assay for detection of HPV plasmid and clinical samples of HPV.

Name	***GenBank***
HPV 16 L1 plasmid	K02718.1
HPV 18 L1 plasmid	MF288726.1
HPV 31 L1 plasmid	J04353.1
HPV 33 L1 plasmid	M12732.1
HPV 35 L1 plasmid	X74477.1
HPV 39 L1 plasmid	KC470236.1
HPV 45 L1 plasmid	X74479.1
HPV 51 L1 plasmid	KU298904.1
HPV 52 L1 plasmid	X74481.1
HPV 56 L1 plasmid	X74483.1
HPV 58 L1 plasmid	KU550602.1
HPV 59 L1 plasmid	X77858.1
HPV 66 L1 plasmid	LR861902.1
HPV 68 L1 plasmid	X67161.1

Supplementary Table 9. Nucleotide sequences of universal RPA primer pairs.

Name	***Sequence (5’→3’)***
Pair1_FP	GGNCAGCCWTTAGGTGTTGGYMTWAGTGGTCATCC
Pair1_RP	TCCATRGCSCCASATCCTGTRTCHACCATRTCMCC
Pair2_FP	CAGGGYCAYAAYAATGGYATTTGYTGGSRYAATCA
Pair2_RP	TGAAAAATAAAYTGTAAATCATATTCCTCCMMATG
F1	GGNCAGCCTTAGGTGTTGGYMTWAGTGGTCATCC
F2	GNCAGCCWTTAGGTGTTGGYMTWAGTGGTCATCCA
F3	NCAGCCWTTAGGTGTTGGYMTWAGTGGTCATCCAT
F4	CAGCCWTTAGGTGTTGGYMTWAGTGGTCATCCATT
F1.1	GGNCAGCCWTTAGGTGTTGGYMTWAGTGGTCATC
F1.2	GGNCAGCCWTTAGGTGTTGGYMTWAGTGGTCAT
F1.3	GGNCAGCCWTTAGGTGTTGGYMTWAGTGGTCA
F1.4	GGNCAGCCWTTAGGTGTTGGYMTWAGTGGTC
F1.5	GGNCAGCCWTTAGGTGTTGGYMTWAGTGGT
R1	ATRGCSCCASATCCTGTRTCHACCATRTCMCCATC
R2	CATRGCSCCASATCCTGTRTCHACCATRTCMCCAT
R3	CCATRGCSCCASATCCTGTRTCHACCATRTCMCCA
R4	TCCATRGCSCCASATCCTGTRTCHACCATRTCMCC
R5	GTCCATRGCSCCASATCCTGTRTCHACCATRTCMC
R6	AGTCCATRGCSCCASATCCTGTRTCHACCATRTCM
R7	AAGTCCATRGCSCCASATCCTGTRTCHACCATRTC
R7.1	AAGTCCATRGCSCCASATCCTGTRTCHACCATRT
R7.2	AAGTCCATRGCSCCASATCCTGTRTCHACCATR
R7.3	AAGTCCATRGCSCCASATCCTGTRTCHACCAT
R7.4	AAGTCCATRGCSCCASATCCTGTRTCHACCA
R7.5	AAGTCCATRGCSCCASATCCTGTRTCHACC

* FP/F: forward primer; RP/R: reverse primer

Supplementary Table 10. Nucleotide sequences of crRNAs.

Name	***Sequence (5'→3')***
Seq1	UAAUUUCUACUAAGUGUAGAU AUGGAUUAUAAACAAACACA
Seq2	UAAUUUCUACUAAGUGUAGAU GACUGUCCUCCUUUGGAACU
Seq3	UAAUUUCUACUAAGUGUAGAU UAAUAAUGUUAUAGAAGAUAG
Seq4	UAAUUUCUACUAAGUGUAGAU UAAUAGGCUGGAUGAUACUGAAA
Seq1-mo1	UAAUUUCUACUAAGUGUAGAU AUGGAUUUUAAACAAACACA
Seq1-mo2	UAAUUUCUACUAAGUGUAGAU AUGGAUUCUAAACAAACACA
Seq1-mo3	UAAUUUCUACUAAGUGUAGAU AUGGAUUGUAAACAAACACA
Seq4-mo1	UAAUUUCUACUAAGUGUAGAU UAAUAGGCAGGAUGAUACUGAAA
Seq4-mo2	UAAUUUCUACUAAGUGUAGAU UAAUAGGCCGGAUGAUACUGAAA
Seq4-mo3	UAAUUUCUACUAAGUGUAGAU UAAUAGGCGGGAUGAUACUGAAA
Seq4-mo4	UAAUUUCUACUAAGUGUAGAU AAUAGGCUGGAUGAUACUGAAA
Seq4-mo5	UAAUUUCUACUAAGUGUAGAU AUAGGCUGGAUGAUACUGAAA
Seq4-mo6	UAAUUUCUACUAAGUGUAGAU UAGGCUGGAUGAUACUGAAA
crRNA A	UAAUUUCUACUAAGUGUAGAU UAGGGGAACACUGGGGCAAA
crRNA B	UAAUUUCUACUAAGUGUAGAU CCACGUCUAAUGUUUCUGAG
crRNA C	UAAUUUCUACUAAGUGUAGAU UAAUAGGCAGGAUGAUACUGAAA
crRNA D	UAAUUUCUACUAAGUGUAGAU AUAGGCUGGAUGACACUGAGG
crRNA E	UAAUUUCUACUAAGUGUAGAU AUGGAUUUUAAACAAACACA
crRNA F	UAAUUUCUACUAAGUGUAGAU UAAUCCAUAGAUAAGCAUUCC
crRNA G	UAAUUUCUACUAAGUGUAGAU UUAGAAUUUUCAGUAUCAUCC
crRNA H	UAAUUUCUACUAAGUGUAGAU UAAGCCUACUACUGUGGUUCA
crRNA I	UAAUUUCUACUAAGUGUAGAU CUGUGUCAUCAUAUUUAUUAA
crRNA J	UAAUUUCUACUAAGUGUAGAU AUGACACUGAAAACUCUAAUA

Supplementary Table 11. Primers and probes of PCR tests.

Name	***Sequence (5'→3')***
HPV16 FWD	GGTCGTGGTCAGCCATTAG
HPV16 REV	TCTCTATTATCCACACCTGCATTT
HPV16 PRB	/56-FAM/TGTGGGCAT/ZEN/TAGTGGCCATCCTTT/3IABkFQ/
HPV18 FWD	CTGGCAGCTGTGTGTATTCT
HPV18 REV	GTTATGACCCTGTGCCTTATGT
HPV18 PRB	/56-FAM/TGGCTCTAT/ZEN/TGTTACCTCTGACTCCCA/3IABkFQ/
HPV31 FWD	AATAGATCAGGCACGGTTGG
HPV31 REV	CCGCTAGGTGTAGGAAAGTATG
HPV31 PRB	/56-FAM/AGGCTCCGG/ZEN/TTCAACAGCTACTTT/3IABkFQ/
HPV33 FWD	GTGTAGGCCTTGAAATAGGTAGAG
HPV33 REV	CGGTTGTCCAGGATACTTGTT
HPV33 PRB	/56-FAM/TAGGCGTTG/ZEN/GCATAAGTGGTCATCC/3IABkFQ/
HPV35 FWD	GGCTGGAACTGTAGGTGAAA
HPV35 REV	CATAGAGCCACTAGGAGTAGGA
HPV35 PRB	/56-FAM/ACTAGGCAA/ZEN/TGTGCCAGTGGTACC/3IABkFQ/
HPV39 FWD	CATTCCAAAGGTGTCTGCATATC
HPV39 REV	CCCTACACAAGCCCATACTAAA
HPV39 PRB	/56-FAM/CGCGTGACA/ZEN/TTGCCCGATCCTAATA/3IABkFQ/
HPV45 FWD	CCAAGGGCACACTTTGTAAAC
HPV45 REV	CCCATAACCTGTATCCACCATATC
HPV45 PRB	/56-FAM/AAAGGAGGA/ZEN/CAGTCACCAGGTTGC/3IABkFQ/
HPV51 FWD	GCCTTAGTGGTCATCCCTTATT
HPV51 REV	GGTGGAGCACAGCCTATTATAC
HPV51 PRB	/56-FAM/TCACGCATA/ZEN/GCAAATGGCAATGCA/3IABkFQ/
HPV52 FWD	TGTCTCGCACAAGCATCTATTA
HPV52 REV	CCAGACACCTTGGGAACTAAA
HPV52 PRB	/56-FAM/TGCAGGCAG/ZEN/TTCTCGATTACTAACAGT/3IABkFQ/
HPV56 FWD	AGTTTGGGCTTCCAGATACTAAT
HPV56 REV	CACTTAGCCCAGCACCTAAA
HPV56 PRB	/56-FAM/AGTGTGGGC/ZEN/ATGTGTAGGTTTGGA/3IABkFQ/
HPV58 FWD	GTTCCCAAGGTATCAGGCTTAC
HPV58 REV	TACACATGCCCAGACCAAAC
HPV58 PRB	/56-FAM/AGGGTGCGT/ZEN/TTACCTGATCCCAAT/3IABkFQ/
HPV59 FWD	GTATGTCACCCGTACCAGTATTT
HPV59 REV	CCTGTCTACCATTACCACCTTTAG
HPV59 PRB	/56-FAM/ACGCAGGCA/ZEN/GTTCCAGACTTCTT/3IABkFQ/
HPV66 FWD	GCCTTCCTGATCCATCTTTCT
HPV66 REV	CCACTTAACCCAGCACCTAAA
HPV66 PRB	/56-FAM/CGTTTGGTA/ZEN/TGGGCCTGTGTAGGT/3IABkFQ/
HPV68 FWD	TGTCCTCTGACTCCCAGTTAT

HPV68 REV	CGTTGTATCCACAACGGTAAGA
HPV68 PRB	/56-FAM/AGGCACAGG/ZEN/GACACAACAATGGTA/3IABkFQ/
GAPDH Primer1	CATGTGGGCCATGAGGTCCACCAC
GAPDH Primer2	TGAAGGTCGGAGTCAACGGATTTGG
GAPDH Probe	/56-FAM/CAAGCTTCC/ZEN/CGTTCTCAGCC/3IABkFQ/

***** The primers and probes were tested to ensure no cross-reactivity.

Supplementary Table 12. Sequences used in the development of the assay of HCV detection.

Name	***Sequence (5’→3’)***
HCV GT1	ACACGGAAGATGTCGTGTGCTGCTCAATGTCTTATTCCTGGACAGGCGCACTC<br>GTCACCCCGTGCGCTGCGGAAGAACAAAAACTGCCCATCAACGCACTGAGCA<br>ACTCGTTGCTACGCCATCACAATCTGGTGTATTCCACCACTTCACGCAGTGCTT<br>GCCAAAGGCAGAAGAAAGTCACATTTGACAGACTGCAAGTTCTGGACAGCC
HCV GT2	AGGACGACTCCGTCGTGTGCTGCTCCATGTCATACTCCTGGACCGGGGCTCTA<br>ATAACTCCTTGTAGTCCCGAAGAGGAGAAGTTACCGATTAACCCCTTGAGCAA<br>CTCCCTGTTGCGATATCACAACAAGGTGTACTGTACCACAACAAAGAGCGCCT<br>CACTAAGGGCTAAAAAGGTAACTTTTGATAGGATGCAAGTGCTCGACTCCT
HCV GT3	AGGAGCAGAGCGTGGTCTGCTGCTCTATGTCGTATTCTTGGACCGGCGCCCTG<br>ATAACACCATGTAGTGCTGAGGAAGAGAAACTGCCCATCAGCCCACTCAGCA<br>ACTCCCTGTTGAGACATCATAACCTAGTCTATTCAACGTCGTCTAGAAGCGCTT<br>CTCAGCGTCAGAAGAAGGTTACCTTCGATAGACTGCAGGTGCTCGACGACC
HCV GT4	GATCGGAGGACGTCGTGTGCTGTTCGATGTCATACTCGTGGACTGGGGCGCTT<br>GTAACACCTTGCGCGGCTGAAGAATCAAAGCTGCCAATTAGCCCCCTGAGCAA<br>TTCACTTTTGCGCCATCACAATATGGTGTATGCCACGACCACCCGTTCTGCTGT<br>GACACGGCAGAAGAAGGTGACCTTCGACCGCCTGCAGGTGGTGGACAGTA
HCV GT5	GTGAGGACAACGTGGTGTGTTGCTCCATGTCATACACCTGGACTGGGGCGCTC<br>ATCACCCCTTGCTCTGCTGAAGAGGAAAAATTACCCATCAATCCCTTAAGCAA<br>CACCTTATTACGCCACCACAATCTTGTGTACTCCACCTCCTCTCGGAGTGCGGG<br>TCTGAGGCAGAAAAAGGTCACTTTTGACAGGCTACAAGTCCTCGACGACC
HCV GT6	AAGAGGACGATGTCGTCTGTTGCTCTATGTCATACACTTGGACGGGGGCCTTG<br>ATCACACCATGCGCTGCTGAGGAGGAGAAATTGCCAATAAACCCTTTGAGCA<br>ATTCTCTCATAAGACACCACAACATGGTCTACTCCACGACATCACGTAGCGCC<br>GGCCTTCGCCAGAAGAAAGTCACATTTGACAGACTACAGGTAGTTGACCAAC
Seq	UAAUUUCUACUAAGUGUAGAUCCUCUUCAGCAGUGCAAGGU
Seq-mod1 (crRNA B)	UAAUUUCUACUAAGUGUAGAUCCUCUUCAGCAGUACAAGGU
Seq-mod2	UAAUUUCUACUAAGUGUAGAUCCUCUUCAGCACUGCAAGGU
crRNA A	UAAUUUCUACUAAGUGUAGAUGCAAGCACUGCGUGAAGUGG
crRNA C	UAAUUUCUACUAAGUGUAGAUCUUCCUCAGCACUACAUGGU

References

[1] T. L. Bailey, C. Elkan, **1994**.

[2] aZ. Li, X. Ding, K. Yin, L. Avery, E. Ballesteros, C. Liu, *Biosensors and Bioelectronics* **2022**, *199*, 113865; bJ. Zhang, Z. Li, C. Guo, X. Guan, L. Avery, D. Banach, C. Liu, *Angewandte Chemie International Edition* **2024**, *63*, e202403123.

[3] F. Madeira, M. Pearce, A. R. Tivey, P. Basutkar, J. Lee, O. Edbali, N. Madhusoodanan, A. Kolesnikov, R. Lopez, *Nucleic acids research* **2022**, *50*, W276-W279.

[4] M. D. Ruopp, N. J. Perkins, B. W. Whitcomb, E. F. Schisterman, *Biometrical Journal: Journal of Mathematical Methods in Biosciences* **2008**, *50*, 419-430.

[5] M. A. Larkin, G. Blackshields, N. P. Brown, R. Chenna, P. A. McGettigan, H. McWilliam, F. Valentin, I. M. Wallace, A. Wilm, R. Lopez, *bioinformatics* **2007**, *23*, 2947-2948.

[6] K. Katoh, D. M. Standley, *Molecular biology and evolution* **2013**, *30*, 772-780.

[7] T. L. Bailey, C. Elkan, in *Ismb, Vol. 3*, **1995**, pp. 21-29.
